# Solvent Induced Helix Folding of Defined Indolenine Squaraine Oligomers

**DOI:** 10.1002/chem.202101063

**Published:** 2021-05-07

**Authors:** Arthur Turkin, Marco Holzapfel, Mohit Agarwal, David Fischermeier, Roland Mitric, Ralf Schweins, Franziska Gröhn, Christoph Lambert

**Affiliations:** ^1^ Institut für Organische Chemie Universität Würzburg Am Hubland 97074 Würzburg Germany; ^2^ Department of Chemistry and Pharmacy & Interdisciplinary Center for Molecular Materials (ICMM) and Bavarian Polymer Institute (BPI) University of Erlangen-Nürnberg Egerlandstraße 3 91058 Erlangen Germany; ^3^ Institut Max von Laue - Paul Langevin (ILL), DS / LSS 71, Avenue des Martyrs - CS 20156 38042 Grenoble Cedex 9 France; ^4^ Institut für Physikalische und Theoretische Chemie Universität Würzburg Am Hubland 97074 Würzburg Germany; ^5^ Center for Nanosystems Chemistry Universität Würzburg Am Hubland 97074 Würzburg Germany

**Keywords:** dye chemistry, solvent effects, superstructure, supramolecular folding, UV/Vis spectroscopy

## Abstract

A protecting group strategy was employed to synthesise a series of indolenine squaraine dye oligomers up to the nonamer. The longer oligomers show a distinct solvent dependence of the absorption spectra, that is, either a strong blue shift or a strong red shift of the lowest energy bands in the near infrared spectral region. This behaviour is explained by exciton coupling theory as being due to H‐ or J‐type coupling of transition moments. The H‐type coupling is a consequence of a helix folding in solvents with a small Hansen dispersity index. DOSY NMR, small angle neutron scattering (SANS), quantum chemical and force field calculations agree upon a helix structure with an unusually large pitch and open voids that are filled with solvent molecules, thereby forming a kind of clathrate. The thermodynamic parameters of the folding process were determined by temperature dependent optical absorption spectra.

## Graphical abstract

A series of defined indolenine squaraine dye oligomers up to the nonamer were synthesised via iterative cross coupling. Depending on the solvent either a random coil (J‐type) or a loose helix (H‐type) is formed. Structural and folding properties were examined via optical spectroscopy, DOSY NMR, SANS and computations.

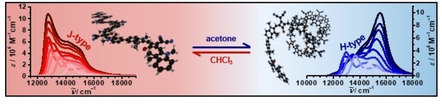

## Introduction

The helix structure is one of the most important supramolecular elements in chemistry.^[1]^ It plays a decisive role in the superstructure of proteins, polysaccharides, DNA, and RNA but was also found in purely artificial supramolecular assemblies of low molecular weight molecules, in coordination polymers and also in oligomers and polymers of covalently linked organic monomers.^[2]^ Among the latter are polyisocyanides,^[3]^ polyisocyanates,^[4]^ *meta*‐phenylene oligomers,^[5]^ oligo(*m*‐phenylene ethynylene)s,^[6]^ oligo‐*β*‐aminoacids,^[7]^ heterocyclic^[8]^ and non‐heterocyclic^[9]^ aromatic amide oligomers, just to name a few parent structures. All these oligomers/polymers may adopt helix structures by a folding process.^[10]^ In many cases the helix folding depends on specific conditions such as temperature,^[11]^ solvent,[^6^, ^12^] (metal) ion concentration,^[13]^ pH‐value,^[14]^ hydrogen bonding^[15]^ or chiral templates such as carbohydrates.[^15b^, ^16^] However, in only very few cases the parent monomer units in the main chain of the folding oligomers/polymers are dyes with an intrinsic strong absorption in the visible or near infrared (NIR) optical range.^[17]^ The reason for that is the lack of suitable dyes which possess a structure that is intrinsically prone to helix formation. T. Nebeshima et al. described a BODIPY trimer that folds to one helix turn upon titration with cesium ions.^[13a]^ Werz. et al.^[18]^ also suggested a helix structure for oligo‐BODIPYs where the relative orientation of the monomers is locked by ethyl substituents. A special case is the linear *meso*‐linked oligo‐Zn‐porphyrin in which the helicity is induced by stepwise turning the porphyrin plane around the connecting axis induced by interaction of the side chain substituents.^[19]^

Though aggregates^[20]^ and polymers^[21]^ of squaraine dyes are well known, they have not yet been explored in the context of folding processes. Their optical properties[^21c^, ^22^] – a narrow and intense absorption band in the red to NIR spectral range and a high fluorescence quantum yield – make them an ideal candidate for studying modification of optical properties upon changes of the mutual orientation of chromophores within either a supramolecular aggregate or covalently bound oligomers or polymers. Thus, the control of supramolecular folding structure would enable to tune optical properties for potential applications in e. g. solar cells, sensors, biolabels, and nonlinear optics.

**Figure 1:**
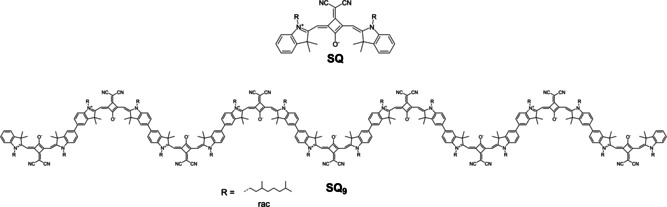
**SQ** monomer and **SQ** nonamer.

In earlier work^[21c]^ we made the puzzling observation that a polymer based on the *cisoid* squaraine **SQ** shows in some solvents such as CHCl_3_ a red‐shift of the main absorption band compared to the **SQ** monomer but in other solvents such as acetone a blue‐shift (see Figure S4 in the Supporting Information). The **SQ** monomer was described first by E. Terpetschnig et al.[^22d^, ^23^] in the late 90ies and, unlike most other squaraine dyes, possesses a bent structure (Figure 1). Following Kasha's exciton theory,^[24]^ for a linear arrangement of monomer units within a polymer strand one expects that the lowest energy exciton state is allowed but all other states within the exciton manifold are forbidden. Thus, we interpreted the observed red‐shift of the **SQ** polymer as being due to exciton interactions of the localized transition moments in a J‐type (=head‐to‐tail) manner. However, the blue‐shift in other solvents such as DMF or acetone was explained by a solvent dependent helix folding. This sounds reasonable in view of the *cisoid* structure of **SQ** which allows connecting two squaraines with an about 120° angle via a biaryl bond. The twist around the biaryl axis then controls the helix pitch in return. Calculations showed that approximately three squaraines were needed for a turn. And indeed, cyclic trimers of **SQ** which we synthesised in earlier works also indicate that for a helix turn about three monomers are necessary.[^21c^, ^25^] While for the **SQ** polymer we investigated photoinduced energy transfer processes in great detail,^[26]^ the exact structure and the folding conditions remained unexplored. However, this knowledge is necessary in order to use the folding for tuning optical properties for further applications. Therefore, in this study we will address the question of how many **SQ** monomers are necessary to form a helix, what is the helix pitch and the helix diameter and we will also give information about the folding thermodynamics. Thus, we will explore the chain length dependence^[27]^ of the optical spectroscopic properties in various solvents. We will learn that the longer **SQ** oligomers indeed form a quite unique helical structure which is – unlike practically all other known helix structure of π‐systems – very loose with a large helix pitch. In order to prove that, we will present a strategy to synthesise monodisperse **SQ** oligomers up to the nonamer (see Figure 1) which requires a delicate protecting group technique based on Suginome's^[28]^ 1,8‐diaminonaphthalene protected boronic acid, which has been applied for the synthesis of small phenylene dendrons before.^[29]^

## Results

**Figure 2:**
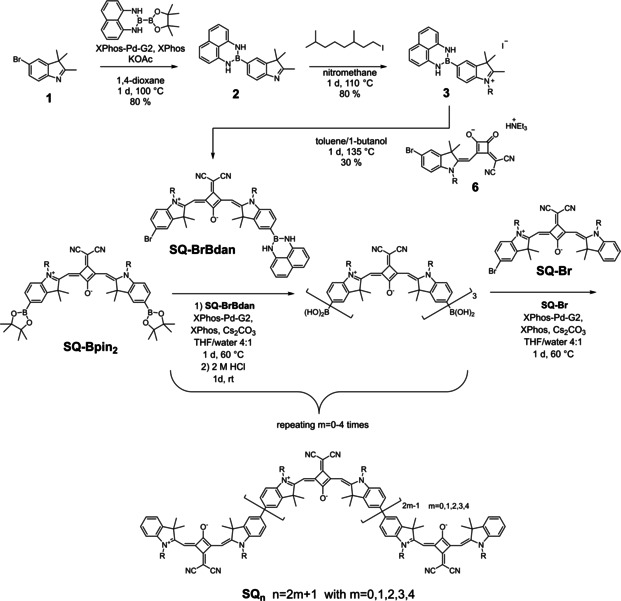
Iterative cross coupling of **SQ** oligomers using 1,8‐diaminonaphthalene as a protecting group for squaraine boronic acid

**Synthesis of Oligosquaraines SQ_n_**: The syntheses of **SQ** monomer, dimer and trimer were already reported by us recently.^[30]^ In order to synthesise longer monodisperse **SQ_n_** oligomers up to the nonamer via a Suzuki‐Miyaura coupling we employed a protecting group for boronic acids developed by Suginome et al.[^28a^, ^28c^] These authors used 1,8‐diaminonaphthalene (**dan**) to protect aryl boronic acids which can easily be deprotected in an acidic environment. Thus, starting from the symmetric squaraine pinacol boronic ester **SQ‐Bpin_2_** iterative cross coupling with **SQ‐BrBdan** at both sides yielded the odd‐numbered **SQ** oligomers, see Figure 2, lower part. Since after deprotection the oligosquaraine boronic acids are difficult to purify and to characterise, intermediates were characterised at the step of the **dan** protected boronic acids.[^29a^, ^31^] In the last step, coupling of the oligosquaraine boronic acids with **SQ‐Br** terminated the oligomer chains. Thus, the bifunctional squaraine **SQ‐BrBdan** is the key building block for chain elongation and was synthesised from the appropriate indolenine and squaric acid ester according to Figure 2, upper part. The indolenines were appended by *rac*‐3‐7‐dimethyloctyl chains to ensure solubility. The even‐numbered oligomers were prepared in a similar way as the odd‐numbered oligomers starting with a symmetrically **Bdan** substituted **SQ** dimer (see Supporting Information). Yields for deprotection and double cross coupling vary between about 20–90 % per step. The details of each synthetic step can be found in the Supporting Information.

### Absorption spectroscopy

**Figure 3:**
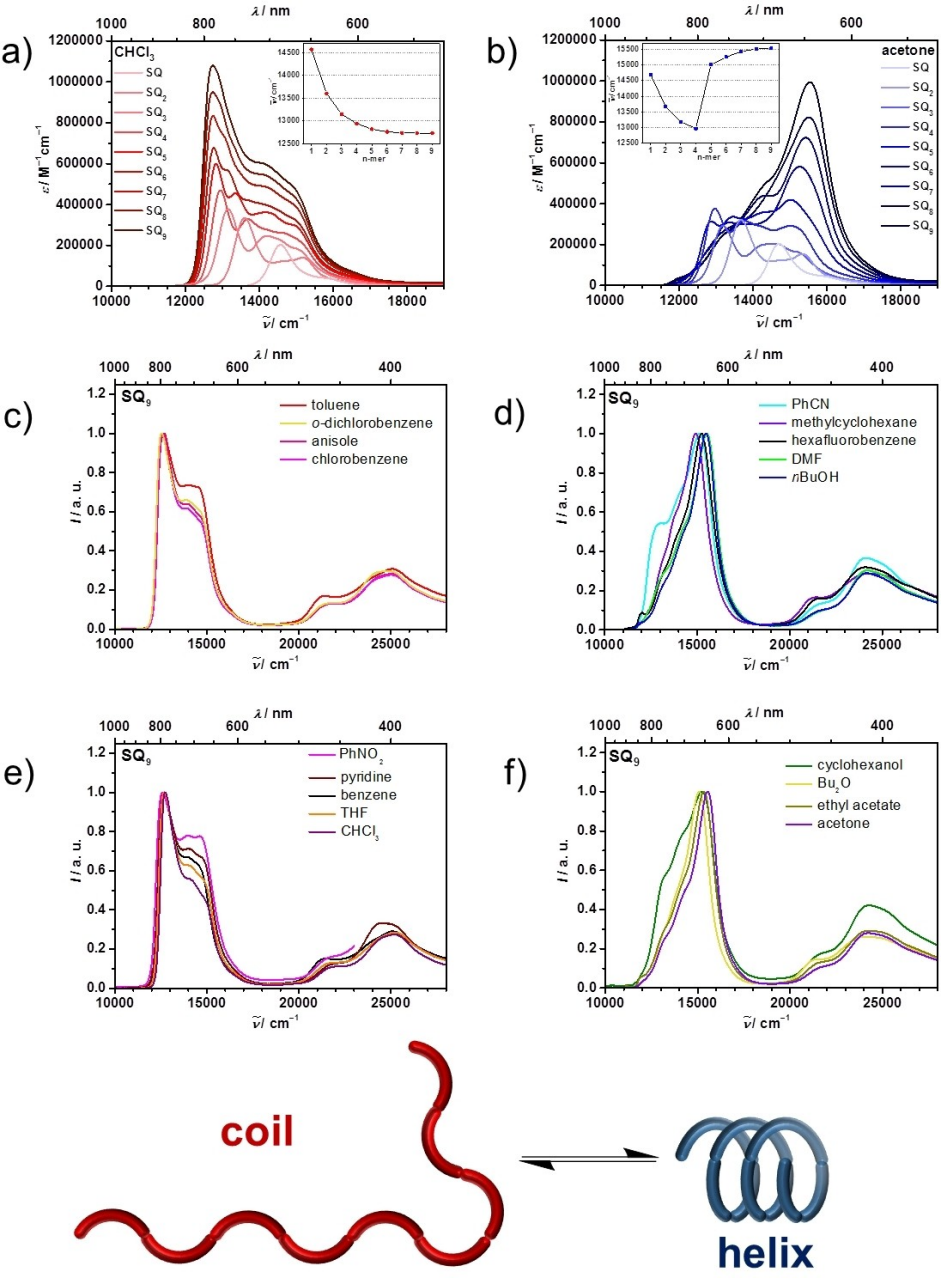
Lowest energy part of the absorption spectra of **SQ** oligomers in (a) CHCl_3,_ and (b) in acetone. The insets show the energy of the absorption maxima vs. chain length. (c) to (f) Normalised absorption spectra of **SQ_9_** in diverse solvents. The structures at the bottom illustrate the coiled and helix folding process and refer to the predominant structures present in solution for the spectra depicted on the left‐hand side (random coil) and on the right hand side (helix).

The **SQ** oligomers were dissolved in CHCl_3_ and their absorption spectra were measured, see Figure 3a. The absorption spectrum of **SQ** monomer shows an intense peak at 14300 cm^−1^ with a much smaller shoulder at higher energies caused by vibronic progression. For **SQ_2_** and **SQ_3_** the lowest energy absorption increases in intensity and shifts towards lower energy compared to **SQ**. At the higher energy side the shoulders are also more intense and structured. As recently discussed,[^30^, ^32^] this is a consequence of exciton coupling between monomer localised states in a J‐type manner, that is, the localised transition moments are arranged in a head‐to‐tail orientation. Here, the in‐phase combination of transition moments refers to the most stable eigenstate and possesses the highest intensity. The higher energy exciton eigenstates (S_2_ for **SQ_2_** and S_2_ and S_3_ for **SQ_3_**) also show some intensity which, for the S_2_ state, is caused by structural disorder, that is, rotation around the biaryl axis results in conformers where the transition moments are no longer in head‐to‐tail orientation but adopt a significant angle relative to each other. While the absorption spectra of the smaller **SQ_n_** chromophores with *n*=1–3 are almost identical irrespective of the solvents (see Figure 2a and b), the absorption spectra of the higher **SQ_n_** oligomers (*n*>3) show a strong solvent dependence that can be classified into either J‐type or H‐type aggregate behavior, depending on the shift of the most intense absorption band: in CHCl_3_ the J‐type behavior leads to an increase of intensity and bathochromic shift of the lowest energy band with increasing oligomer length. More specific, the lowest energy band shifts from 14300 cm^−1^ for **SQ** to 12700 cm^−1^ for **SQ_9_**. However, in acetone a clear H‐type behavior is seen (see Figure 3b) where the highest energy band of the exciton manifold shifts towards higher energies (e. g. to 15500 cm^−1^ for **SQ_9_**) and gains intensity with increasing chain length. Comparison with the spectra of a related polymer with *X*_n_=50 shows that in CHCl_3_ the spectra are practically identical but in acetone the absorption maximum of the polymer is still somewhat blue shifted compared to the nonamer (see Figure S4 in the Supporting Information). A plot of the squared transition dipole moment ($\mu_{\mathrm{eg}}^{2}$ =dipole strength, determined by integration over the whole exciton manifold from 11000–19000 cm^−1^) vs. oligomer length shows for both solvents a linear correlation (see Figure S1d) and S2d) in the Supporting Information) which proves that no other states besides those generated by exciton coupling are involved in this energy range.

In the broad range of tested solvents ranging from hydrocarbons, aromatic solvents, chlorinated solvents, polar aprotic solvents, alcohols, and ethers the higher **SQ** oligomers either show a clear J‐type (see Figure 3c and e) or H‐type behavior (see Figure 3d and f) for **SQ_9_**.^[33]^ There is only one exception, benzonitrile (PhCN), which displays H‐type spectra at rt but J‐type behavior at elevated temperature indicating a temperature dependent reversible folding process. Thus, in the following we concentrate on the optical properties of the **SQ** oligomers in CHCl_3_, acetone and PhCN. Since concentration dependent measurements in the range of 10^−6^–10^−8^ M in PhCN and of 10^−4^–10^−6^ M in acetone did not reveal any intermolecular aggregation effects, the different optical behavior in CHCl_3_ and acetone must be due to a different oligomer structure in the respective solvent (see Figure S3 in the Supporting Information). The most extremes conceivable are a linear chain and a helix structure. The former is expected to show J‐type behavior, the latter H‐type behavior. However, structural disorder such as the formation of rotamers around the biaryl axes might blur the pure J/H‐type spectroscopic appearance of the oligomers in the respective solvent. This interpretation is based on our earlier work on **SQ** polymers.[^21c^, ^26^] When looking at the evolution of absorption maxima in CHCl_3_ it is obvious that all oligomers adopt a chain structure, see inset in Figure 3a. This is different for acetone solutions of the oligomers. The inset in Figure 3b shows a close similarity of the energy of absorption maxima in acetone and in CHCl_3_ solution up to the tetramer. However, for the higher oligomers **SQ_5_** to **SQ_9_** the absorption maximum in acetone switches to the highest exciton state, indicating partial helix formation for **SQ_5_** and almost complete helix formation for **SQ_9_** as the spectral differences between **SQ_8_** and the latter are minor.

#### Helix folding

**Figure 4:**
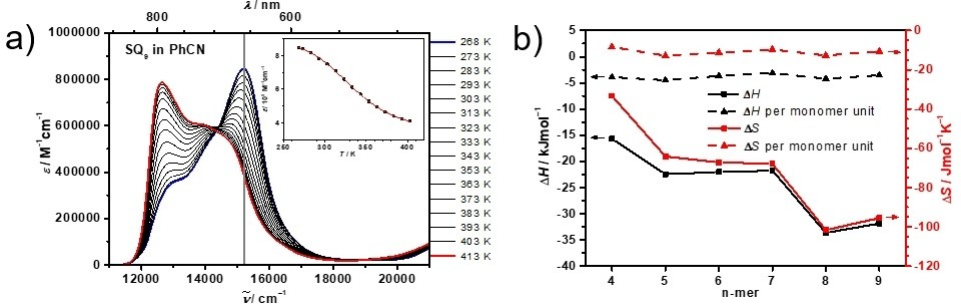
(a) Absorption spectra of **SQ_9_** in PhCN at different temperatures. Inset: Extinction coefficient at 15200 cm^−1^ (black squares) at different temperature and fit with Equation 1 (red line). (b) Enthalpy and entropy of the coil‐helix folding for the squaraine oligomers (squares) and for the oligomers divided by the number of monomer units (triangles).

While in CHCl_3_ the absorption spectra of the higher oligomers display clear J‐type behavior and in acetone distinct H‐type behavior, those in PhCN possess H‐type features but upon heating, the spectra change gradually and reversibly and display the typical J‐type behavior. This allows determining the thermodynamic parameters of the chain‐helix interconversion. In Figure 4 the temperature dependent spectra of **SQ_9_** are given as the most prominent example, those of **SQ_4_**‐**SQ_8_** can be found in Figure S8 in the Supporting Information. Clearly, at low temperature (268 K) we find H‐type spectra and at elevated temperature (413 K) J‐type spectra. An almost perfect isosbestic point proves that only two species are involved in the equilibrium. Therefore, we fitted the temperature dependent extinction coefficient at the maximum absorption of the H‐band at 15200 cm^−1^ by assuming a simple two‐state interconversion equilibrium C$\overset{}{\underset{}{↔}}$ H with Equation 1, see inset in Figure 4a), thus, we assumed a fully cooperative coil‐helix transformation.^[34]^ Here, *ϵ*_H_ is the extinction coefficient of the pure helix (H) and *ϵ*_C_ that of the pure chain (C) structure.

(1) $$\frac{OD\left(\lambda \right)}{cd}=\frac{K}{1+K}\epsilon_{H}+\left(\frac{1}{1+K}\right)\epsilon_{C}\;\mathrm{with}\;K=exp\left(-\frac{\Delta H}{RT}+\frac{\Delta S}{R}\right)$$

The thereby evaluated enthalpy Δ*H* ranges between −15.7 kJ mol^−1^ for the tetramer to −33.7 kJ mol^−1^ for the octamer, the respective entropy between −33.3 J mol^−1^ K^−1^ and −101.6 J mol^−1^ K^−1^ (see Figure 4b). Thus, helix formation is exothermic but disfavored by entropy, as anticipated for a more ordered structure. A closer inspection of the enthalpy and entropy, however, reveals a distinct step‐like behavior, that is, these values are very similar for the pentamer to heptamer but distinctly more negative for the octamer and nonamer. The highest values are obtained for the tetramer. We assume that this is due to the number of helix turns that may form in each oligomer. Assuming about three monomers per helix turn (see above), the pentamer to heptamer may form two turns while the octamer and nonamer may form three turns. In Figure 4b), the corresponding enthalpy and entropy is also normalised by the number of monomers which gives an enthalpy of roughly −4 kJ mol^−1^ and an entropy of −11 J mol^−1^ K^−1^ per monomer. The Gibbs free energy is on the order of −2 to −6 kJ mol^−1^ depending on oligomer length. As we observe either a clear J‐ or H‐type behaviour in all other solvents and no temperature dependence of the spectra, the absolute values of the Gibbs free energy are expected to be much larger there. Indeed, measuring absorption spectra of **SQ_9_** in CHCl_3_/acetone mixtures ranging from pure CHCl_3_ to pure acetone in 5 %vol fraction steps (see Figure S10 in the Supporting Information) yielded equilibrium constants and, thereby, Gibbs free energies referring to each particular solvent mixture.[^11^, ^34^] Extrapolating the Δ*G* dependence to pure CHCl_3_ and to pure acetone gave Δ*G*=+13.4 kJ mol^−1^ and −12.4 kJ mol^−1^, respectively, for the helix formation process, compared to −3.4 kJ mol^−1^ in PhCN, see Figure S11 in the Supporting Information.

### Helix structure

**Figure 5:**
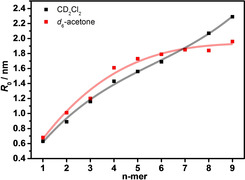
Hydrodynamic radii of **SQ** oligomers in CD_2_Cl_2_ and in acetone‐*d*_6_ obtained from the DOSY measured diffusion coefficients via the Stokes‐Einstein equation. The solid lines are cubic fit functions and serve only as a guide to the eye.

While optical spectra and thermodynamic considerations speak for a helix formation of longer **SQ** oligomers in acetone, the geometric dimensions need to be elucidated. Simple 1D and 2D NMR methods proved to be unhelpful as the oligoquaraines show rather complex spectra in CD_2_Cl_2_ or acetone‐*d*_6_ and strongly temperature dependent spectra in PhCN‐*d*_5_ (see Figure S45–47 in the Supporting Information). For this reason, we performed diffusion ordered DOSY NMR measurements in acetone‐*d*_6_ and in CD_2_Cl_2_ (see Figure S48–56 in the Supporting Information). The latter was chosen as the squaraine solutions showed somewhat sharper and better resolved signals in CD_2_Cl_2_ than in CDCl_3_. Nonetheless, the optical spectroscopic behavior is almost identical in the two solvents. From the DOSY NMR measurements we calculated the hydrodynamic radii *R*_0_ of the oligomers from the corresponding diffusion coefficients *D* by the Stokes‐Einstein equation (Equation S1 in the Supporting Information). While different equations^[35]^ according to different hypothetical geometries can be used to calculate a hydrodynamic radius, here we resort to the simple Stokes‐Einstein equation which will at least yield comparable results within the **SQ** oligomer series within one solvent. The results are given in Figure 5. Here we can see that in CD_2_Cl_2_ the hydrodynamic radius increases with the number of monomer units. This is expected for random coils whose size, on average, increases with the chain length. In acetone, a similar trend is followed up to the tetramer. However, beginning with the pentamer, the curve flattens considerably, that is, the structure of the longer oligomers starts to be more compact due to limited one dimensional growth, in agreement with the suggested helix structure.

In order to gain more precise information on the nanoscale structure we performed small angle neutron scattering (SANS) measurements on **SQ_8_** in acetone‐*d*_6_ (see Figures S57 and S58 in the Supporting Information). The best fit of the scattering data was obtained for a flexible cylinder model with a cross‐section radius *R*_c_=(0.9±0.1) nm, a total length of *L*=(8.5±0.5) nm, and a Kuhn segment length of *l*_K_=(2.1±0.1) nm. A model independent Guinier plot for small *q* yields a radius of gyration *R*_G_=(1.56±0.02) nm, which is in accordance with the flexible cylinder dimensions and with the hydrodynamic radius determined by DOSY. The flexible cylinder structure revealed by SANS confirms the above‐mentioned hypothesis of structural disorder such as the formation of rotamers around the biaryl axes blurring the pure J/H‐type spectroscopic appearance.

**Figure 6:**
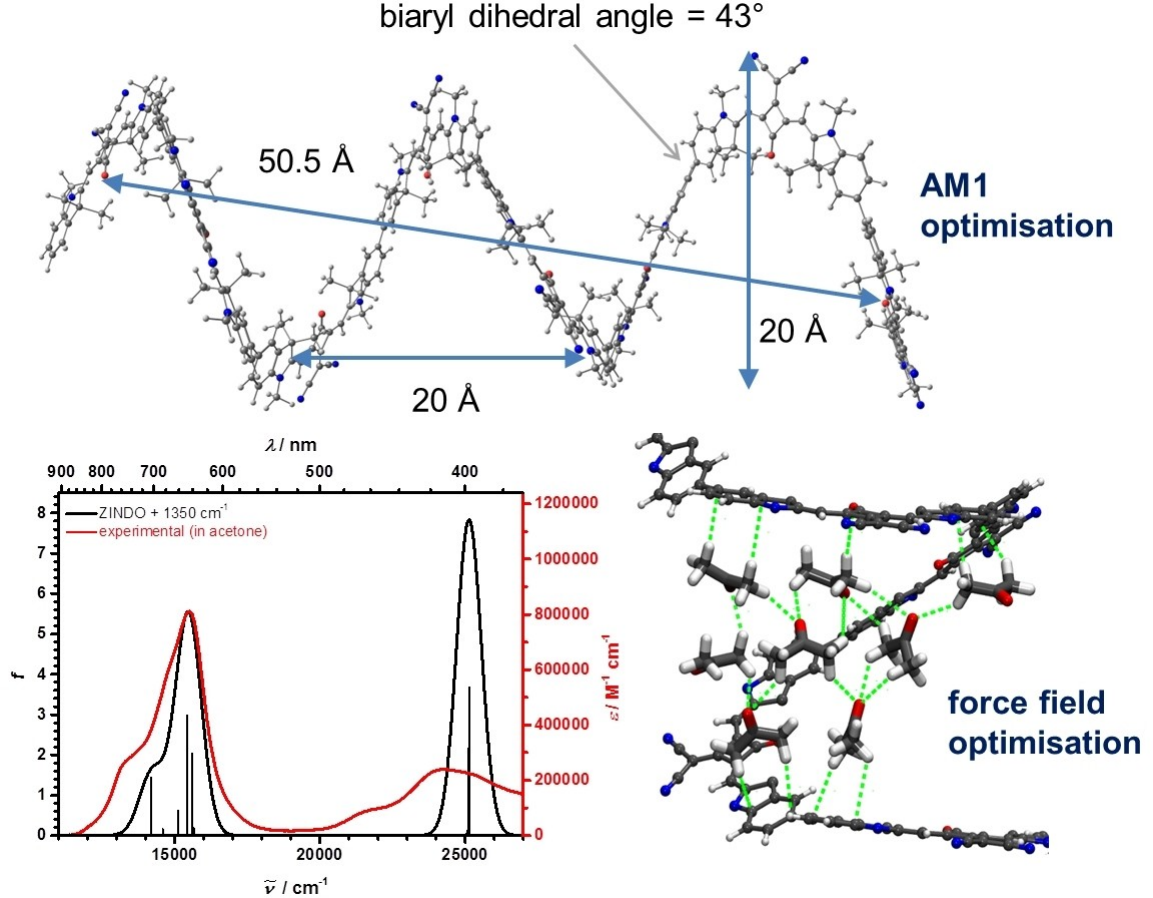
Upper part: AM1 optimized **SQ_8_**. The dimethyloctyl chains are replaced by methyl for simplicity. Lower part left: experimental and ZINDO calculated absorption spectrum on the AM1 optimized structure of **SQ_8_**. The stick spectrum was broadened by Gaussian function with a fwhm of 1000 cm^−1^ and shifted by 1350 cm^−1^. Lower part right: Example for a three layer acetone network connecting two neighbouring helix sheets. The electron rich indole ring systems serve as anchor for the methyl groups of acetone forming a solvation layer around the squaraine. These neighbouring layers are then connected via interlaced acetone molecules. Octyl chains and additional solvent molecules excluded for clarity.

Modelling of the helix structure using the semiempirical AM1 Hamiltonian yields indeed a helix with about 2.7 squaraine dyes per turn which gives an approximate radius of 10 Å (in good agreement with the SANS data) and a separation between the centers of the terminal squaraines of the helix of 50.5 Å (see Figure 6, upper part). The optimized equilibrium biaryl twist angle is about 43°. As the squaraine chromophores are practically stiff and flat units, this biaryl twist angle determines the large helix pitch of about 20 Å. This also is consistent with what appears as Kuhn segment length in the SANS model. Consequently, the helix voids must be filled by solvent molecules. A double helix structure – as seen in e. g., oligo‐*meta*‐phenylenes[^2d^, ^36^] – which would lead to a denser structure and the replacement of the solvent molecules within the helix, though geometrically possible, can safely be excluded as we did not find any concentration dependence of the absorption spectra.^[37]^ Furthermore, the dimethylmethylene groups in the 3‐position of the indolenine heterocycles prevent dense π‐stacking anyway.

We also simulated a solvated helix by propagating the AM1 optimized helical **SQ** octamer including the dimethyl octyl side chains in a periodic acetone solvent box using the CHARMM36 force field. Along the 100 ps trajectory several snapshots were optimized with the forcefield. Inside the helical pitch, the acetone solvent molecules orient with their partially positive charged methyl groups towards the electron rich groups of the squaraine, that is, the indolenines flanking the squaric acid core. This forms the first solvation layer between the helix sheets as seen in Figure 6, lower part right. The simulations show, that the layers between two neighbouring sheets are bridged by another singular layer of acetone. This orientation can be seen throughout multiple simulations and appears to the preferred orientation induced by the helix's extensive π‐system. While obtaining reliable thermodynamic data from QM/MM calculations is unfortunately out of scope due to the size of the system, this recurring structural motif might stabilise the helix and compensate for the entropic contributions at a given temperature. Using that AM1 structural model we also calculated the absorption spectrum of **SQ_8_** by the ZINDO method, see Figure 6 lower part left, which is in very good agreement with the experimental spectrum, particularly in view that vibronic progressions were disregarded in the calculations. This emphasized the correctness of our structural model. The large pitch of our structural model also indicates that the blue shift of absorption maxima (H‐type) is not caused by parallel arrangement of transition dipole moments in π‐stacked squaraines but is because of the *gauche* conformation^[38]^ of the banana‐bent squaraines around the connecting biaryl axis. This can easily be seen when comparing the TD‐DFT computed absorption spectra of linear **SQ_3_** vs. the closed form, see Figure S59 in the Supporting Information.

## Discussion and Conclusion

An iterative cross coupling/deprotection strategy allowed the synthesis of monodisperse **SQ** oligomers and established the chain length dependence of optical spectra, the thermodynamics of helix folding and their geometry. The coil‐helix transition of the longer oligomers follows the cooperative model,^[39]^ that is, as a nucleation step, about three monomers are necessary to form a loop until propagation of the oligomer leads to helix formation. AM1 computations, DOSY and SANS measurements agree on an oligomer helix with an unprecedented large pitch (ca. 20 Å) and, consequently, voids between the squaraine dyes that are filled by solvent molecules. Calculated absorption spectra based on this structural model also are in very good agreement with experiment. The obvious question arises what causes the helix formation if direct π‐π interaction of stacking aromatic systems as found in e. g. oligo(*m*‐phenylene ethynylene)s can be ruled out at this large helix pitch.

In general, in the most favourable case, helix folding requires partial desolvation, that is, removal of solvent molecules which has to be compensated by attractive interactions between helix segments, be it e. g., hydrogen bonding or π‐stacking. Furthermore, because helix folding is despite desolvation usually entropically unfavourable, the entropy term must be overcompensated by the enthalpy term. In our case, folding a helix with a small pitch as to allow π‐stacking interactions would require strong twisting around the biaryl axes between the squaraine dyes beyond the equilibrium angle of ca. 43°. This should afford a significant amount of extra enthalpy which is unfavourable and thus a loose helix is preferred.^[40]^ The solution experiments with diverse solvents shows that the reason for helix folding is a microscopic solute‐solvent interaction which must at least overcompensate the entropy loss of helix formation. The most intriguing observation is that **SQ_9_** displays distinct J‐type behavior in nitrobenzene but H‐type behavior in benzonitrile, although both solvents are very similar in almost all their physical properties (see Table S2 in the Supporting Information). This reminds of a bistable situation in which tiny changes of the solute‐solvent interaction lead to one of the preferred structures and is typical of a cooperative phenomenon. Almost all other aromatic solvents also induce a J‐type behavior as do THF, CHCl_3_ and CH_2_Cl_2_ and thus may be regarded as “good” solvents. DMF, acetone, alcohols, and less polar solvents such as hydrocarbons and dialkyl ethers induce H‐type behavior and are “poor” solvents leading to helix folding. However, titration with hydrogen bonding spermine or octylglucoside^[16]^ did not lead to helix formation of **SQ_9_** in either CHCl_3_, DCM, toluene, or benzonitrile (see Figure S6 and S7 in the Supporting Information). Furthermore, solutions of **SQ_9_** in enantiomerically pure (*S*)‐1‐phenylethylamine or (*S*)‐2‐methylbutanol exhibited J‐type and H‐type spectra, respectively, but no CD signal at all (see Figure S5 in the Supporting Information). Thus, specific hydrogen bonding phenomena appear not to play a decisive role in the helix folding.

None of the usually employed solvent parameters such as dipole moment, refractive index, permittivity, $E_{T}^{N}$ ‐values, or Onsager factors^[41]^ proved to be helpful for explaining the observed selective J‐ or H‐type behaviour. However, the observed trend correlates with the dispersion index of the Hansen solubility parameter which characterises the polarisability of a solvent.^[42]^ Thus, solvents with ***δ***_D_ index larger than 17.4 favor the random coil, while those below, the helix.^[43]^ Here also nitrobenzene (***δ***_D_=20.0) and benzonitrile (***δ***_D_=17.4) in fact differ markedly. The latter is obviously right at the border concerning the polarizability influence on the coil‐helix equilibrium as shown by the above mentioned temperature dependence of absorption spectra. Another aromatic solvent that has a low Hansen dispersion index is hexafluorobenzene (***δ***_D_=16.9) which indeed also induces the H‐type behavior while benzene (***δ***_D_=18.4) with the somewhat larger dispersity index favours the random coil. Therefore, because at the estimated distance of ca. 20 Å, the reason for helix formation cannot be any direct interchromophore interaction, we suppose that the interstitial solvent molecules act as a kind of “glue” which is induced by the static dipole moments of **SQ** monomers (6.9 D).^[44]^ The dipole moment of **SQ** is sizable unlike all other squaraines which are centrosymmetrc with a vanishing dipole moment. According to our solvation experiments, the solvents that qualify for helix formation must be either polar (hydrogen bonding or non‐hydrogen bonding) or nonpolar, but in any case, possess little polarisability, as characterised by small Hansen dispersion indices. Thus, the enthalpy measured above does not represent the helix formation itself but the stabilisation of interstitial solvent by the folded helix. Such an enhanced solvent order was observed e. g., for polymers in hydrocarbon solutions up to a distance of 2 nm,^[45]^ theoretically predicted for several diverse solvents between graphene sheets,^[46]^ on silica surfaces,^[47]^ and for nanoparticles^[48]^ up to a distance of 2 nm. Furthermore, clathrate formation of solvents between alky side chains of aggregated perylene diimide helices was observed to control the helicity of the aggregate.^[49]^ Molecular mechanics simulations of a helix hexamer with freely optimised acetone solvent molecules indeed indicate such a preferred solvent order, see Figure 6. Therefore, it is conceivable that solvent molecules adopt a long‐range order between the turns of the **SQ_n_** helix with a pitch of 2 nm stabilizing the entire solvated helix structure, thus forming a kind of clathrate. Therefore, we conclude that longer squaraine oligomers form helix‐solvent clathrates as an unusual supramolecular entity that has a strong impact on the absorption spectra by exciton coupling interactions. Such a behavior is unprecedented for dye oligomers to the best of our knowledge but should be considered in other supramolecular dye arrangements as well.

## Conflict of interest

The authors declare no conflict of interest.

## Supporting information

As a service to our authors and readers, this journal provides supporting information supplied by the authors. Such materials are peer reviewed and may be re‐organized for online delivery, but are not copy‐edited or typeset. Technical support issues arising from supporting information (other than missing files) should be addressed to the authors.

Supplementary

## References

[1] H.-W. Schmidt , F. Würthner , Angew. Chem. Int. Ed. 2020, 59, 8766–8775; 10.1002/anie.201915643 32020704

[2a] G. Guichard , I. Huc , Chem. Commun. 2011, 47, 5933–5941; 10.1039/c1cc11137j 21483969

[2b] Foldamers: Structure, Properties, and Applications, Wiley-VCH Verlag GmbH & Co. KGaA, Weinheim, 2007;

[2c] E. Yashima , K. Maeda , H. Iida , Y. Furusho , K. Nagai , Chem. Rev. 2009, 109, 6102–6211; 19905011 10.1021/cr900162q

[2d] E. Yashima , N. Ousaka , D. Taura , K. Shimomura , T. Ikai , K. Maeda , Chem. Rev. 2016, 116, 13752–13990; 27754649 10.1021/acs.chemrev.6b00354

[2e] T. Leigh , P. Fernandez-Trillo , Nat. Chem. Rev. 2020, 4, 291–310; 10.1038/s41570-020-0180-5 37127955

[2f] M. Fujiki , Macromol. Rapid Commun. 2001, 22, 539–563;

[2g] H.-J. Kim , W.-C. Zin , M. Lee , J. Am. Chem. Soc. 2004, 126, 7009–7014; 15174870 10.1021/ja049799v

[2h] H.-J. Kim , J.-H. Lee , M. Lee , Angew. Chem. Int. Ed. 2005, 44, 5810–5814; 10.1002/anie.200501270 16130165

[2i] J. W. Wackerly , J. S. Moore , Macromolecules 2006, 39, 7269–7276.

[3] E. Schwartz , M. Koepf , H. J. Kitto , R. J. M. Nolte , A. E. Rowan , Polym. Chem. 2011, 2, 33–47.

[4a] S. Mayer , R. Zentel , Prog. Polym. Sci. 2001, 26, 1973–2013;

[4b] S. Lifson , M. M. Green , C. Andreola , N. C. Peterson , J. Am. Chem. Soc. 1989, 111, 8850–8858;

[4c] M. M. Green , M. P. Reidy , R. D. Johnson , G. Darling , D. J. O′Leary , G. Willson , J. Am. Chem. Soc. 1989, 111, 6452–6454.

[5] T. Ben , Y. Furusho , H. Goto , K. Miwa , E. Yashima , Org. Biomol. Chem. 2009, 7, 2509–2512. 19503921 10.1039/b903546j

[6] D. J. Hill , J. S. Moore , Proc. Natl. Acad. Sci. USA 2002, 99, 5053–5057. 11917139 10.1073/pnas.072642799 PMC122720

[7] D. Seebach , D. F. Hook , A. Glattli , Biopolymers 2006, 84, 23–37. 16235225 10.1002/bip.20391

[8a] Y. Ferrand , I. Huc , Acc. Chem. Res. 2018, 51, 970–977; 29589916 10.1021/acs.accounts.8b00075

[8b] V. Berl , I. Huc , R. G. Khoury , M. J. Krische , J.-M. Lehn , Nature 2000, 407, 720–723. 11048713 10.1038/35037545

[9] D.-W. Zhang , W.-K. Wang , Z.-T. Li , Chem. Rec. 2015, 15, 233–251. 25351894 10.1002/tcr.201402046

[10] D. J. Hill , M. J. Mio , R. B. Prince , T. S. Hughes , J. S. Moore , Chem. Rev. 2001, 101, 3893–4011. 11740924 10.1021/cr990120t

[11] J. C. Nelson , J. G. Saven , J. S. Moore , P. G. Wolynes , Science 1997, 277, 1793–1796. 9295264 10.1126/science.277.5333.1793

[12a] B. M. W. Langeveld-Voss , M. P. T. Christiaans , R. A. J. Janssen , E. W. Meijer , Macromolecules 1998, 31, 6702–6704;

[12b] H. Sugiura , Y. Nigorikawa , Y. Saiki , K. Nakamura , M. Yamaguchi , J. Am. Chem. Soc. 2004, 126, 14858–14864; 15535712 10.1021/ja0478882

[12c] Y. Nagata , T. Yamada , T. Adachi , Y. Akai , T. Yamamoto , M. Suginome , J. Am. Chem. Soc. 2013, 135, 10104–10113; 23773002 10.1021/ja403391m

[12d] M. M. Green , C. Khatri , N. C. Peterson , J. Am. Chem. Soc. 1993, 115, 4941–4942;

[12e] G. Bidan , S. Guillerez , V. Sorokin , Adv. Mater. 1996, 8, 157–160;

[12f] H. Goto , E. Yashima , Y. Okamoto , Chirality 2000, 12, 396–399; 10824159 10.1002/(SICI)1520-636X(2000)12:5/6<396::AID-CHIR17>3.0.CO;2-X

[12g] M. Fujiki , J. R. Koe , M. Motonaga , H. Nakashima , K. Terao , A. Teramoto , J. Am. Chem. Soc. 2001, 123, 6253–6261; 11427048 10.1021/ja0026509

[12h] H. Nakako , R. Nomura , T. Masuda , Macromolecules 2001, 34, 1496–1502;

[12i] K. K. L. Cheuk , J. W. Y. Lam , J. Chen , L. M. Lai , B. Z. Tang , Macromolecules 2003, 36, 5947–5959;

[12j] K. K. L. Cheuk , J. W. Y. Lam , L. M. Lai , Y. Dong , B. Z. Tang , Macromolecules 2003, 36, 9752–9762;

[12k] K. Maeda , K. Morino , E. Yashima , J. Polym. Sci. Part A 2003, 41, 3625–3631;

[12l] K. Morino , K. Maeda , E. Yashima , Macromolecules 2003, 36, 1480–1486;

[12m] K. Maeda , N. Kamiya , E. Yashima , Chem. Eur. J. 2004, 10, 4000–4010; 15317054 10.1002/chem.200400315

[12n] H. Zhao , F. Sanda , T. Masuda , Polymer 2005, 46, 2841–2846;

[12o] K. Yamazaki , A. Yokoyama , T. Yokozawa , Macromolecules 2006, 39, 2432–2434;

[12p] T. Hasegawa , Y. Furusho , H. Katagiri , E. Yashima , Angew. Chem. Int. Ed. 2007, 46, 5885–5888; 10.1002/anie.200701735 17591733

[12q] T. Fukushima , K. Tsuchihara , Macromol. Rapid Commun. 2009, 30, 1334–1338. 21638388 10.1002/marc.200900156

[13a] N. Sakamoto , C. Ikeda , M. Yamamura , T. Nabeshima , Chem. Commun. 2012, 48, 4818–4820; 10.1039/c2cc17513d 22302032

[13b] J. M. Lehn , A. Rigault , J. Siegel , J. Harrowfield , B. Chevrier , D. Moras , Proc. Natl. Acad. Sci. USA 1987, 84, 2565–2569; 3472223 10.1073/pnas.84.9.2565 PMC304698

[13c] T. Sanji , Y. Sato , N. Kato , M. Tanaka , Macromolecules 2007, 40, 4747–4749.

[14a] E. Kolomiets , V. Berl , J.-M. Lehn , Chem. Eur. J. 2007, 13, 5466–5479; 17429821 10.1002/chem.200601826

[14b] K.-C. Sham , C.-C. Yee , Y. Pan , K.-C. Lau , S.-M. Yiu , H.-L. Kwong , RSC Adv. 2014, 4, 14513–14526;

[14c] Y. Okamoto , T. Nakano , E. Ono , K. Hatada , Chem. Lett. 1991, 525–528;

[14d] F. Sanda , K. Terada , T. Masuda , Macromolecules 2005, 38, 8149–8154.

[15a] Y. Yan , B. Qin , Y. Shu , X. Chen , Y. K. Yip , D. Zhang , H. Su , H. Zeng , Org. Lett. 2009, 11, 1201–1204; 19222182 10.1021/ol802679p

[15b] M. Waki , H. Abe , M. Inouye , Chem. Eur. J. 2006, 12, 7839–7847. 16847986 10.1002/chem.200600315

[16] M. Inouye , M. Waki , H. Abe , J. Am. Chem. Soc. 2004, 126, 2022–2027. 14971935 10.1021/ja039371g

[17a] helical polymers where dyes are appended to the main chain are also known: e. g. refs.^[17b,c]^ . There is also a significant number of supramolecular aggregates known where dyes show a helical π-stacking^[17d–g]^ or are linked by other weak forces such as hydrogen or coordination bonding.^[17h,i]^;

[17b] P. A. J. de Witte , M. Castriciano , J. J. L. M. Cornelissen , L. M. Scolaro , R. J. M. Nolte , A. E. Rowan , Chem. Eur. J. 2003, 9, 1775–1781; 12698435 10.1002/chem.200390204

[17c] V. Palermo , M. B. J. Otten , A. Liscio , E. Schwartz , P. A. J. de Witte , M. A. Castriciano , M. M. Wienk , F. Nolde , G. De Luca , J. J. L. M. Cornelissen , R. A. J. Janssen , K. Müllen , A. E. Rowan , R. J. M. Nolte , P. Samori , J. Am. Chem. Soc. 2008, 130, 14605–14614; 18844351 10.1021/ja804069n

[17d] P. A. Korevaar , S. J. George , A. J. Markvoort , M. M. J. Smulders , P. A. J. Hilbers , A. P. H. J. Schenning , T. F. A. De Greef , E. W. Meijer , Nature 2012, 481, 492–496; 22258506 10.1038/nature10720

[17e] A. P. H. J. Schenning , J. van Herrikhuyzen , P. Jonkheijm , Z. Chen , F. Würthner , E. W. Meijer , J. Am. Chem. Soc. 2002, 124, 10252–10253; 12197707 10.1021/ja020378s

[17f] C. Thalacker , F. Würthner , Adv. Funct. Mater. 2002, 12, 209–218;

[17g] F. Würthner , C. R. Saha-Möller , B. Fimmel , S. Ogi , P. Leowanawat , D. Schmidt , Chem. Rev. 2016, 116, 962–1052; 26270260 10.1021/acs.chemrev.5b00188

[17h] M. A. Alam , A. Tsuda , Y. Sei , K. Yamaguchi , T. Aida , Tetrahedron 2008, 64, 8264–8270;

[17i] M. Kumar , S. J. George , Chem. Sci. 2014, 5, 3025–3030.

[18] L. J. Patalag , L. P. Ho , P. G. Jones , D. B. Werz , J. Am. Chem. Soc. 2017, 139, 15104–15113. 28948783 10.1021/jacs.7b08176

[19] C. Ikeda , Z. S. Yoon , M. Park , H. Inoue , D. Kim , A. Osuka , J. Am. Chem. Soc. 2005, 127, 534–535. 15643872 10.1021/ja043428x

[20a] R. S. Stoll , N. Severin , J. P. Rabe , S. Hecht , Adv. Mater. 2006, 18, 1271–1275;

[20b] M. H. Schreck , M. I. S. Röhr , T. Clark , V. Stepanenko , F. Würthner , C. Lambert , Chem. Eur. J. 2019, 25, 2831–2839; 30549333 10.1002/chem.201805685

[20c] U. Mayerhöffer , F. Würthner , Chem. Sci. 2012, 3, 1215–1220;

[20d] S. Das , T. L. Thanulingam , K. G. Thomas , P. V. Kamat , M. V. George , J. Phys. Chem. 1993, 97, 13620–13624;

[20e] H. Chen , K.-Y. Law , J. Perlstein , D. G. Whitten , J. Am. Chem. Soc. 1995, 117, 7257–7258;

[20f] K. T. Arun , B. Epe , D. Ramaiah , J. Phys. Chem. B 2002, 106, 11622–11627;

[20g] G. M. Paterno , L. Moretti , A. J. Barker , C. D′Andrea , A. Luzio , N. Barbero , S. Galliano , C. Barolo , G. Lanzani , F. Scotognella , J. Mater. Chem. C 2017, 5, 7732–7738;

[20h] A. J. McKerrow , E. Buncel , P. M. Kazmaier , Can. J. Chem. 1995, 73, 1605–1615;

[20i] V. Grande , F. Doria , M. Freccero , F. Würthner , Angew. Chem. Int. Ed. 2017, 56, 7520–7524; 10.1002/anie.201702096 28524354

[20j] C.-A. Shen , F. Würthner , Chem. Commun. 2020, 56, 9878–9881; 10.1039/d0cc03686b 32720667

[20k] O. A. Mass , C. K. Wilson , S. K. Roy , M. S. Barclay , L. K. Patten , E. A. Terpetschnig , J. Lee , R. D. Pensack , B. Yurke , W. B. Knowlton , J. Phys. Chem. B 2020, 124, 9636–9647; 33052691 10.1021/acs.jpcb.0c06480 PMC8524781

[20l] C. Martin , D. Bourgault , C. Michel , J. Provost , M. Hervieu , B. Raveau , Eur. J. Solid State Inorg. Chem. 1989, 26, 1–6;

[20m] D. Zhang , Y.-X. Zhao , Z.-Y. Qiao , U. Mayerhöffer , P. Spenst , X.-J. Li , F. Würthner , H. Wang , Bioconjugate Chem. 2014, 25, 2021–2029; 10.1021/bc5003983 25370305

[20n] A. Kaczmarek-Kedziera , D. Kedziera , Theor. Chem. Acc. 2016, 135, 1–17;

[20o] A. Kaczmarek-Kedziera , P. S. Zuchowski , D. Kedziera , Sci. Rep. 2020, 10, 19670. 33184323 10.1038/s41598-020-76631-z PMC7665223

[21a] S. F. Völker , T. Dellermann , H. Ceymann , M. Holzapfel , C. Lambert , J. Polym. Sci. Part A 2014, 52, 890–911;

[21b] S. F. Völker , A. Schmiedel , M. Holzapfel , C. Böhm , C. Lambert , Phys. Chem. Chem. Phys. 2013, 15, 19831–19844; 24145596 10.1039/c3cp53455c

[21c] S. F. Völker , C. Lambert , Chem. Mater. 2012, 24, 2541–2553;

[21d] S. F. Völker , A. Schmiedel , M. Holzapfel , K. Renziehausen , V. Engel , C. Lambert , J. Phys. Chem. C 2014, 118, 17467–17482.

[22a] T. Maeda , S. Arikawa , H. Nakao , S. Yagi , H. Nakazumi , New J. Chem. 2013, 37, 701–708;

[22b] U. Mayerhöffer , B. Fimmel , F. Würthner , Angew. Chem. Int. Ed. 2012, 51, 164–167; 10.1002/anie.201107176 22105993

[22c] K. Ilina , W. M. MacCuaig , M. Laramie , J. N. Jeouty , L. R. McNally , M. Henary , Bioconjugate Chem. 2020, 31, 194–213; 10.1021/acs.bioconjchem.9b00482 PMC7845514 31365819

[22d] B. Oswald , M. Gruber , M. Bohmer , F. Lehmann , M. Probst , O. S. Wolfbeis , Photochem. Photobiol. 2001, 74, 237–245. 11547561 10.1562/0031-8655(2001)074<0237:ndlcfa>2.0.co;2

[23] A. L. Tatarets , I. A. Fedyunyaeva , E. Terpetschnig , L. D. Patsenker , Dyes Pigm. 2004, 64, 125–134.

[24] E. G. McRae, M. Kasha, Physical Processes in Radiation Biology, Augenstein L.G., Rosenberg B., Mason R. (Eds.), Academic Press, New York, **1964**, pp. 23–42.

[25] C. Brüning , E. Welz , A. Heilos , V. Stehr , C. Walter , B. Engels , S. F. Völker , C. Lambert , V. Engel , J. Phys. Chem. C 2015, 119, 6174–6180.

[26] C. Lambert , F. Koch , S. F. Völker , A. Schmiedel , M. Holzapfel , A. Humeniuk , M. I. S. Röhr , R. Mitric , T. Brixner , J. Am. Chem. Soc. 2015, 137, 7851–7861. 26016517 10.1021/jacs.5b03644

[27] M. T. Stone , J. M. Heemstra , J. S. Moore , Acc. Chem. Res. 2006, 39, 11–20. 16411735 10.1021/ar0501267

[28a] H. Noguchi , K. Hojo , M. Suginome , J. Am. Chem. Soc. 2007, 129, 758–759; 17243801 10.1021/ja067975p

[28b] N. Iwadate , M. Suginome , J. Am. Chem. Soc. 2010, 132, 2548–2549; 20141128 10.1021/ja1000642

[28c] H. Noguchi , T. Shioda , C.-M. Chou , M. Suginome , Org. Lett. 2008, 10, 377–380. 18183990 10.1021/ol702420x

[29a] E. J. Wren , X. Wang , A. Farlow , S.-C. Lo , P. L. Burn , P. Meredith , Org. Lett. 2010, 12, 4338–4340; 20825164 10.1021/ol101717c

[29b] A. R. Chianese , A. Mo , D. Datta , Organometallics 2009, 28, 465–472.

[30] H. Ceymann , A. Rosspeintner , M. H. Schreck , C. Mützel , A. Stoy , E. Vauthey , C. Lambert , Phys. Chem. Chem. Phys. 2016, 18, 16404–16413. 27264847 10.1039/c6cp02312f

[31] boronic acids are prone to condensation reactions, e. g. formation of boroxine derivatives, making NMR spectroscopical and mass spectrometrical characterisations difficult.

[32] E. Michail , M. H. Schreck , M. Holzapfel , C. Lambert , Phys. Chem. Chem. Phys. 2020, 22, 18340–18350. 32785389 10.1039/d0cp03410j

[33] in some very polar solvents **SQB_9_** proved to be insoluble: formamide, DMSO, nitromethane, ethanol.

[34] R. B. Prince , J. G. Saven , P. G. Wolynes , J. S. Moore , J. Am. Chem. Soc. 1999, 121, 3114–3121.

[35a] A. A. Hernandez Santiago , A. S. Buchelnikov , M. A. Rubinson , S. O. Yesylevskyy , J. A. Parkinson , M. P. Evstigneev , J. Chem. Phys. 2015, 142, 104202; 25770533 10.1063/1.4913974

[35b] L. Allouche , A. Marquis , J.-M. Lehn , Chem. Eur. J. 2006, 12, 7520–7525. 16874831 10.1002/chem.200600552

[36] H. Goto , H. Katagiri , Y. Furusho , E. Yashima , J. Am. Chem. Soc. 2006, 128, 7176–7178. 16734467 10.1021/ja062171v

[37] assuming a hypothetical dimerization equilibrium we fitted the temperature dependent absorption spectra in PhCN and extracted the associated equilibrium constant at r.t. Using this equilibrium constant we simulated the expected concentration dependence which turns out to be significant and which is in disagreement with the observation of concentration-independent spectra (see the SI) and thus the formation of a double helix.

[38] C. Zhong , D. Bialas , C. J. Collison , F. C. Spano , J. Phys. Chem. C 2019, 123, 18734–18745.

[39] H. Qian , J. A. Schellman , J. Phys. Chem. 1992, 96, 3987–3994.

[40] twisting the biaryl axis in the octamer from the AM1 optimised equilibrium structure with an angle of ca. 43° to 20° or even to 10° rises the energy by 6.4 or 13.4 kJ mol^−1^ per dihedral angle and reduces the pitch to ca. 12.6 or 8.9 Å in the helix. Thus, even further twisting would be necessary in order to allow for π-stacking between the helix turns.

[41] C. Reichardt , T. Welton , Solvents and solvent effects in organic chemistry, fourth ed., Wiley-VCH Verlag GmbH & Co. KGaA, Weinheim, 2011.

[42] C. Hansen , Hansen Solubility Parameters: A User's Handbook, Second Edition, CDC Press, Boca Raton, 2007.

[43] the only exception is THF but in that case the low polarity parameter compensates for that.

[44] DFT calculation of **SQB** at B3LYP/6-31G*.

[45] G. W. Longman , G. D. Wignall , R. P. Sheldon , Polymer 1979, 20, 1063–1070.

[46] F. Ramirez Rozzi , Sci. Rep. 2016, 6, 27405. 27305976 10.1038/srep27405 PMC4910065

[47] X. Zhang , M. M. Cunningham , R. A. Walker , J. Phys. Chem. B 2003, 107, 3183–3195.

[48] M. Zobel , R. B. Neder , S. A. J. Kimber , Science 2015, 347, 292–294. 25593188 10.1126/science.1261412

[49] C. Kulkarni , P. A. Korevaar , K. K. Bejagam , A. R. A. Palmans , E. W. Meijer , S. J. George , J. Am. Chem. Soc. 2017, 139, 13867–13875. 28891291 10.1021/jacs.7b07639

