# Supplementary material for: Solvent Induced Helix Folding of Defined Indolenine Squaraine Oligomers
Source: Chemistry. 2021 May 7;27(32):8380–9. doi: 10.1002/chem.202101063 (PMC8251825; doi:10.1002/chem.202101063)
Supplement: Supplementary file 1 — Supplementary [file CHEM-27-8380-s001.pdf]

Chemistry–A European Journal

Supporting Information

Solvent Induced Helix Folding of Defined Indolenine Squaraine Oligomers

Arthur Turkin, Marco Holzapfel, Mohit Agarwal, David Fischermeier, Roland Mitric, Ralf Schweins, Franziska Gröhn, and Christoph Lambert*

Table of Contents

1 Materials and Methods 1
2 Synthesis 4
2.1 Synthetic Route of Odd-Numbered Oligomers 11
2.2 Synthetic Route of Even-Numbered Oligomers 26
3 Absorption Spectroscopic Data of Squaraine Oligomers 39
3.1 Temperature Dependent Absorption Spectroscopy 46
3.2 Absorption Spectroscopy in Solvent Mixtures 49
4 NMR Spectroscopy 51
5 Small Angle Neutron Scattering (SANS) 75
6 Calculations 77
7 References 82

1 Materials and Methods

NMR Spectroscopy

- Avance III HD 400 FT-Spectrometer (^{1}H: 400.13 MHz, ^{13}C: 100.61 MHz) with a Bruker Ultrashield magnet
- Avance III HD 400 FT-Spectrometer (^{1}H: 400.03 MHz, ^{13}C: 100.59 MHz) with a Bruker Ascend magnet
- Avance III HD 600 FT-Spectrometer (^{1}H: 600.13 MHz, ^{13}C: 150.90 MHz) with an Oxford Instruments magnet
- Avance III HD 600 FT-Spectrometer (^{1}H: 600.43 MHz, ^{13}C: 150.98 MHz) with a Bruker Ascend magnet

^{1}H and ^{13}C spectra were acquired using one of the spectrometer listed above with sample concentrations of > 0.1 mM in deuterated solvents (acetone-*d*$_6$, CD_2Cl_2, $CDCl_3$, dimethylsulfoxide-*d*$_6$). All solvents were used as received. Chemical shifts are given in ppm relative to the residual nondeuterated solvent signal (^{1}H; CH_2Cl_2: δ 5.32 $CHCl_3$: *δ* 7.26, acetone: *δ* 2.05, dimethylsulfoxide: *δ* 2.50; ^{13}C: CH_2Cl_2: *δ* 53.84, $CHCl_3$: *δ* 77.16, acetone: *δ* 29.87, dimethylsulfoxide: *δ* 39.52).[1] The abbreviations used for assigning the spin multiplicities and C-atoms are: s = singlet, d = doublet, t = triplet, m = multiplet, dd = doublet, ddd = doublet of doublet of doublet, td = triplet of doublet; prim = primary, sec = secondary, tert = tertiary, quart = quarternary. Multiplet signals or overlapping multiplet signals in ^{1}H NMR spectra that could not

be assigned to first order couplings are given as (-). The coupling constants are given in Hertz (Hz). Order of description for ^{1}H NMR spectra: chemical shift (spin multiplicity, coupling constant, number of protons, assignment). The recorded spectra are displayed in Figure S12-S44.

DOSY NMR measurements were performed in acetone-d_6 and CD_2Cl_2. The hydrodynamic radii R_0 of the oligomers were calculated from the corresponding diffusion coefficients D of the Stokes-Einstein equation (S1) at 293 K:

$$D = \frac{k_B T}{6\pi\eta R_0} \qquad \text{S1}$$

where k_B is the Boltzmann constant, T is the temperatue, and η is the viscosity of the solvent (η_{DCM} = 4.40 × 10^{-4} Ns m^{-2} and $\eta_{acetone}$ = 3.20 × 10^{-4} Ns m^{-2}). The sample concentration of each measurement was approx. 1.29 × 10^{-3} M and thus not adjusted to the number of repeating units. For the evaluation of the data the TopSpin Software (v.3.2) from Bruker GmbH was used. The squaraine peaks were integrated and the exported data fitted with the function type "vargard". The resulting diffusion constants were averaged and further viand used in the Stokes-Einstein equation in order to calculate the radius R_0. The recorded spectra are displayed in Figure S48 – S56.

VT (variable temperature) NMR measurements were performed in benzonitrile-d_5 (PhCN-d_5) in an NMR tube equipped with a valve for moderate pressure from Wilmad-LabGlass (Vineland, USA). The temperature ranges from 268 K – 423 K in 10 K steps. The chemical shift of PhCN-d_5 was referenced via tetrakis(trimethylsilyl)silane relative to the residual nondeuterated solvent signal (δ = 7.23 ppm for the right peak). Dry nitrogen gas was used to prevent water condensation at the NMR tube.

Mass Spectrometry

- Bruker Daltonics microTOF focus (ESI)
- Bruker Daltonics autoflex II LRF (MALDI)
- Bruker Daltonics UltrafleXtreme (MALDI)

Mass spectra were recorded with a Bruker Daltonics autoflex II LRF or a Bruker Daltonics UltrafleXtreme (MALDI) in positive mode (POS) using a DCTB (*trans*-2-[3-(4-*tert*-butylphenyl)-2-methyl-2-propenylidene]malononitrile) matrix. High resolution mass spectra were recorded with a Bruker Daltonic microTOF focus (ESI). All mass spectra peaks are reported as m/z. For calculation of the respective mass values of the isotopic distributions the software Compass 1.1 from Bruker Daltonics GmbH (Bremen, Germany) was used. Calculated (calc.) and measured

(found) peak values refer to the most intense peak of the isotopic distributions or the monoisotopic mass.

Recycling Gel Permeation Chromatography (GPC)

Shimadzu Recycling GPC-System

- Photodiode array UV/vis detector (SPD-M20A, 190-800 nm)
- Card type system controller (CBM-20Alite)
- Liquid Chromatograph (LC-20AD)
- Degassing unit (DGU-20A3R)
- Shimadzu valve unit (FCV-20AH$_2$)
- Fraction collector (FRC-10A)
- Shimadzu LCsolution (v. 1.25
- Preparative PSS columns (styrene-divinylbenzene-copolymer / 10µ / 20 × 600 mm / 50 Ǻ,100 Ǻ, 500 Ǻ)

Gel permeation chromatography (GPC) was done using two columns from PSS. $CHCl_3$ was used as eluent with a flow rate of 4-6 mL min^{-1}.

Recycling High Pressure Liquid Chromatography (HPLC)

Jasco Recycling GPC/HPLC system

- Four Channel UV/vis detector (195-700 nm)
- Fraction collector (CHF 122SC)
- (Semi-)preparative MN HPLC columns (VP Nucleosil / 5µ / 21 × 250 mm / 120 Ǻ)

High Pressure Liquid Chromatography (HPLC) was done using two columns from Macherey-Nagel. DCM + 0.2 % MeOH was used as eluent with a flow rate of 10 mL min^{-1}.

Solvent Purification System (SPS)

Inert anhydrous solvent dispensing system

- Model: PS-MD-6/7-EN
- Drying Columns: dual purifying columns design / polystyrene / medium density / 101 × 635 mm / 4 L internal volume
- 29/30 stainless steel glassware adaptors
- Particle filter (7 micron)
- 17 L solvent reservoirs for (DCM, DMF, Et_2O, toluene, THF)

HPLC grade and stabilizer free solvents were used to fill the reservoirs. Solvent was taken from the outlet valves, degassed for 15 min and stored in flame dried Schlenk flasks.

2 Synthesis

All reactions were carried out in standard glass ware. All chemicals were purchased from commercial suppliers and were used without further purification. For reactions performed under nitrogen atmosphere flame-dried Schlenk flasks were used. Nitrogen was dried over Sicapent® from Merck and oxygen was removed via copper catalyst R3-11 from BASF. Solvents, if not taken from the SPS, were dried according to standard literature procedure and stored under inert gas atmosphere. Column chromatography and thin layer chromatography were performed on silica gel (40 – 63 µm, Macherey-Nagel) in wet-packed glass columns and TLC plates (Macherey-Nagel), respectively.

Reagents

Synthesis of 2,3,3-trimethyl-3*H*-indole (**1**), 5-bromo-1-(3,7-dimethyloctyl)-2,3,3-trimethyl-3*H*-indol-1-ium iodide, (E)-3-((5-bromo-1-(3,7-dimethyloctyl)-3,3-dimethylindolin-2-ylidene)methyl)-4-ethoxycyclobut-3-ene-1,2-dione, (E)-2-((5-bromo-1-(3,7-dimethyloctyl)-3,3-dimethylindolin-2-ylidene)methyl)-3-(dicyanomethylene)-4-oxocyclobut-1-en-1-olate (**4**), 2-(4,4,5,5-tetramethyl-1,3,2-dioxaborolan-2-yl)-2,3-dihydro-1*H*-naphtho[1,8-de][1,3,2]diazaborinine (**(pin)B-B(dan)**), **SQ**, **SQ-Br** and **SQ-Bpin$_2$** according to literature.[2]

SQ$_2$ was thankfully provided by Dr. M. Schreck

General Procedure for the Deprotection of 1,8-Diaminonaphthyl Protected Boron Compounds (GP I)

Under a nitrogen atmosphere, in an oven dried flask a protected squaraine (1.00 eq) was dissolved in air- and peroxide free THF and 2 M hydrochloric acid (24.0 eq) was added. The solution was stirred for 24 h at rt. DCM was added and the residue was filtered off. The intermediate product was extracted using DCM, dried over $MgSO_4$ and the solvent was removed *in vacuo*. The product was used without further purification.

General Procedure for the Iterative Cross-Coupling (GP II)

Under a nitrogen atmosphere, in an oven dried flask **SQ-BrBdan**, or **SQ-Bpin$_2$**, or **SQ$_2$-Bdan$_2$**, or an intermediate product, a building block (**SQ-BrBdan**, **SQ-BpinBdan** or **SQ-Br**, 2.40 eq), XPhos-Pd-G2 catalyst (0.10 eq), XPhos (0.20 eq) and Cs_2CO_3 (4.00 eq) were dissolved in a degassed THF/water (4:1) mixture. The reaction mixture was stirred at 60 °C for 24 h. The mixture was diluted with water, extracted using DCM and dried over $MgSO_4$. After removing the solvent *in vacuo* the crude product was purified by flash column chromatography, precipitation in *n*-hexane and if necessary by preparative recycling GPC or HPLC.

Synthetic Route of the Squaraine Building Blocks

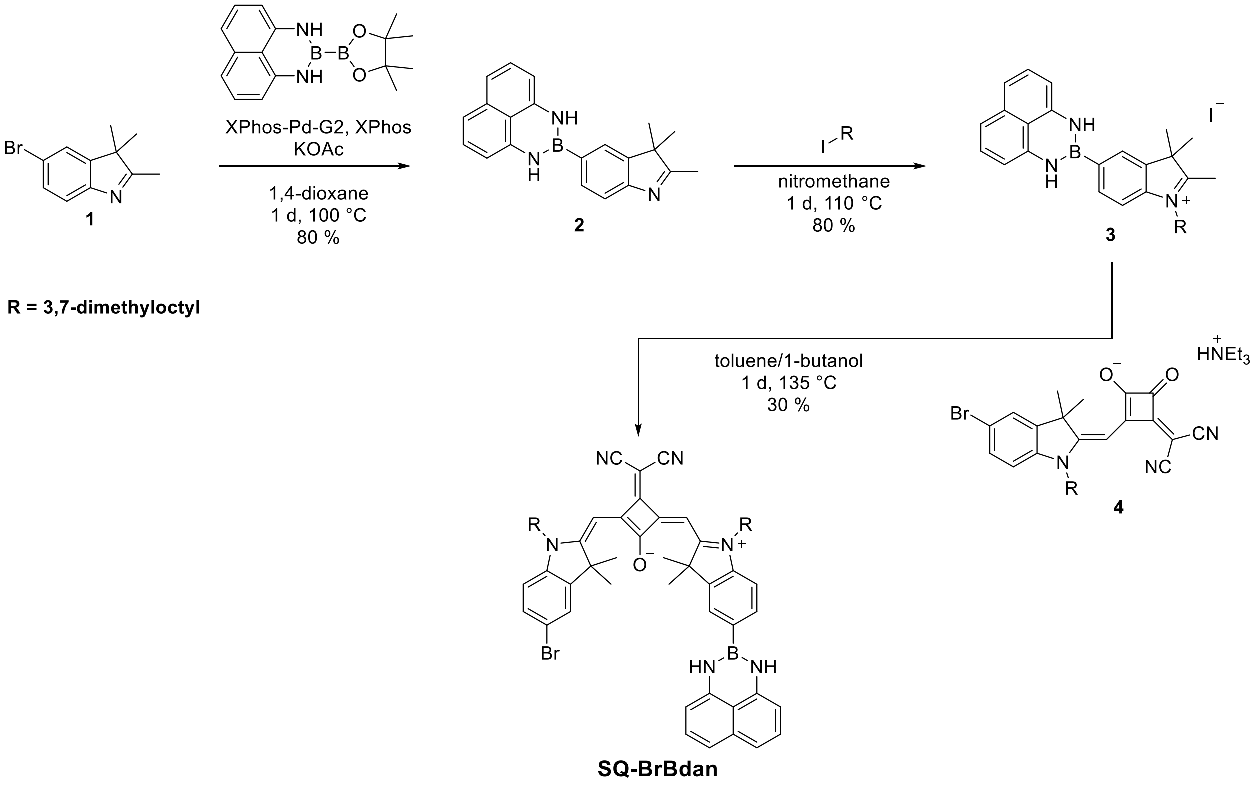

Scheme S1. Synthetic route of the building block **SQ-BrBdan**.

Synthesis of **2-(2,3,3-Trimethyl-3*H*-indol-5-yl)-2,3-dihydro-1*H*-naphtho[1,8-de][1,3,2]diazaborinine (2)**

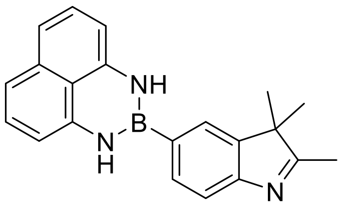

Synthesis following literature.[3]

Under a nitrogen atmosphere 5-bromo-2,3,3-trimethyl-3*H*-indole (3.59 g, 15.1 mmol), (pin)B-B(dan) (4.43 g, 15.1 mmol), potassium acetate (4.44 g, 45.2 mmol), XPhos-Pd-G2 catalyst (356 mg, 452 µmol) and XPhos (431 µg, 905 µmol) were dissolved in dry 1,4-dioxane (50 mL), previously degassed for 15 min. After stirring for 24 h at 100 °C the crude mixture was filtrated and the solvent removed *in vacuo*. The residue was purified by flash column chromatography (eluent: PE/EA 2:1 → 1:1).
Yield: 3.93 g (12.1 mmol, 80 %) of a brown solid.

$C_{21}H_{20}BN_3$ [325.22]

^{1}H NMR (400.1 MHz, $CDCl_3$, 295 K):

δ [ppm] = 7.65 – 7.60 (-, 2H, 2 × -CH-), 7.59 – 7.56 (m, 1H, -CH-), 7.16 (d, $^3J_{HH}$ = 7.3 Hz, 1H, -CH-), 7.14 (d, $^3J_{HH}$ = 7.3 Hz, 1H, -CH-), 7.06 (dd, $^3J_{HH}$ = 8.4 Hz, $^5J_{HH}$ = 0.9 Hz, 2H, 2 × -CH-), 6.44 (dd, $^3J_{HH}$ = 7.3 Hz, $^5J_{HH}$ = 1.0 Hz, 2H, 2 × -CH-), 6.06 (bs, 2H, 2 × -NH-), 2.36 (s, 3H, CH_3), 1.38 (s, 6H, -C(CH_3)$_2$).

^{13}C NMR (100.6 MHz, $CDCl_3$, 295 K):

δ [ppm] = 189.9 (quart.), 153.7 (quart.), 145.4 (quart), 141.2 (2 × quart.), 136.5 (quart.), 131.5 (tert.), 127.8 (2 × tert.), 124.4 (tert.), 119.9 (quart.), 119.8 (tert.), 118.0 (2 × tert.), 106.2 (2 × tert.), 53.8 (quart.), 23.3 (2 × prim.), 15.6 (prim.).

The boron-bound carbon was not found due to quadrupolar relaxation.

MALDI-MS pos (high res.): [$M^{•+}$]

calc.: 325.17503

found: 325.17297

Synthesis of **1-(3,7-dimethyloctyl)-2,3,3-trimethyl-5-(1*H*-naphtho[1,8-de][1,3,2]diazaborinin-2(3*H*)-yl)-3*H*-indol-1-ium iodide (3)**

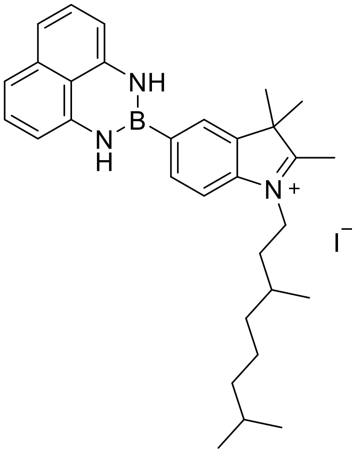

Synthesis according to literature.[2a]

2-(2,3,3-trimethyl-3*H*-indol-5-yl)-2,3-dihydro-1*H*-naphtho[1,8-de][1,3,2]diazaborinine (**2**) (4.98 g, 15.3 mmol) and 1-iodo-3,7-dimethyloctane (9.03 g, 33.7 mmol) were dissolved in nitromethane (60 mL) and stirred at 110 °C for 1 d. After cooling to rt, the solvent was removed *in vacuo* and Et_2O (50 mL) was added to the residue and the mixture was left at −30 °C for 3 h. The precipitate was filtered off, washed with Et_2O (200 mL) and dried under high vacuum.
Yield: 7.80 g (13.2 mmol, 86 %) of a brown solid.

$C_{31}H_{41}BN_2N^+I^-$ [593.40]

^{1}H NMR (400.1 MHz, DMSO-d_6, 295 K):

δ [ppm] = 8.40 (s, 2H, 2 × -N$\underline{H}$), 8.33 (s, 1H, -C$\underline{H}$-), 8.17 (d, $^3J_{HH}$ = 8.0 Hz, 1H, -C$\underline{H}$-), 7.98 (d, $^3J_{HH}$ = 8.2 Hz, 1H, -C$\underline{H}$-), 7.12 (t, $^3J_{HH}$ = 7.8 Hz, 2H, 2 × -C$\underline{H}$-), 6.95 (d, $^3J_{HH}$ = 7.7 Hz, 2H, 2 × -C$\underline{H}$-), 6.60 (d, $^3J_{HH}$ = 7.4 Hz, $^4J_{HH}$ = 0.7 Hz, 2H, 2 × -C$\underline{H}$-), 4.48 (t, $^3J_{HH}$ = 7.5 Hz, 2H, -NC$\underline{H_2}$-), 2.87 (s, 3H, -CC$\underline{H_3}$), 1.93 – 1.79 (m, 1H, -NCH$_2$C$\underline{H_2}$-), 1.73 – 1.56 (-, 2H, -NCH$_2$C$\underline{H_2}$-, -C$\underline{H}$CH$_3$), 1.59 (s, 6H, -C(C$\underline{H_3}$)$_2$), 1.56 – 1.44 (m, 1H, -C$\underline{H}$(CH$_3$)$_2$), 1.43 – 1.29 (m, 2H, -C$\underline{H_2}$-), 1-29 – 1.19 (m, 2H, -C$\underline{H_2}$-), 1.19 – 1.07 (m, 2H, -C$\underline{H_2}$-), 1.02 (d, $^3J_{HH}$ = 6.1 Hz, 3H, -CHC$\underline{H_3}$), 0.851 (d, $^3J_{HH}$ = 6.6 Hz, 3H, -CH(C$\underline{H_3}$)$_2$), 0.849 (d, $^3J_{HH}$ = 6.6 Hz, 3H, -CH(C$\underline{H_3}$)$_2$).

^{13}C NMR (100.6 MHz, DMSO-d_6, 295 K):

δ [ppm] = 196.8 (quart.), 149.8 (quart.), 142.3 (quart.), 142.1 (2 × quart.), 136.0 (quart.), 133.3 (tert.), 127.8 (2 × tert.), 127.4 (tert.), 119.8 (quart.), 116.7 (2 × tert.), 114.5

(tert.), 105.8 (2 × tert.), 55.0 (quart.), 46.2 (sec.), 38.7 (sec.), 36.3 (sec.), 34.0 (sec.), 30.3 (tert.), 27.4 (tert.), 24.0 (sec.), 22.6 (prim.), 22.5 (prim.), 22.31 (prim.), 22.27 (prim.), 19.2 (prim.), 14.0 (prim.).

The boron-bound carbon was not found due to quadrupolar relaxation.

MALDI-MS pos (high res.): [$M^{\bullet+}$-I^-]

calc.: 466.33881

found: 466.33845

Synthesis of **SQ-BrBdan**

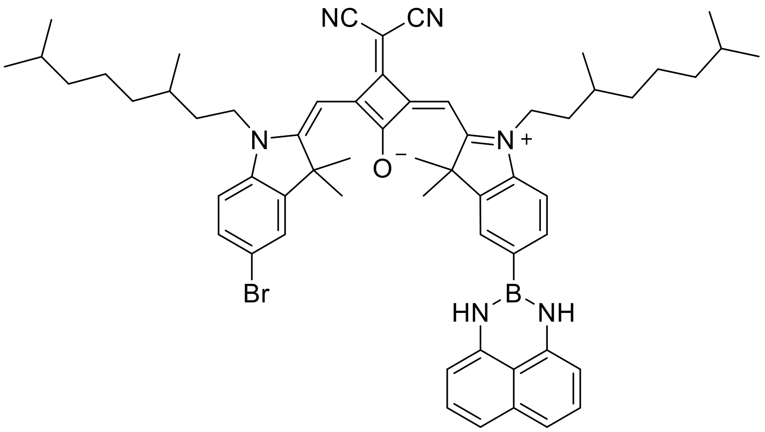

Synthesis following literature.[4]

3 (1.52 g, 2.56 mmol) and **4** (1.68 g, 2.69 mmol) were dissolved in a mixture of 1-butanol/toluene 1:1 (120 mL). Pyridine (20 mL) was added and the solution stirred at 135 °C for 18 h. The solvent was removed *in vacuo* and the crude product purified by flash column chromatography (eluent: DCM). Finally, the product was further purified via precipitation in *n*-hexane. The precipitate was filtered off and dried under high vacuum.
Yield: 781 mg (805 µmol, 32 %) of a green solid.

$C_{59}H_{70}BBrN_6O$ [969.96]

^{1}H NMR (400.1 MHz, CD_2Cl_2, 295 K):

δ [ppm] = 7.72 – 7.65 (-, 2H, 2 × -CH-), 7.51 (d, $^4J_{HH}$ = 1.8 Hz, 1H, -CH-), 7.48 (dd, $^3J_{HH}$ = 8.4 Hz, $^4J_{HH}$ = 2.0 Hz, 1H, -CH-), 7.18 (d, $^4J_{HH}$ = 2.6 Hz, 1H, -CH-), 7.16 (d, $^4J_{HH}$ = 3.6 Hz, 1H, -CH-), 7.15 (d, $^3J_{HH}$ = 7.3 Hz, 1H, -CH-), 7.06 (dd, $^3J_{HH}$ = 8.4 Hz, $^5J_{HH}$ = 0.9 Hz, 2H, 2 × -CH-), 6.93 (d, $^3J_{HH}$ = 8.4 Hz, 1H, -CH-), 6.53 (s, 1H, -CCHC-), 6.50 (dd, $^3J_{HH}$ = 7.3 Hz, $^4J_{HH}$ = 0.9 Hz, 2H, 2 × -CH-), 6.47 (s,

1H, -CCHC-), 6.20 (bs, 2H, 2 × -NH-), 4.18 – 3.89 (-, 4H, 2 × -NCH$_2$-), 1.89 – 1.71 (-, 2H, 2 × -NCH$_2$CH$_2$-), 1.80 (s, 6H, -C(CH$_3$)$_2$), 1.76 (s, 6H, -C(CH$_3$)$_2$), 1.70 – 1.11 (-, 18H, 2 × -NCH$_2$CH$_2$-, 2 × -CHCH$_3$, 2 × -CH(CH$_3$)$_2$, 2 × -CH$_2$CH$_2$CH$_2$-), 1.04 (d, $^3J_{HH}$ = 6.4 Hz, 3H, -CHCH$_3$), 1.02 (d, $^3J_{HH}$ = 6.4 Hz, 3H, -CHCH$_3$), 0.87 (d, $^3J_{HH}$ = 6.6 Hz, 6H, -CH(CH$_3$)$_2$), 0.86 (d, $^3J_{HH}$ = 6.6 Hz, 6H, -CH(CH$_3$)$_2$).

^{13}C NMR (100.6 MHz, CD$_2$Cl$_2$, 295 K):

δ [ppm] = 173.3 (quart.), 172.4 (quart.), 171.3 (quart.), 168.1 (quart.), 167.5 (quart.), 166.9 (quart.), 144.9 (quart.), 144.1 (quart.), 142.8 (quart.), 141.53 (quart.), 141.46 (2 × quart.), 136.7 (quart.), 132.1 (tert.), 131.3 (tert.), 128.1 (2 × tert.), 126.1 (tert.), 125.6 (tert.), 120.1 (quart.), 119.0 (2 × quart.), 118.1 (2 × tert.), 117.6 (quart.), 111.8 (tert.), 110.5 (tert.), 106.4 (2 × tert.), 90.0 (tert.), 89.7 (tert.), 49.9 (quart.), 49.8 (quart.), 43.46 (sec.), 43.41 (sec.), 41.0 (quart.) 39.53 (sec.), 39.51 (sec.), 37.48 (sec.), 37.46 (sec.), 34.44 (sec.), 34.26 (sec.), 31.32 (tert.), 31.28 (tert.), 28.394 (tert.), 28.389 (tert.), 26.80 (prim.), 26.75 (prim.), 26.74 (prim.), 26.70 (prim.), 25.0 (2 × sec.), 22.83 (prim.), 22.82 (prim.), 22.74 (prim), 22.73 (prim), 19.80 (prim.), 19.77 (prim.).

The boron-bound carbon was not found due to quadrupolar relaxation.

MALDI-MS pos (high res.): [M$^{\bullet+}$]

calc.: 968.48875

found: 968.49171

Synthesis of **SQ-BpinBdan**

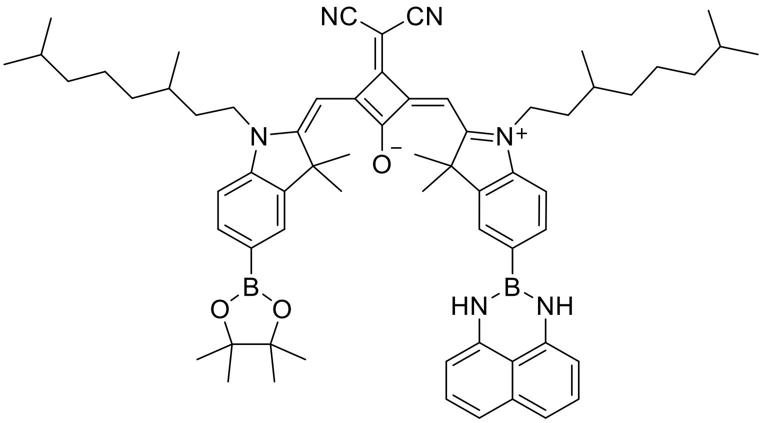

Synthesis following literature.[2a]

Under a nitrogen atmosphere, $PdCl_2(PhCN)_2$ catalyst (9.89 mg, 26.0 µmol), dppf (14.0 mg, 26.0 µmol) in 1,4-dioxane (10 mL) heated at 60 °C for 30 min; catalyst mixture was added to **SQ-BrBdan** (500 mg, 515 µmol), B_2pin_2 (183 mg, 722 µmol), potassium acetat (162 mg, 1.65 mmol) in 1,4-dioxane (40 mL) and heated at 115 °C for 1 d; flash column chromatography (eluent: DCM + 0.1 → 0.5 % MeOH), precipitation in *n*-hexane.
Yield: 179 mg (176 µmol, 34 %) of a green solid.

$C_{65}H_{82}B_2N_6O_3$ [1017.03]

^{1}H NMR (400.1 MHz, CD_2Cl_2, 295 K):

δ [ppm] = 7.79 (dd, $^3J_{HH}$ = 7.9 Hz, $^4J_{HH}$ = 1.1 Hz, 1H, -C$\underline{H}$-), 7.77 – 7.76 (m, 1H, -C$\underline{H}$-), 7.68 (dd, $^3J_{HH}$ = 8.1 Hz, $^4J_{HH}$ = 1.2 Hz, 1H, -C$\underline{H}$-), 7.68 (d, $^4J_{HH}$ = 1.4 Hz, 1H, -C$\underline{H}$-), 7.19 – 7.13 (-, 3H, 3 × -C$\underline{H}$-), 7.09 – 7.04 (-, 3H, 3 × -C$\underline{H}$-), 6.52 (s, 1H, -CC$\underline{H}$C-), 6.51 (s, 1H, -CC$\underline{H}$C-), 6.50 (dd, $^3J_{HH}$ = 7.4 Hz, $^4J_{HH}$ = 1.0 Hz, 2H, 2 × -C$\underline{H}$-), 6.21 (s, 2H, 2 × -N$\underline{H}$), 4.28 – 3.96 (-, 4H, 2 × -NC$\underline{H_2}$-), 1.87 – 1.72 (-, 2H, 2 × -NCH$_2$C$\underline{H_2}$-), 1.81 (s, 6H, -C(C$\underline{H_3}$)$_2$), 1.77 (s, 6H, -C(C$\underline{H_3}$)$_2$), 1.72 – 1.58 (-, 4H, 2 × -NCH$_2$C$\underline{H_2}$-, 2 × -C$\underline{H}$CH$_3$), 1.58 – 1.46(-, 2H, 2 × -C$\underline{H}$(CH$_3$)$_2$), 1.46 – 1.11 (-, 12H, 2 × -C$\underline{H_2}$C$\underline{H_2}$C$\underline{H_2}$-), 1.35 (s, 12H, C(C$\underline{H_3}$)$_2$), 1.04 (d, $^3J_{HH}$ = 6.5 Hz, 3H, -CHC$\underline{H_3}$), 1.02 (d, $^3J_{HH}$ = 6.5 Hz, 3H, -CHC$\underline{H_3}$), 0.87 (d, $^3J_{HH}$ = 6.6 Hz, 6H, -CH(C$\underline{H_3}$)$_2$), 0.86 (d, $^3J_{HH}$ = 6.6 Hz, 6H, -CH(C$\underline{H_3}$)$_2$).

^{13}C NMR (100.6 MHz, CD_2Cl_2, 295 K):

δ [ppm] = 173.4 (quart.), 172.5 (quart.), 172.1 (quart.), 168.1 (quart.), 167.3 (quart.), 167.1 (quart.), 144.8 (quart.), 144.2 (quart.), 142.7 (quart.), 142.2 (quart.), 141.5 (2 × quart.), 136.7 (quart.), 135.5 (tert.), 132.1 (tert.), 128.4 (tert.), 128.1 (2 × tert.), 125.5 (tert.), 120.1 (quart.), 119.05 (quart.), 119.03 (quart.), 118.1 (2 × tert.), 110.3 (tert.), 110.0 (tert.), 106.4 (2 × tert.), 90.0 (tert.), 89.9 (tert.), 84.4 (2 × quart.), 49.8 (quart.), 49.7 (quart.), 43.37 (sec.), 43.36 (sec.), 40.8 (quart.), 39.53 (sec.), 39.52 (sec.), 37.48 (sec.), 37.46 (sec.), 34.41 (sec.), 34.40 (sec.), 31.32 (tert.), 31.29 (tert.), 28.40 (tert.), 28.39 (tert.), 26.85 (prim.), 26.81 (prim.), 26.74 (prim.), 26.70 (prim.), 25.1 (4 × prim.), 25.03 (sec.), 25.01 (sec.), 22.83 (prim.), 22.82 (prim.), 22.74 (prim.), 22.73 (prim.), 19.80 (prim). 19.79 (prim.).

The boron-bound carbon was not found due to quadrupolar relaxation.

MALDI-MS pos (high res.): [$M^{\cdot+}$]

calc.: 1016.665

found: 1016.672

2.1 Synthetic Route of Odd-Numbered Oligomers

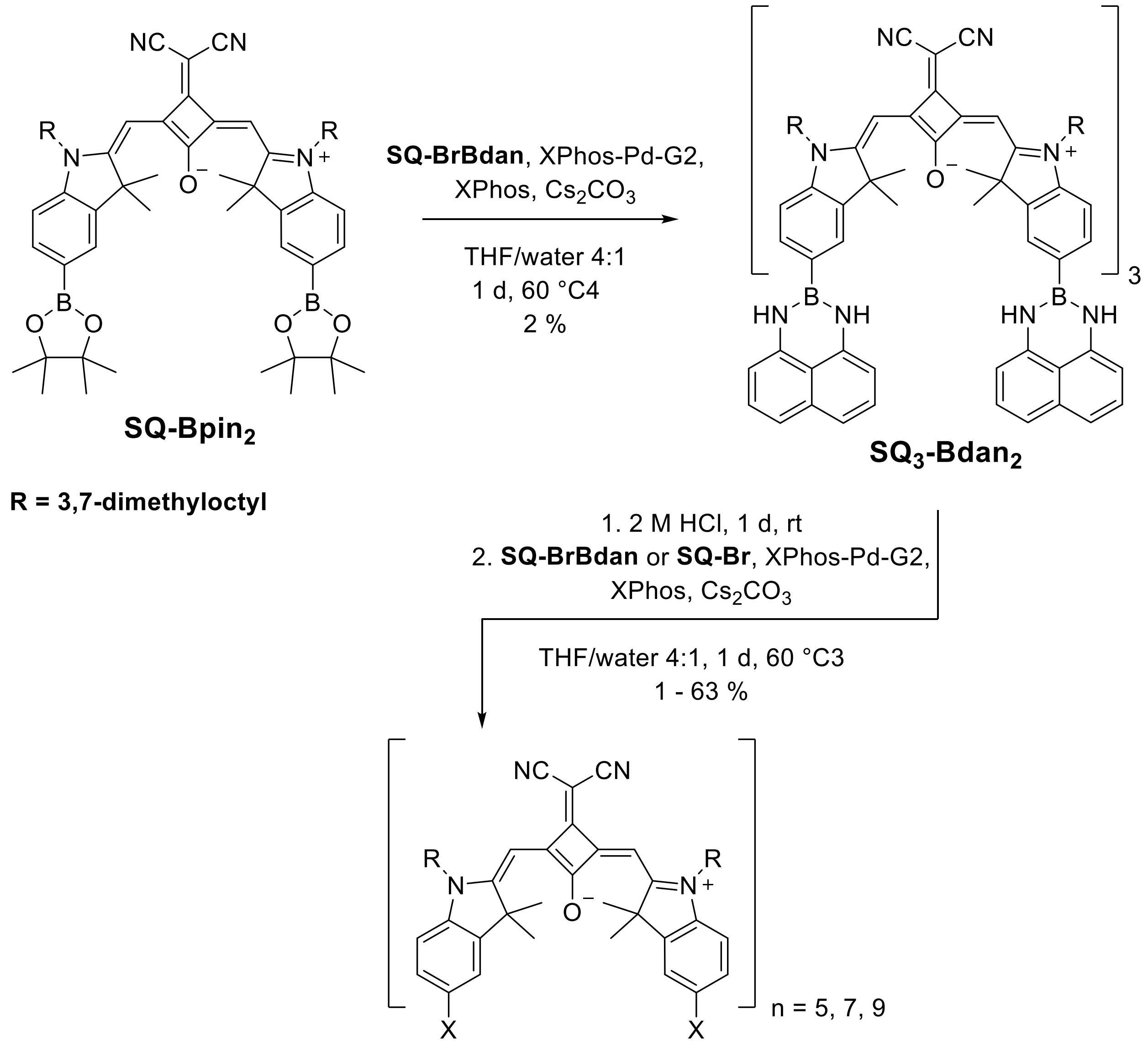

X = H (SQ_n), Bdan (SQ_n-$Bdan_2$, except 9)

Scheme S2. Synthetic route of the odd-numbered oligomers, **SQ_5**, **SQ_7** and **SQ_9**.

Synthesis of **SQ$_3$**

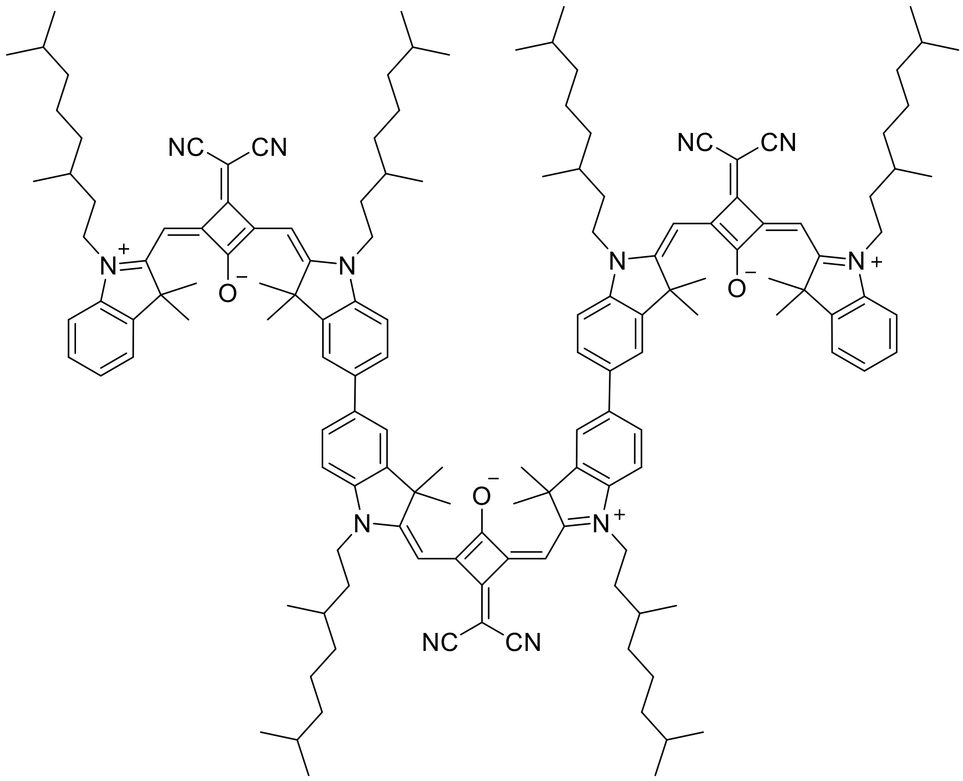

CAS: [1980849-14-6]

Synthesis based on literature.[5]

Under a nitrogen atmosphere, **SQ-Bpin$_2$** (50.0 mg, 51.0 µmol), **SQ-Br** (82.0 mg, 102 µmol), $Pd(PPh_3)_4$ (1.18 mg, 1.02 µmol) and $NaHCO_3$ (172 mg, 2.05 mmol) were dissolved in a degassed 4:1 mixture of THF/water (10 mL). The reaction was heated at 80 °C for 1 d. The solution was cooled to rt and Brine (20 mL) and DCM (20 mL) were added. The organic layer was separated and the aqueous layer extracted with DCM (2 × 20 mL). The combined organic layers were dried over $MgSO_4$, filtrated and the solvent removed *in vacuo*. The residue was purified by flash column chromatography (eluent: DCM + 0.1 → 0.5 % MeOH), subsequent recycling HPLC (DCM + 0.2 % MeOH) and precipitation in *n*-hexane.
Yield: 50.0 mg (23.1 mmol, 45 %) of a green solid.

$C_{147}H_{188}N_{12}O_3$ [2171.14]

^{1}H NMR (400.1 MHz, CD_2Cl_2, 295 K):

δ [ppm] = 7.64 – 7.59 (-, 8H, 8 × -CH-), 7.40 (dd, $^3J_{HH}$ = 7.4 Hz, $^4J_{HH}$ = 0.7 Hz, 2H, 2 × -CH-), 7.37 (td, $^3J_{HH}$ = 7.7 Hz, $^4J_{HH}$ = 0.7 Hz, 2H, 2 × -CH-), 7.22 (td, $^3J_{HH}$ = 7.5 Hz,

$^4J_{HH}$ = 0.7 Hz, 2H, 2 × -CH-), 7.18 – 7.13 (m, 4H, 4 × -CH-), 7.09 (d, $^3J_{HH}$ = 8.0 Hz, 2H, 2 × -CH-), 6.52 (s, 2H, 2 × -CCHC-), 6.50 (s, 2H, 2 × -CCHC-), 6.49 (s, 2H, 2 × -CCHC-), 4.18 – 3.96 (m, 12H, 6 × -NCH_2-), 1.91 – 1.73 (-, 6H, 6 × -NCH_2CH_2-), 1.84 (s, 12H, 2 × -C(CH_3)$_2$), 1.82 (s, 12H, 2 × -C(CH_3)$_2$), 1.76 (s, 12H, 2 × -C(CH_3)$_2$), 1.73 – 1.59 (-, 12H, 6 × -NCH_2CH_2-, 6 × CHCH_3), 1.59 – 1.47 (-, 6H, 6 × -CH(CH_3)$_2$), 1.47 – 1.32 (-, 12H, 6 × -CH_2-), 1.32 – 1.12 (-, 24H, 12 × -CH_2CH_2-), 1.07 – 1.01 (-, 18H, 6 × -CHCH_3), 0.874 (d, $^3J_{HH}$ = 6.6 Hz, 12H, 2 × -CH(CH_3)$_2$), 0.869 (d, $^3J_{HH}$ = 6.6 Hz, 12H, 2 × -CH(CH_3)$_2$), 0.86 (d, $^3J_{HH}$ = 6.6 Hz, 12H, 2 × -CH(CH_3)$_2$).

MALDI-MS pos (high res.): [$M^{\cdot+}$]

calc.: 2170.49541

found: 2170.49263

Synthesis of **SQ$_3$-Bdan$_2$**

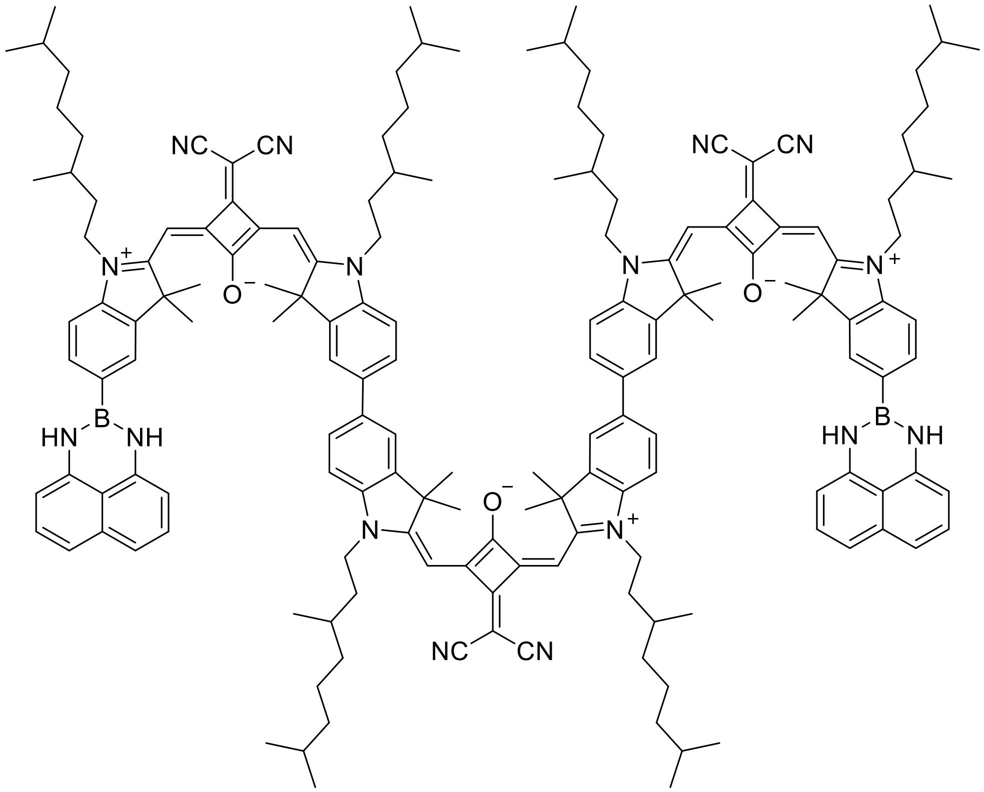

Synthesis following **GP II**:

SQ-Bpin$_2$ (250 mg, 256 µmol), **SQ-BrBdan** (546 mg, 563 µmol), XPhos-Pd-G2 (10 .1 mg, 12.8 µmol), XPhos (12.0 mg, 25.1 µmol), Cs_2CO_3 (333 mg, 1.024 mmol), THF/water 4:1 (50 mL); flash column chromatography (eluent: DCM + 0.1 → 0.7 % MeOH), preparative HPLC (DCM + 0.2 % MeOH), precipitation in *n*-hexane.
Yield: 271 mg (108 µmol, 42 %) of a green solid.

$C_{167}H_{202}B_2N_{16}O_3$ [2502.64]

^{1}H NMR (400.1 MHz, CD_2Cl_2, 295 K):

δ [ppm] = 7.69 – 7.60 (-, 12H, 12 × -CH-), 7.17 (dd, $^3J_{HH}$ = 9.0 Hz, $^4J_{HH}$ = 2.8 Hz, 2H, 2 × -CH-), 7.16 – 7.10 (-, 6H, 2 × -CH-), 7.03 (dd, $^3J_{HH}$ = 8.4 Hz, $^4J_{HH}$ = 0.9 Hz, 2H, 2 × -CH-), 6.55 (s, 2H, 2 × -CCHC-), 6.53 (s, 2H, 2 × -CCHC-), 6.51 (s, 2H, 2 × -CCHC-), 6.48 (dd, $^3J_{HH}$ = 7.3 Hz, $^4J_{HH}$ = 0.9 Hz, 2H, 2 × -CH-), 6.18 (s, 4H, 4 × -NH), 4.22 – 3.96 (-, 12H, 6 × -NCH_2-), 1.91 – 1.74 (-, 30H, 4 × -C(CH_3)$_2$), 6 × -NCH$_2$CH_2), 1.81 (s, 12H, 2 × -C(CH_3)$_2$), 1.74 – 1.59 (-, 12H, 6 × -NCH$_2$CH_2, 6 × -CHCH$_3$), 1.59 – 1.48 (-, 6H, 6 × -CH(CH$_3$)$_2$), 1.48 – 1.32 (-, 12H,

6 × -C$\underline{H}_2$CH$_2$CH$_2$-), 1.32 – 1.12 (-, 24H, 6 × -CH$_2$C$\underline{H}_2$C$\underline{H}_2$-), 1.08 – 1.03 (-, 18H, 6 × -CHC$\underline{H}_3$), 0.88 (d, $^3J_{HH}$ = 6.6 Hz, 12H, 2 × -CH(C$\underline{H}_3$)$_2$), 0.88 – 0.86 (-, 24H, 4 × -CH(C$\underline{H}_3$)$_2$).

^{13}C NMR (100.6 MHz, CD_2Cl_2, 295 K):

δ [ppm] = 173.6 (quart.), 173.5 (2 × quart.), 172.1 (2 × quart.), 171.7 (4 × quart.), 168.1 (2 × quart.), 168.0 (quart.), 167.0 (2 × quart.), 166.6 (2 × quart.), 166.5 (2 × quart.), 144.2 (2 × quart.), 143.84 (2 × quart.), 143.80 (2 × quart.), 142.6 (2 × quart.), 141.9 (2 × quart.), 141.8 (2 × quart.), 141.5 (4 × quart.), 137.8 (2 × quart.), 137.6 (2 × quart.), 136.7 (2 × quart.), 132.1 (2 × tert.), 128.0 (6 × tert.), 127.3 (2 × tert.), 125.5 (2 × tert.), 121.2 (4 × tert.), 120.1 (2 × quart.), 119.11 (2 × quart.), 119.10 (2 × quart.), 119.05 (2 × quart.), 118.1 (4 × tert.), 111.0 (2 × tert.), 110.9 (2 × tert.), 110.3 (2 × tert.), 106.4 (4 × tert.), 90.0 (2 × tert.), 89.9 (2 × tert.), 89.8 (2 × tert.), 50.0 (2 × quart.), 49.9 (2 × quart.), 49.7 (2 × quart.), 43.55 (sec.), 43.43 (sec.), 43.50 (sec.), 43.48 (sec.), 43.33 (sec.), 43.32 (sec.), 40.9 (2 × quart.), 40.8 (quart.), 39.5 (6 × sec.), 37.5 (6 × sec.), 34.49 (2 × sec.), 34.47 (2 × sec.), 34.40 (2 × sec.), 31.3 (6 × tert.), 28.4 (6 × tert.), 26.89 (2 × prim.), 26.86 (2 × prim.), 26.84 (2 × prim.), 26.83 (4 × prim.), 26.79 (2 × prim.), 25.05 (4 × sec.), 25.04 (2 × sec.), 22.8 (6 × prim.), 22.7 (6 × prim.), 19.84 (4 × prim.), 19.81 (2 × prim.).

The boron-bound carbon was not found due to quadrupolar relaxation.

MALDI-MS pos (high res.): [$M^{\cdot +}$]

calc.: 2502.63829

found: 2502.64076

Synthesis of **SQ_5**

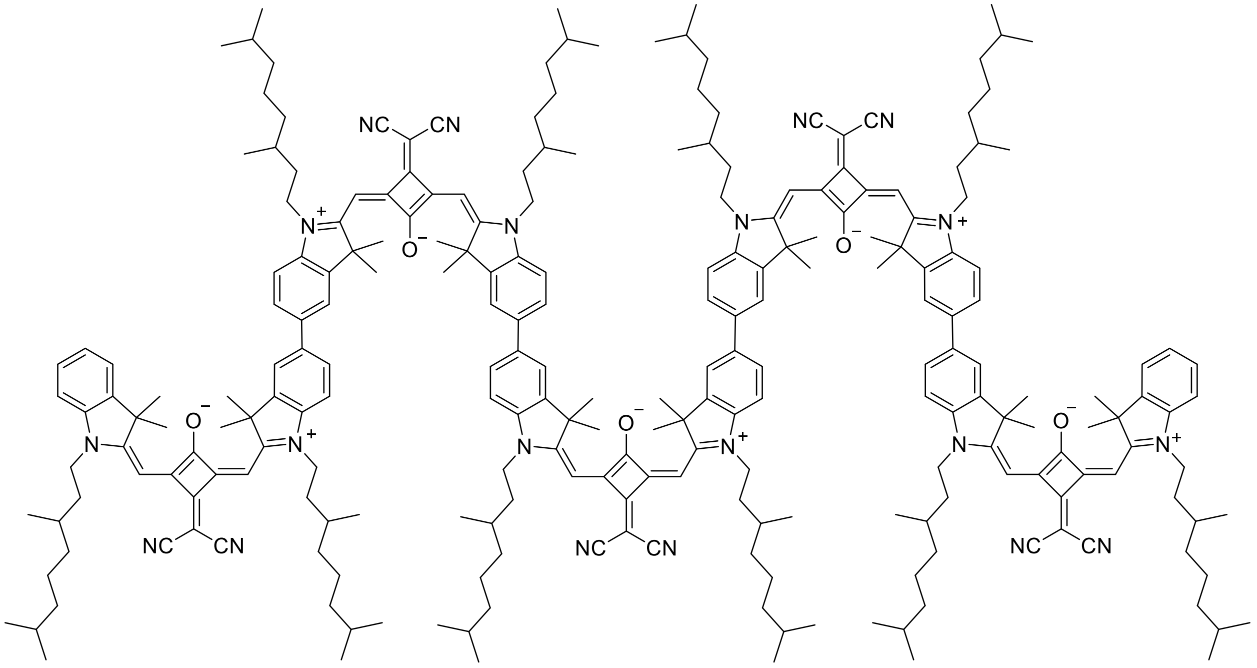

Synthesis following **GP I**:

SQ_3-Bdan$_2$ (50.0 mg, 20.0 µmol), 2 M HCl (240 µmol, 480 mmol), THF (3 mL).

Synthesis following **GP II**:

SQ-Br (35.0 mg, 44.0 µmol), XPhos-Pd-G2 (1.57 mg, 2.00 µmol), XPhos (1.91 mg, 4.00 µmol), Cs_2CO_3 (26.0 mg, 80.0 µmol), THF/water 4:1 (5 mL); flash column chromatography (eluent: DCM + 0.5 % MeOH), preparative recycling GPC ($CHCl_3$), precipitation in *n*-hexane.
Yield: 22.0 mg (6.09 µmol, 31 %) of a green solid.

$C_{245}H_{312}N_{20}O_5$ [3617.33]

^{1}H NMR (600.1 MHz, CD_2Cl_2, 295 K):

δ [ppm] = 7.64 – 7.58 (-, 16H, 16 × -CH-), 7.40 (dd, $^3J_{HH}$ = 7.4 Hz, $^4J_{HH}$ = 0.7 Hz, 2H, 2 × -CH-), 7.36 (td, $^3J_{HH}$ = 7.3 Hz, $^4J_{HH}$ = 1.4 Hz, 2H, 2 × -CH-), 7.21 (td, $^3J_{HH}$ = 7.4 Hz, $^4J_{HH}$ = 0.7 Hz, 2H, 2 × -CH-), 7.19 – 7.15 (-, 6H, 6 × -CH-), 7.13 (d, $^3J_{HH}$ = 8.2 Hz, 2H, 2 × -CH-), 7.09 (d, $^3J_{HH}$ = 8.0 Hz, 2H, 2 × -CH-), 6.531 – 6.524 (-, 4H, 4 × -CCHC-), 6.521 (s, 2H, 2 × -CCHC-), 6.50 (s, 2H, 2 × -CCHC-), 6.48 (s, 2H, 2 × -CCHC-), 4.17 – 3.98 (-, 20H, 20 × -NCH_2-), 1.96 – 1.73 (-, 10H, 10 × -NCH_2CH_2), 1.84 (s, 12H, 2 × -C(CH_3)$_2$), 1.85 – 1.82 (-, 24H, 4 × -C(CH_3)$_2$), 1.82

(s, 12H, 2 × -C(C$\underline{H}_3$)$_2$), 1.76 (s, 12H, 2 × -C(C$\underline{H}_3$)$_2$), 1.73 – 1.59 (-, 20H, 10 × -NCH_2C$\underline{H}_2$, 10 × -C$\underline{H}$CH_3), 1.59 – 1.48 (-, 10H, 10 × -C$\underline{H}$(CH_3)$_2$), 1.48 – 1.13 (-, 60H, 10 × -C$\underline{H}_2$C$\underline{H}_2$C$\underline{H}_2$-), 1.07 – 1.05 (-, 12H, 4 × -CHC$\underline{H}_3$), 1.05 (d, $^3J_{HH}$ = 6.6 Hz, 6H, 2 × -CHC$\underline{H}_3$), 1.04 (d, $^3J_{HH}$ = 6.3 Hz, 6H, 2 × -CHC$\underline{H}_3$), 1.03 (d, $^3J_{HH}$ = 6.4 Hz, 6H, 2 × -CHC$\underline{H}_3$), 0.89 – 0.85 (-, 60H, 10 × -CH(C$\underline{H}_3$)$_2$).

^{13}C NMR (150.9 MHz, CD_2Cl_2, 295 K):

δ [ppm] = 173.57 (quart.), 173.55 (2 × quart.), 173.50 (2 × quart.), 172.4 (2 × quart.), 171.74 (2 × quart.), 171.70 (2 × quart.), 171.6 (2 × quart.), 171.3 (2 × quart.), 168.04 (quart.), 168.03 (2 × quart.), 167.9 (2 × quart.), 167.1 (2 × quart.), 166.6 (2 × quart.), 166.52 (2 × quart.), 166.46 (2 × quart.), 166.2 (2 × quart.), 143.77 (2 × quart.), 143.76 (4 × quart.), 143.71 (2 × quart.), 142.9 (2 × quart.), 142.3 (2 × quart.), 141.95 (2 × quart.), 141.89 (2 × quart.), 141.88 (2 × quart.), 141.83 (2 × quart.), 137.71 (4 × quart.), 137.66 (2 × quart.), 137.4 (2 × quart.), 128.4 (2 × tert.), 127.36 (2 × tert.), 127.34 (2 × tert.), 127.30 (2 × tert.), 127.2 (2 × tert.), 125.0 (2 × tert.), 122.6 (2 × tert.), 121.24 (4 × tert.), 121.16 (4 × tert.), 119.14 (4 × quart.), 119.11 (6 × quart.), 110.91 (2 × tert.), 110.89 (2 × tert.), 110.86 (2 × tert.), 110.7 (2 × tert.), 110.6 (2 × tert.), 89.85 (4 × tert.), 89.81 (2 × tert.), 89.6 (4 × tert.), 49.93 (6 × quart.), 49.90 (2 × quart.), 49.8 (2 × quart.), 43.5 (5 × sec.), 43.4 (5 × sec.), 40.8 (3 × quart.), 40.7 (2 × quart.), 39.5 (10 × sec.), 37.50 (8 × sec.), 37.47 (2 × sec.), 34.5 (5 × sec.), 34.4 (5 × sec.), 31.32 (8 × tert.), 31.30 (2 × tert.), 28.41 (6 × tert.), 28.40 (2 × tert.), 28.39 (2 × tert.), 26.90 (2 × prim.), 26.86 (6 × prim.), 26.84 (2 × prim.), 26.82 (4 × prim.), 26.81 (2 × prim.), 26.62 (2 × prim.), 26.59 (2 × prim.), 25.1 (8 × sec.), 25.0 (2 × sec.), 22.84 (8 × prim.), 22.82 (2 × prim.), 22.75 (8 × prim.), 22.73 (2 × prim.), 19.84 (8 × prim.), 19.78 (2 × prim.).

ESI-MS pos (high res.): [$M^{\cdot 2+}$] + 2 Na^+

calc.: 1831.23117

found: 1831.23364 Δ = -1.35 ppm

Synthesis of **SQ_5-Bdan$_2$**

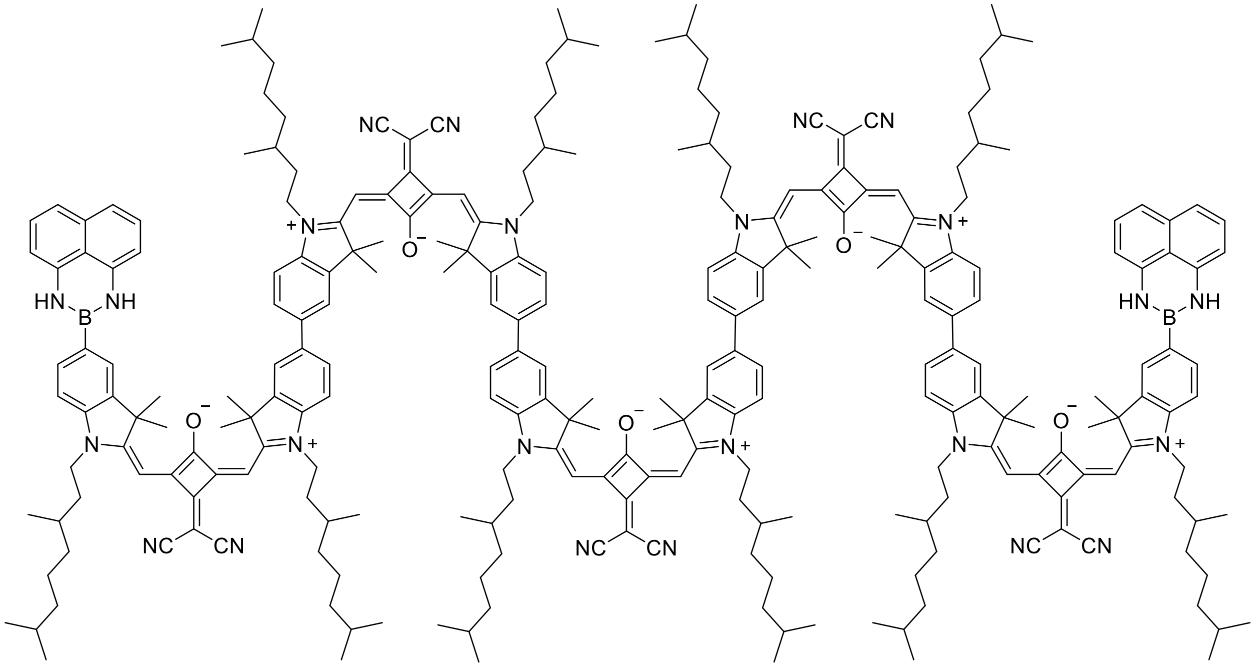

Synthesis following **GP I**:

SQ_3-Bdan$_2$ (230 mg, 92.0 µmol), 2 M HCl (1.10 mL, 2.21 mmol), THF (10 mL).

Synthesis following **GP II**:

SQ-BrBdan (214 mg, 221 µmol), XPhos-Pd-G2 (7.24 mg, 9.20 µmol), XPhos (8.77 mg, 18.4 µmol), Cs_2CO_3 (120 mg, 368 µmol), THF/water (15 mL); flash column chromatography (eluent: DCM + 0.1 $\rightarrow$ 0.4 % MeOH), precipitation in *n*-hexane.

Yield: 194 mg (49.0 µmol, 54 %) of a green solid.

$C_{265}H_{326}B_2N_{24}O_5$ [3949.31]

^{1}H NMR (400.1 MHz, CD_2Cl_2, 295 K):

δ [ppm] = 7.71 – 7.67 (-, 4H,4 × -C$\underline{H}$-), 7.65 – 7.58 (16H, 16 × -C$\underline{H}$-), 7.19 – 7.11 (-, 14H, 14 × -C$\underline{H}$-), 7.03 (dd, $^3J_{HH}$ = 8.4 Hz, $^4J_{HH}$ = 0.8 Hz, 4H, 4 × -C$\underline{H}$-), 6.55 – 6.51 (-, 10H, 10 × -CC$\underline{H}$C-), 6.49 (dd, $^3J_{HH}$ = 7.3 Hz, $^4J_{HH}$ = 0.9 Hz, 4H, 4 × -C$\underline{H}$-), 6.23 (s, 4H, 4 × -N$\underline{H}$), 4.19 – 3.98 (-, 20H, 10 × -NC$\underline{H_2}$-), 1.91 – 1.74 (-, 10H, 10 × -NCH$_2$C$\underline{H_2}$-), 1.84 (-, 36H, 6 × -C(C$\underline{H_3}$)$_2$), 1.83 (s, 12H, 2 × -C(C$\underline{H_3}$)$_2$), 1.81 (s, 12H, 2 × -C(C$\underline{H_3}$)$_2$), 1.74 – 1.59 (-, 20H, 10 × -NCH$_2$C$\underline{H_2}$, 10 × C$\underline{H}$CH$_3$), 1.59 – 1.47 (-, 10H, 10 × -C$\underline{H}$(CH$_3$)$_2$), 1.47 – 1.12 (-, 60H, 10 × -C$\underline{H_2}$C$\underline{H_2}$C$\underline{H_2}$-), 1.07 – 1.03 (-, 30H, 10 × -CHC$\underline{H_3}$), 0.88 (d, $^3J_{HH}$ = 6.6 Hz, 12H, 2 × CH(C$\underline{H_3}$)$_2$), 0.87 (d, $^3J_{HH}$ = 6.6 Hz, 12H, 2 × CH(C$\underline{H_3}$)$_2$), 0.88 – 086 (-, 36H, 6 × CH(C$\underline{H_3}$)$_2$).

^{13}**C NMR** (100.6 MHz, CD_2Cl_2, 295 K):

δ [ppm] = 173.56 (quart.), 173.55 (2 × quart.), 173.5 (2 × quart.), 172.0 (2 × quart.), 171.72 (2 × quart.), 171.68 (6 × quart.), 168.05 (4 × quart.), 168.03 (quart.), 167.0 (2 × quart.), 166.6 (2 × quart.), 166.5 (6 × quart.), 144.3 (2 × quart.), 143.83 (2 × quart.), 143.77 (2 × quart.), 143.74 (4 × quart.), 142.7 (2 × quart.), 141.90 (2 × quart.), 141.87 (2 × quart.), 141.85 (2 × quart.), 141.79 (2 × quart.), 141.5 (4 × quart.), 137.8 (2 × quart.), 137.7 (4 × quart.), 137.6 (2 × quart.), 136.7 (2 × quart.), 132.1 (2 × tert.), 128.0 (4 × tert.), 127.4 (8 × tert.), 125.5 (2 × tert.), 121.2 (8 × tert.), 120.1 (2 × quart.), 119.1 (8 × quart.), 119.0 (2 × quart.), 118.1 (4 × tert.), 111.0 (2 × tert.), 110.9 (6 × tert.), 110.3 (2 × tert.), 106.4 (4 × tert.), 90.0 (2 × tert.), 89.9 (6 × tert.), 89.8 (2 × tert.), 50.0 (2 × quart.), 49.9 (6 × quart.), 49.7 (2 × quart.), 43.5 (8 × sec.), 43.3 (2 × sec.), 40.9 (2 × quart.), 40.8 (3 × quart.), 39.54 (10 × sec.), 37.49 (10 × sec.), 34.5 (8 × sec.), 34.4 (2 × sec.), 31.3 (10 × tert.), 28.4 (10 × tert.), 26.90 (2 × prim.), 26.86 (8 × prim.), 26.82 (8 × prim.), 26.78 (2 × prim.), 25.1 (5 × sec.), 25.0 (5 × sec.), 22.84 (10 × prim.), 22.75 (10 × prim.), 19.84 (6 × prim.), 19.83 (2 × prim.), 19.81 (2 × prim.).

The boron-bound carbon was not found due to quadrupolar relaxation.

MALDI-MS pos (high res.): [$M^{\cdot+}$]

calc.: 3948.62463

found: 3948.63705

Synthesis of **SQ$_7$**

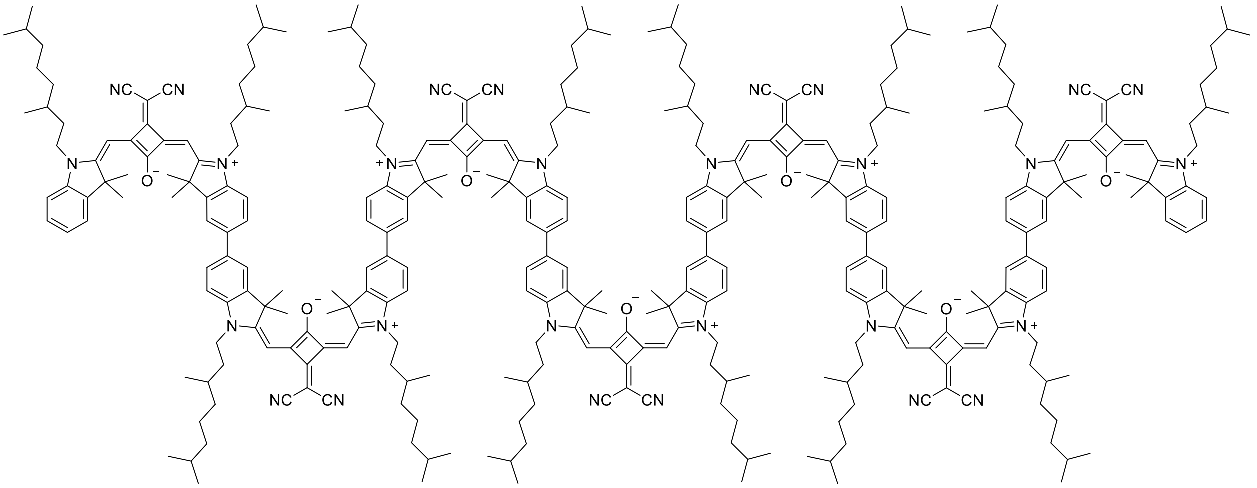

Synthesis following **GP I**:

SQ$_5$-Bdan$_2$ (64.0 mg, 16.0 µmol), 2 M HCl (195 µL, 389 µmol), THF (3 mL).

Synthesis following **GP II**:

SQ-Br (31.0 mg, 39.0 µmol), XPhos-Pd-G2 (1.28 mg, 1.62 µmol), XPhos (1.55 mg, 3.24 µmol), Cs_2CO_3 (21.0 mg, 65.0 µmol), THF/water 4:1 (5 mL); flash column chromatography (eluent: DCM + 0.1 → 0.4 % MeOH), recycling HPLC (eluent: DCM + 0.2 % MeOH), precipitation in *n*-hexane.

Yield: 38.0 mg (7.51 µmol, 46 %) of a green solid.

$C_{343}H_{436}N_{28}O_7$ [5063.45]

^{1}H NMR (600.1 MHz, CD_2Cl_2, 295 K):

δ [ppm] = 7.64 – 7.58 (-, 24H, 24 × -C<u>H</u>-), 7.40 (dd, $^3J_{HH}$ = 7.5 Hz, $^4J_{HH}$ = 0.8 Hz, 2H, 2 × -C<u>H</u>-), 7.36 (td, $^3J_{HH}$ = 7.7 Hz, $^4J_{HH}$ = 1.1 Hz, 2H, 2 × -C<u>H</u>-), 7.21 (td, $^3J_{HH}$ = 7.5 Hz, $^4J_{HH}$ = 0.5 Hz, 2H, 2 × -C<u>H</u>-), 7.19 – 7.11 (-, 12H, 12 × -C<u>H</u>-), 7.09 (d, $^3J_{HH}$ = 8.0 Hz, 2H, 2 × -C<u>H</u>-), 6.53 – 6.522 (-, 4H, 4 × -CC<u>H</u>C-), 6.520 (s, 2H, 2 × -CC<u>H</u>C-), 6.517 – 6.504 (-, 4H, 4 × -CC<u>H</u>C-), 6.496 (s, 2H, 2 × -CC<u>H</u>C-), 6.48 (s, 2H, 2 × -CC<u>H</u>C-), 4.17 – 3.99 (-, 28H, 14 × -NC<u>H$_2$</u>-), 1.90 – 1.73 (-, 74H, 14 × -NCH$_2$C<u>H</u>$_2$, 10 × -C(C<u>H$_3$</u>)$_2$), 1.81 (s, 12H, 2 × -C(C<u>H$_3$</u>)$_2$), 1.76 (s, 12H, 2 × -C(C<u>H$_3$</u>)$_2$), 1.73 – 1.58 (-, 28H, 14 × -NCH$_2$C<u>H</u>$_2$, 14 × -C<u>H</u>CH$_3$), 1.58 – 1.47 (-, 14H, 14 × -C<u>H</u>(CH$_3$)$_2$), 1.48 – 1.32 (-, 28H, 14 × -C<u>H$_2$</u>-), 1.32 – 1.20 (-, 28H, 14 × -C<u>H$_2$</u>-),1.20 – 1.13 (-, 28H, 14 × -C<u>H$_2$</u>-), 1.07 – 1.02 (-, 42H, 14 × -CHC<u>H$_3$</u>), 0.89 – 0.85 (-, 84H, 14 × -CH(C<u>H$_3$</u>)$_2$).

^{13}C NMR (150.9 MHz, CD_2Cl_2, 295 K):

δ [ppm] = 173.6 (5 × quart.), 173.5 (2 × quart.), 172.5 (2 × quart.), 171.8 (2 × quart.), 171.72 (2 × quart.), 171.67 (4 × quart.), 171.6 (2 × quart.), 171.3 (2 × quart.), 168.0 (5 × quart.), 167.9 (2 × quart.), 167.1 (2 × quart.), 166.58 (2 × quart.), 166.55 (2 × quart.), 166.52 (2 × quart.), 166.50 (2 × quart.), 166.46 (2 × quart.), 166.2 (2 × quart.), 143.77 (10 × quart.), 143.72 (2 × quart.), 143.0 (2 × quart.), 142.3 (2 × quart.), 142.0 (2 × quart.), 141.9 (8 × quart.), 141.8 (2 × quart.), 137.71 (4 × quart.), 137.66 (6 × quart.), 137.4 (2 × quart.), 128.4 (2 × tert.), 127.34 (10 × tert.), 127.25 (2 × tert.), 125.0 (2 × tert.), 122.6 (2 × tert.), 121.22 (8 × tert.), 121.17 (4 × tert.), 119.13 (2 × quart.), 119.10 (12 × quart.), 110.9 (10 × tert.), 110.7 (2 × tert.), 110.6 (2 × tert.), 89.9 (10 × tert.), 89.6 (4 × tert.), 49.94 (6 × quart.), 49.92 (4 × quart.), 49.91 (2 × quart.), 49.8 (2 × quart), 43.5 (10 × sec.), 43.4 (4 × sec.), 40.82 (3 × quart.), 40.80 (2 × quart.), 40.7 (2 × quart.), 39.5 (14 × sec.), 37.50 (12 × sec.), 37.48 (2 × sec.), 34.5 (10 × sec.), 34.4 (4 × sec.), 31.3 (14 × tert.), 28.4 (14 × tert.), 26.90 (2 × prim.), 26.86 (12 × prim.), 26.8 (10 × prim.), 26.63 (2 × prim.), 26.60 (2 × prim.), 25.1 (12 × sec.), 25.0 (2 × sec.), 22.84 (12 × prim.), 22.82 (2 × prim.), 22.75 (12 × prim.), 22.74 (2 × prim.), 19.84 (12 × prim.), 19.79 (2 × prim.).

ESI-MS pos (high res.): $[M^{\cdot\cdot\cdot\cdot\cdot 5+}]$

calc.: 1012.49382

found: 1012.49330 Δ = 0.51 ppm

Synthesis of **SQ_7-Bdan$_2$**

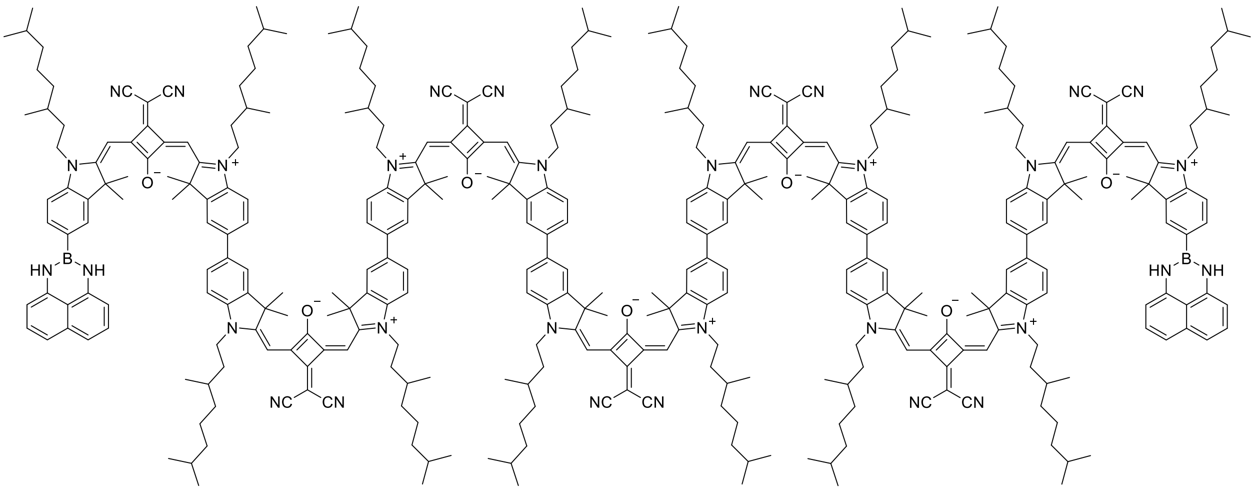

Synthesis following **GP I**:

SQ_5-Bdan$_2$ (130 mg, 33.0 µmol), 2 M HCl (396 µL, 791 µmol), THF (3 mL).

Synthesis following **GP II**:

SQ-BrBdan (77.0 mg, 79.0 µmol), XPhos-Pd-G2 (2.59 mg, 3.30 µmol), XPhos (3.14 mg, 6.59 µmol), Cs_2CO_3 (43.0 mg, 132 µmol), THF/water 4:1 (10 mL); flash column chromatography (eluent: DCM + 0.1 → 0.5 % MeOH), recycling HPLC (eluent: DCM + 0.2 % MeOH), precipitation in *n*-hexane.

Yield: 111 mg (20.6 µmol, 63 %) of a green solid.

$C_{363}H_{450}B_2N_{32}O_7$ [5395.43]

^{1}H NMR (600.1 MHz, CD_2Cl_2, 295 K):

δ [ppm] = 7.70 – 7.66 (-,4H, 4 × -CH-), 7.64 – 7.56 (-, 24H, 24 × -CH-), 7.19 – 7.11 (-, 18H, 18 × -CH-), 7.03 (d, $^3J_{HH}$ = 8.0 Hz, 4H, 4 × -CH-), 6.54 – 6.50 (-, 14H, 14 × -CCHC-), 6.49 (d, $^3J_{HH}$ = 7.3 Hz, 4H, 4 × -CH-), 6.21 (s, 4H, 4 × -NH), 4.17 – 4.00 (-, 28H, 14 × -NCH$_2$-), 1.95 – 1.74 (-, 62H, 14 × -NCH$_2$CH$_2$, 8 × -C(CH$_3$)$_2$), 1.822 (s, 12H, 2 × -C(CH$_3$)$_2$), 1.815 (s, 12H, 2 × -C(CH$_3$)$_2$), 1.810 (s, 12H, 2 × -C(CH$_3$)$_2$), 1.74 – 1.58 (-, 28H, 14 × -NCH$_2$CH$_2$, 14 × -CHCH$_3$), 1.58 – 1.48 (-, 14H, 14 × -CH(CH$_3$)$_2$), 1.45 – 1.32 (-, 28H, 14 × -CH$_2$-), 1.32 – 1.20 (-, 28H, 14 × -CH$_2$-), 1.20 – 1.12 (-, 28H, 14 × -CH$_2$-), 1.07 – 1.02 (-, 42H, 14 × -CHCH$_3$), 0.89 – 0.85 (-, 84H, 14 × -CH(CH$_3$)$_2$).

^{13}C NMR (150.9 MHz, CD_2Cl_2, 295 K):

δ [ppm] = 173.55 (5 × quart.), 173.47 (2 × quart.), 172.0 (2 × quart.), 171.72 (2 × quart.), 171.70 (4 × quart.), 171.67 (4 × quart.), 171.65 (2 × quart.), 168.06 (2 × quart.),

168.04 (5 × quart.), 166.6 (2 × quart.), 166.53 (2 × quart.), 166.52 (2 × quart.), 166.51 (2 × quart.), 166.49 (4 × quart.), 166.46 (2 × quart.), 144.3 (2 × quart.), 143.83 (2 × quart.), 143.76 (10 × quart.), 142.7 (2 × quart.), 141.90 (2 × quart.), 141.87 (8 × quart.), 141.79 (2 × quart.), 141.5 (4 × quart.), 137.8 (2 × quart.), 137.7 (8 × quart.), 137.6 (2 × quart.), 136.7 (2 × quart.), 128.0 (4 × tert.), 127.3 (12 × tert.), 125.5 (2 × tert.), 121.2 (14 × tert.), 120.1 (2 × quart.), 119.1 (14 × quart.), 118.1 (4 × tert.), 111.0 (2 × tert.), 110.9 (10 × tert.), 110.3 (2 × tert.), 106.4 (4 × tert.), 90.0 (2 × tert.), 89.8 (12 × tert.), 50.0 (2 × quart.), 49.92 (6 × quart.), 49.91 (4 × quart.), 49.7 (2 × quart.), 43.5 (12 × sec.), 43.3 (2 × sec.), 40.9 (2 × quart.), 40.8 (5 × quart.), 39.5 (14 × sec.), 37.5 (14 × sec.), 34.47 (6 × sec.), 34.45 (6 × sec.), 34.41 (2 × sec.), 31.3 (14 × tert.), 28.4 (14 × tert.), 26.90 (2 × prim.), 26.85 (12 × prim.), 26.81 (12 × prim.), 26.77 (2 × prim.), 25.0 (14 × sec.), 22.8 (14 × prim.), 22.7 (14 × prim.), 19.83 (12 × prim.), 19.81 (2 × prim.).

The boron-bound carbon was not found due to quadrupolar relaxation.

MALDI-MS pos: [$M^{\cdot +}$]

calc.: 5394.615

found: 5394.708

Synthesis of **SQ_9**

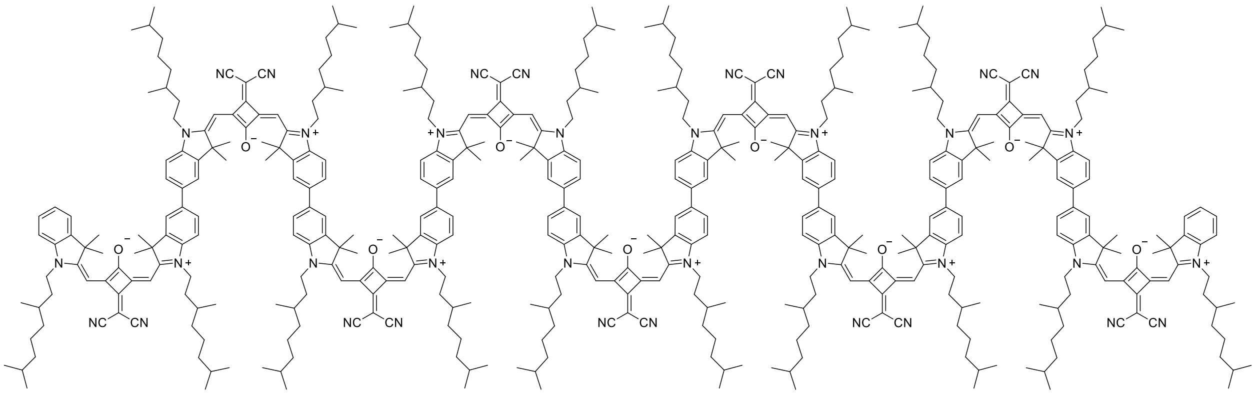

Synthesis following **GP I**:

SQ_7-Bdan$_2$ (100 mg, 19.0 µmol), 2 M HCl (223 µL, 445 µmol), THF (3 mL).

Synthesis following **GP II**:

SQ-Br (36.0 mg, 45.0 µmol), XPhos-Pd-G2 (1.46 mg, 1.86 µmol), XPhos (1.77 mg, 3.71 µmol), Cs_2CO_3 (24.0 mg, 74.0 µmol), THF/water 4:1 (7.5 mL); flash column chromatography (eluent: DCM + 0.1 → 0.4 % MeOH), recycling HPLC (DCM + 0.2 % MeOH), precipitation in *n*-hexane.

Yield: 70.0 mg (10.8 µmol, 58 %) of a green solid.

$C_{441}H_{560}N_{36}O_9$ [6509.57]

^{1}H NMR (600.1 MHz, CD_2Cl_2, 295 K):

δ [ppm] = 7.64 – 7.57 (-, 32H, 32 × -C$\underline{H}$-), 7.40 (d, $^3J_{HH}$ = 7.4 Hz, 2H, 2 × -C$\underline{H}$-), 7.36 (td, $^3J_{HH}$ = 7.7 Hz, $^4J_{HH}$ = 1.1 Hz, 2H, 2 × -C$\underline{H}$-), 7.21 (t, $^3J_{HH}$ = 7.4 Hz, 2H, 2 × -C$\underline{H}$-), 7.19 – 7.10 (-, 16H, 16 × -C$\underline{H}$-), 7.09 (d, $^3J_{HH}$ = 8.0 Hz, 2H, 2 × -C$\underline{H}$-), 6.68 – 6.32 (-, 18H, 18 × -CC$\underline{H}$C-), 4.18 – 3.97 (-, 36H, 18 × -NC$\underline{H_2}$-), 1.95 – 1.73 (-, 102H, 18 × -NCH$_2$C$\underline{H_2}$, 14 × -C(C$\underline{H_3}$)$_2$), 1.81 (s, 12H, 2 × -C(C$\underline{H_3}$)$_2$), 1.76 (s, 12H, 2 × -C(C$\underline{H_3}$)$_2$), 1.73 – 1.61 (-, 36H, 18 × -NCH$_2$C$\underline{H}_2$, 18 × -C$\underline{H}$CH$_3$), 1.61 – 1.48 (-, 18H, 18 × -C$\underline{H}$(CH$_3$)$_2$), 1.46 – 1.32 (-, 36H, 18 × -C$\underline{H_2}$-), 1.32 – 1.21 (-, 36H, 18 × -C$\underline{H_2}$-), 1.21 – 1.12 (-, 36H, 18 × -C$\underline{H_2}$-), 1.08 – 1.01 (-, 54H, 18 × -CHC$\underline{H_3}$), 0.89 – 0.84 (-, 108H, 18 × -CH(C$\underline{H_3}$)$_2$).

^{13}C NMR (150.9 MHz, CD_2Cl_2, 295 K):

δ [ppm] = 173.55 (7 × quart.), 173.49 (2 × quart.), 171.7 (18 × quart.), 168.0 (7 × quart.), 167.9 (2 × quart.), 166.5 (18 × quart.), 143.8 (18 × quart.), 143.0 (2 × quart.), 141.9 (16 × quart.), 137.71 (4 × quart.), 137.66 (10 × quart.), 137.4 (2 × quart.), 128.4 (2 × tert.), 127.3 (14 × tert.), 127.2 (2 × tert.), 125.0 (2 × tert.), 122.6 (2 × tert.), 121.2 (16 × tert.), 119.1 (18 × quart.), 110.9 (14 × tert.), 110.7

(2 × tert.), 110.6 (2 × tert.), 89.9 (18 × tert.), 49.93 (6 × quart.), 49.91 (10 × quart.), 49.79 (2 × quart.), 43.5 (12 × sec.), 43.4 (6 × sec.), 40.8 (9 × quart.), 39.5 (18 × sec.), 37.50 (16 × sec.), 37.47 (2 × sec.), 34.5 (18 × sec.), 31.3 (18 × tert.), 28.4 (18 × tert.), 26.9 (32 × prim.), 26.6 (4 × prim.), 25.05 (16 × sec.), 25.02 (2 × sec.), 22.84 (16 × prim.), 22.82 (2 × prim.), 22.75 (16 × prim.), 22.73 (2 × prim.), 19.83 (16 × prim.), 19.78 (2 × prim.).

ESI-MS pos (high res.): $[M^{\cdot\cdot\cdot\cdot\cdot\cdot 6+}]$

calc.:	1084.74275	
found:	1084.74072	Δ = 1.87 ppm

2.2 Synthetic Route of Even-Numbered Oligomers

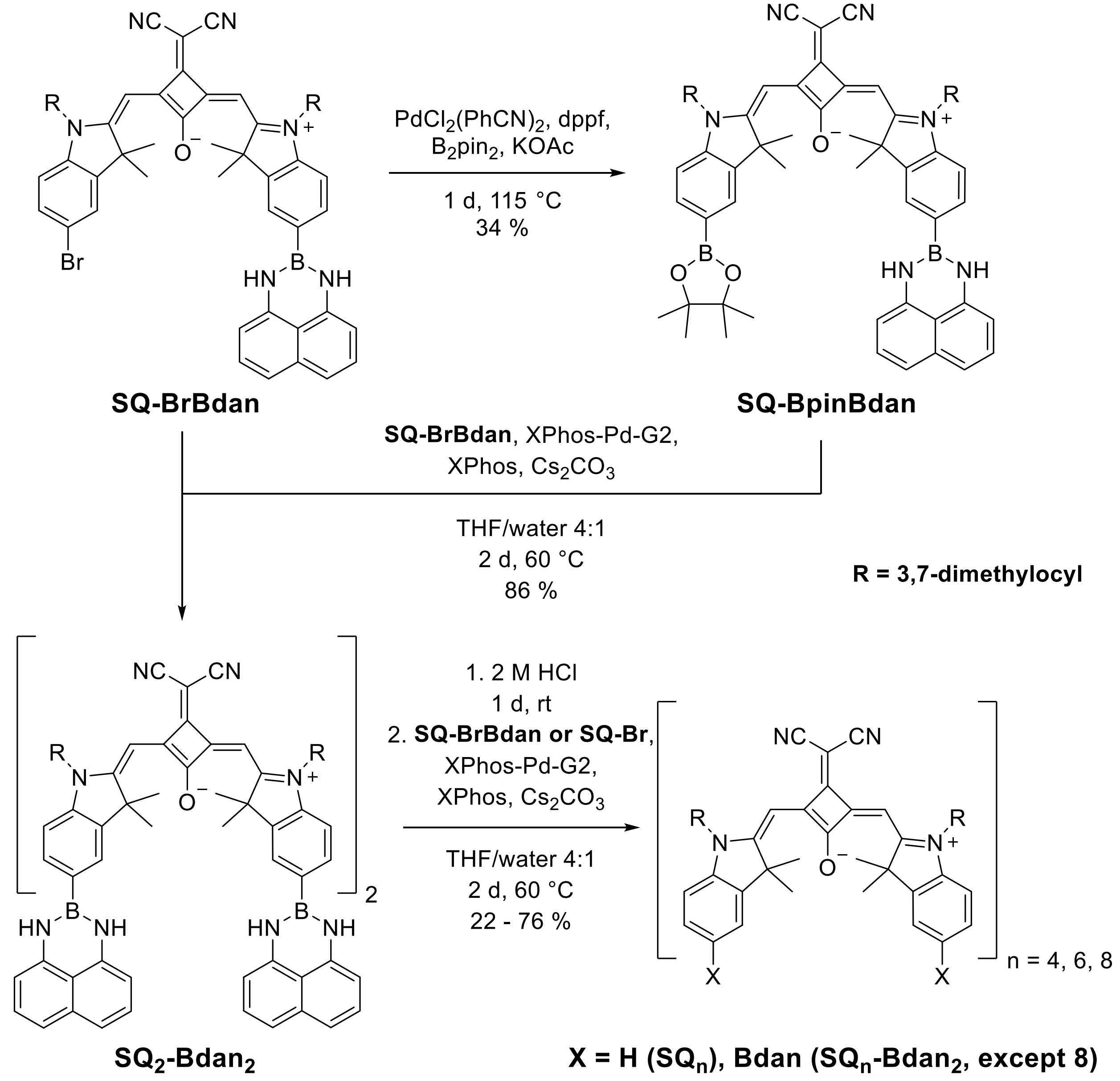

Scheme S3. Synthetic route of the even-numbered oligomers, **SQ$_4$**, **SQ$_6$** and **SQ$_8$**.

Synthesis of **SQ_2-$Bdan_2$**

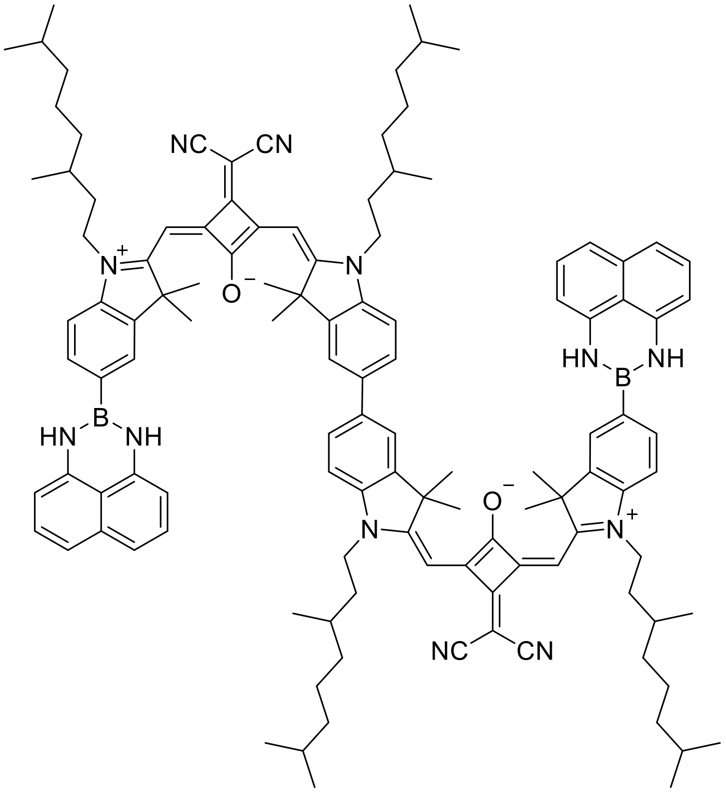

Synthesis following **GP II**:

SQ-BrBdan (269 mg, 277 µmol), **SQ-BpinBdan** (296 µL, 291 µmol), XPhos-Pd-G2 (22.0 mg, 28.0 µmol), XPhos (26.0 mg, 55.0 µmol), Cs_2CO_3 (361 mg, 1.11 µmol), THF/water 4:1 (25 mL); flash column chromatography (eluent: DCM + 0.5 → 0.75 % MeOH), precipitation in *n*-hexane.
Yield: 422 mg (237 µmol, 86 %) of a green solid.

$C_{118}H_{140}B_2N_{12}O_2$ [1780.12]

^{1}H NMR (400.1 MHz, CD_2Cl_2, 295 K):

δ [ppm] = 7.70 – 7.66 (-, 4H, 4 × -C<u>H</u>-), 7.66 – 7.61(-, 4H, 4 × -C<u>H</u>-), 7.20 – 7.13 (-, 8H, 8 × -C<u>H</u>-), 7.06 (dd, $^3J_{HH}$ = 8.4 Hz, $^4J_{HH}$ = 0.8 Hz, 4H, 4 × -C<u>H</u>-), 6.55 (s, 2H, 2 × -CC<u>H</u>C-), 6.52 (s, 2H, 2 × -CC<u>H</u>C-), 6.50 (dd, $^3J_{HH}$ = 7.3 Hz, $^4J_{HH}$ = 0.9 Hz, 4H, 4 × -C<u>H</u>-), 6.20 (s, 4H, 4 × -N<u>H</u>), 4.19 – 3.98 (-, 8H, 4 × -NC<u>H</u>$_2$-), 1.95 – 1.74 (-, 4H, 4 × -NCH$_2$C<u>H</u>$_2$-), 1.84 (s, 12H, 2 × -C(C<u>H</u>$_3$)$_2$), 1.82 (s, 12H, 2 × -C(C<u>H</u>$_3$)$_2$), 1.74 – 1.59 (-, 8H, 4 × -NCH$_2$C<u>H</u>$_2$-, 4 × -C<u>H</u>CH$_3$), 1.59 – 1.47 (-, 4H, 4 × -C<u>H</u>(CH$_3$)$_2$), 1.46 – 1.12 (-, 24H, 4 × -C<u>H</u>$_2$C<u>H</u>$_2$C<u>H</u>$_2$-), 1.06 (d, $^3J_{HH}$ = 6.2 Hz, 2 × -CHC<u>H</u>$_3$), 1.05 (d, $^3J_{HH}$ = 6.3 Hz, 2 × -CHC<u>H</u>$_3$), 0.89 – 0.86 (-, 24H, 4 × -CH(C<u>H</u>$_3$)$_2$).

^{13}**C NMR** (100.6 MHz, CD_2Cl_2, 295 K):

δ [ppm] = 173.5 (2 × quart.), 172.1 (2 × quart.), 171.7 (2 × quart.), 168.1 (2 × quart.), 167.1 (2 × quart.), 166.6 (2 × quart.), 144.3 (2 × quart.), 143.9 (2 × quart.), 142.7 (2 × quart.), 141.8 (2 × quart.), 141.5 (4 × quart.), 137.8 (2 × quart.), 136.7 (2 × quart.), 132.1 (2 × tert.), 128.1 (4 × tert.), 127.4 (2 × tert.), 125.5 (2 × tert.), 121.2 (2 × tert.), 120.1 (4 × quart.), 119.1 (2 × quart.), 118.1 (4 × tert.), 111.0 (2 × tert.), 110.3 (2 × tert.), 106.4 (4 × tert.), 90.0 (2 × tert.), 89.8 (2 × tert.), 50.0 (2 × quart.), 49.7 (2 × quart.), 43.4 (2 × sec.), 43.3 (2 × sec.), 40.9 (2 × quart.), 39.5 (4 × sec.), 37.5 (4 × sec.), 34.5 (2 × sec.), 34.4 (2 × sec.), 31.3 (4 × tert.), 28.4 (4 × tert.), 26.90 (2 × prim.), 26.86 (2 × prim.), 26.82 (2 × prim.), 26.78 (2 × prim.), 25.1 (2 × sec.), 25.0 (2 × sec.), 22.8 (4 × prim.), 22.7 (4 × prim.), 19.83 (2 × prim.), 19.81 (2 × prim.).

The boron-bound carbon was not found due to quadrupolar relaxation.

MALDI-MS pos (high res.): [$M^{\cdot+}$]

calc.: 1779.14335

found: 1779.12040

Synthesis of **SQ_4**

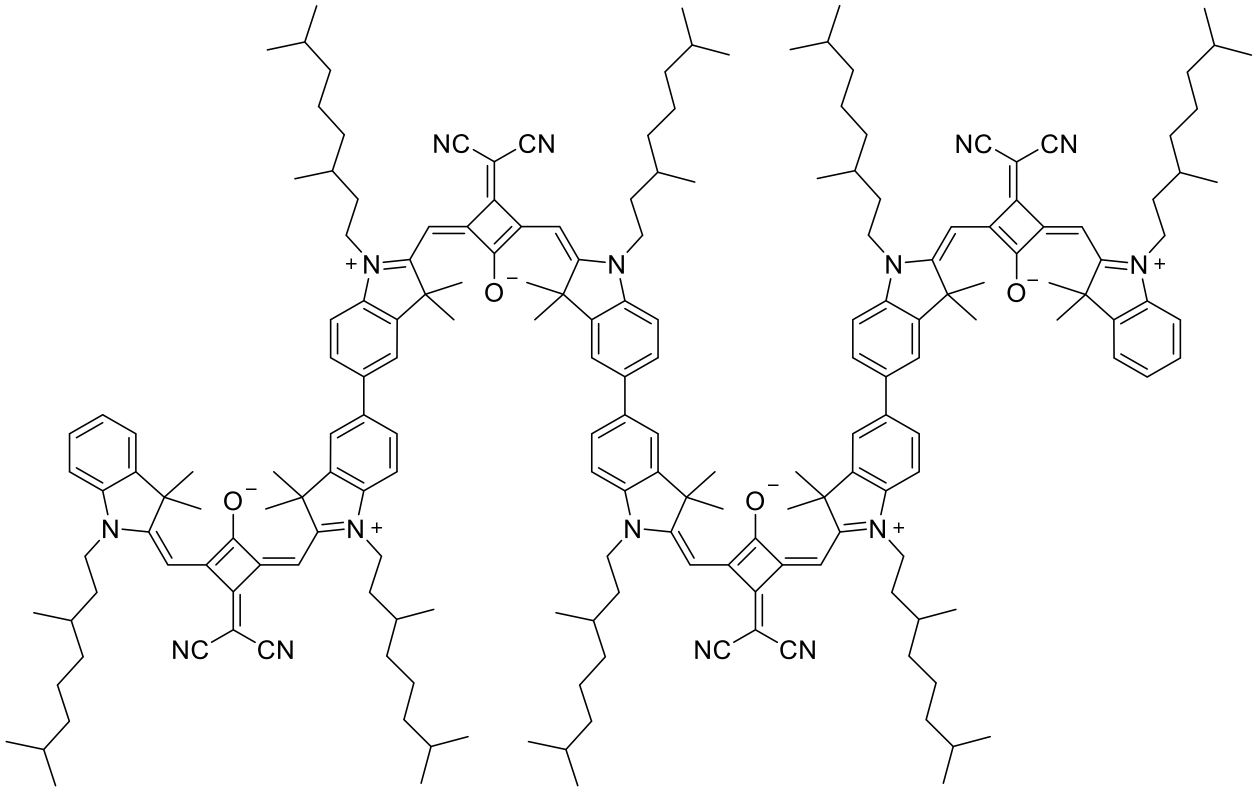

Synthesis following **GP I**:

SQ_2-Bdan$_2$ (80.0 mg, 45.0 µmol), 2 M HCl (540 µL, 1.08 mmol), THF (3 mL).

Synthesis following **GP II**:

SQ-Br (80.0 mg, 99.5 µmol), XPhos-Pd-G2 (3.54 mg, 4.50 µmol), XPhos (4.29 mg, 9.00 µmol), Cs_2CO_3 (58.6 mg, 180 µmol), THF/water 4:1 (5 mL); flash column chromatography (eluent: DCM + 0.1 → 0.4 % MeOH), precipitation in *n*-hexane.
Yield: 40.0 mg (1.38 µmol, 31 %) of a green solid.

$C_{196}H_{250}N_{16}O_4$ [2894.26]

^{1}H NMR (600.1 MHz, CD_2Cl_2, 295 K):

δ [ppm] = 7.63 – 7.58 (-, 12H, 12 × -CH-), 7.40 (d, $^3J_{HH}$ = 7.3 Hz 2H, 2 × -CH-), 7.36 (td, $^3J_{HH}$ = 7.7 Hz, $^4J_{HH}$ = 1.1 Hz, 2H, 2 × -CH-), 7.22 (td, $^3J_{HH}$ = 7.4 Hz, $^4J_{HH}$ = 0.5 Hz, 2H, 2 × -CH-), 7.18 – 7.15 (-, 4H, 4 × -CH-), 7.14 (d, $^3J_{HH}$ = 8.2 Hz, 2H, 2 × -CH-), 7.1 (d, $^3J_{HH}$ = 8.0 Hz, 2H, 2 × -CH-), 6.53 – 6.52 (-, 4H, 4 × -CCHC-), 6.50 (s, 2H, 2 × -CCHC-), 6.49 (s, 2H, 2 × -CCHC-), 4.16 – 3.99 (-, 16H, 8 × -NCH$_2$-), 1.92 –

1.73 (-, 32H, 8 × $-NCH_2C\underline{H_2}-$, 4 × $-C(C\underline{H_3})_2$), 1.82 (s, 12H, 2 × $-C(C\underline{H_3})_2$), 1.76 (s, 12H, 4 × $-C(C\underline{H_3})_2$), 1.73 – 1.58 (-, 16H, 8 × $-NCH_2C\underline{H_2}-$, 8 × $-C\underline{H}CH_3$), 1.58 – 1.48 (-, 8H, 8 × $-C\underline{H}(CH_3)_2$), 1.48 – 1.32 (-, 16H, 8 × $-C\underline{H_2}-$), 1.32 – 1.20 (-, 16H, 8 × $-C\underline{H_2}-$), 1.20 – 1.12 (-, 16H, 8 × $-C\underline{H_2}-$), 1.06 – 1.05 (-, 12H, 4 × $-CHC\underline{H_3}$), 1.04 (d, $^3J_{HH}$ = 6.7 Hz, 6H, 2 × $-CHC\underline{H_3}$), 1.03 (d, $^3J_{HH}$ = 6.5 Hz, 6H, 2 × $-CHC\underline{H_3}$), 0.88 – 0.85 (-, 48H, 8 × $-CH(C\underline{H_3})_2$).

^{13}C NMR (150.9 MHz, CD_2Cl_2, 295 K):

δ [ppm] = 173.6 (2 × quart.), 173.5 (2 × quart.), 172.5 (2 × quart.), 171.74 (2 × quart.), 171.67 (2 × quart.), 171.3 (2 × quart.), 168.0 (2 × quart.), 167.9 (2 × quart.), 167.1 (2 × quart.), 166.6 (2 × quart.), 166.5 (2 × quart.), 166.2 (2 × quart.), 143.8 (4 × quart.), 143.7 (2 × quart.), 143.0 (2 × quart.), 142.3 (2 × quart.), 142.0 (2 × quart.), 141.9 (2 × quart.), 141.8 (2 × quart.), 137.7 (4 × quart.), 137.4 (2 × quart.), 128.4 (2 × tert.), 127.4 (2 × tert.), 127.30 (2 × tert.), 127.25 (2 × tert.), 125.0 (2 × tert.), 122.6 (2 × tert.), 121.3 (2 × tert.), 121.2 (4 × tert.), 119.1 (8 × quart.), 110.9 (4 × tert.), 110.7 (2 × tert.), 110.6 (2 × tert.), 89.9 (4 × tert.), 89.6 (4 × tert.), 49.93 (4 × quart.), 49.92 (2 × quart.), 49.8 (2 × quart.), 43.5 (4 × sec.), 43.4 (4 × sec.), 40.8 (2 × quart.), 40.7 (2 × quart.), 39.5 (8 × sec.), 37.50 (6 × sec.), 37.48 (2 × sec.), 34.5 (4 × sec.), 34.4 (4 × sec.), 31.3 (8 × tert.), 28.41 (4 × tert.), 28.403 (2 × tert.), 28.396 (2 × tert.), 26.90 (2 × prim.), 26.86 (6 × prim.), 26.82 (2 × prim.), 26.81 (2 × prim.), 26.63 (2 × prim.), 26.59 (2 × prim.), 25.1 (6 × sec.), 25.0 (2 × sec.), 22.84 (6 × prim.), 22.82 (2 × prim.), 22.75 (6 × prim.), 22.73 (2 × prim.), 19.84 (6 × prim.), 19.78 (2 × prim.).

ESI-MS pos (high res.): [$M^{\cdot 2+}$]

calc.: 1446.99539

found: 1446.99522 Δ = 0.12 ppm

Synthesis of **SQ_4-$Bdan_2$**

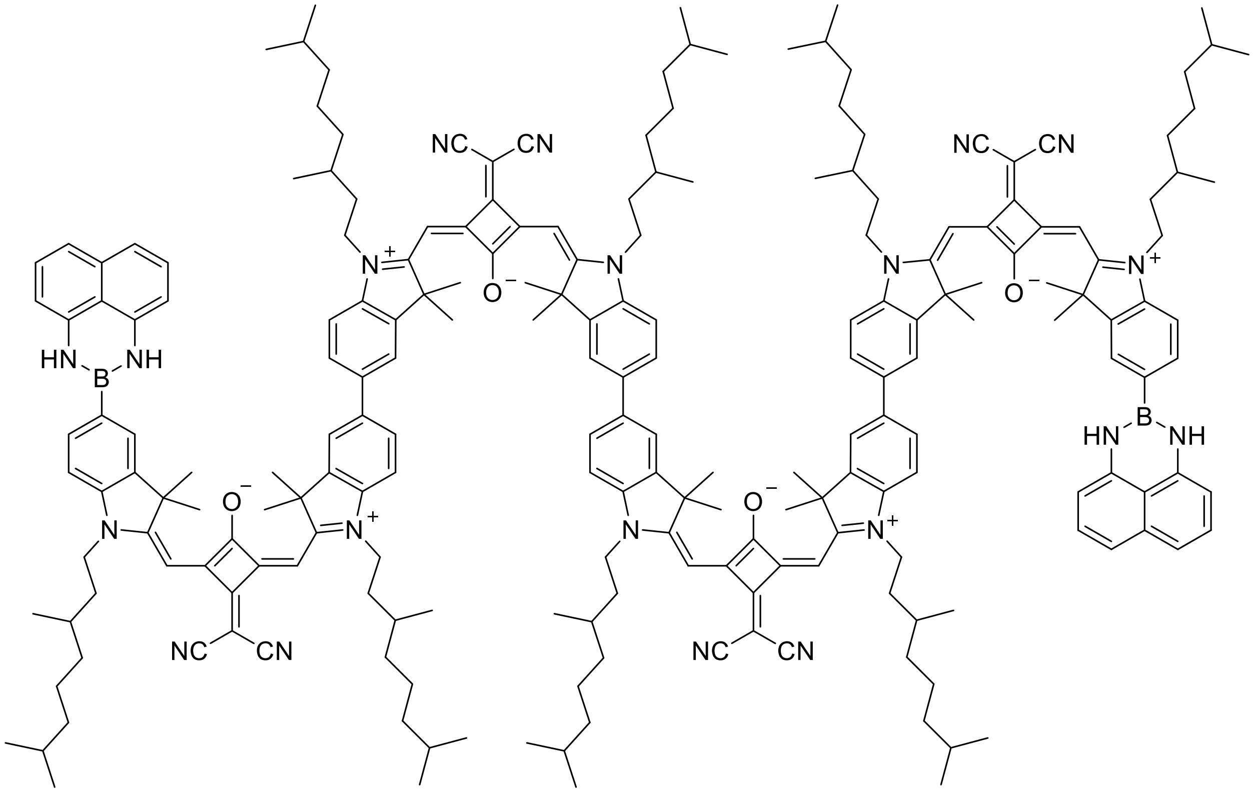

Synthesis following **GP I**:

SQ_2-$Bdan_2$ (382 mg, 215 µmol), 2 M HCl (2.58 mL, 5.15 mmol), THF (20 mL).

Synthesis following **GP II**:

SQ-BrBdan (458 mg, 472 µmol), XPhos-Pd-G2 (17.0 mg, 21.0 µmol), XPhos (20.0 mg, 43.0 µmol), Cs_2CO_3 (280 mg, 859 µmol), THF/water 4:1 (30 mL); flash column chromatography (eluent: DCM + 0.3 % MeOH), recycling HPLC (eluent: DCM + 0.2 % MeOH), precipitation in *n*-hexane.

Yield: 155 mg (48.0 µmol, 22 %) of a green solid.

$C_{216}H_{264}B_2N_{20}O_4$ [3226.24]

^{1}H NMR (600.1 MHz, CD_2Cl_2, 295 K):

δ [ppm] = 7.68 (dd, $^3J_{HH}$ = 8.0 Hz, $^4J_{HH}$ = 1.2 Hz, 2H, 2 × -CH-), 7.68 – 7.65 (-, 2H, 2 × -CH-), 7.65 – 7.58 (-, 12H, 12 × -CH-), 7.19 – 7.11 (-, 12H, 12 × -CH-), 7.03 (dd, $^3J_{HH}$ = 8.4 Hz, $^4J_{HH}$ = 0.9 Hz, 4H, 4 × -CH-), 6.56 – 6.52 (-, 6H, 6 × -CCHC-), 6.51 (s, 2H, 2 × -CH-), 6.49 (dd, $^3J_{HH}$ = 7.3 Hz, $^4J_{HH}$ = 0.92 Hz, 4H, 4 × -CH-), 6.20 (s, 4H, 4 × -NH), 4.19 – 3.98 (-, 16H, 8 × -NCH$_2$-), 1.93 – 1.75 (-, 32H,

8 × -$NCH_2C\underline{H}_2$-, 4 × -$C(C\underline{H}_3)_2$), 1.83 (s, 12H, 2 × -$C(C\underline{H}_3)_2$), 1.81 (s, 12H, 2 × -$C(C\underline{H}_3)_2$), 1.75 – 1.58 (-, 16H, 8 × -$NCH_2C\underline{H}_2$-, 8 × -$C\underline{H}CH_3$), 1.58 – 1.47 (-, 8H, 8 × -$C\underline{H}(CH_3)_2$), 1.47 – 1.11 (- 48H, 8 × -$C\underline{H}_2C\underline{H}_2C\underline{H}_2$-), 1.07 – 1.04 (-, 12H, 4 × -$CHC\underline{H}_3$), 1.04 (d, $^3J_{HH}$ = 6.2 Hz, 6H, 2 × -$CHC\underline{H}_3$), 1.03 (d, $^3J_{HH}$ = 6.4 Hz, 6H, 2 × -$CHC\underline{H}_3$), 0.89 – 0.86 (-, 24H, 4 × -$CH(C\underline{H}_3)_2$), 0.869 (d, $^3J_{HH}$ = 6.6 Hz, 12H, 2 × -$CH(C\underline{H}_3)_2$), 0.865 (d, $^3J_{HH}$ = 6.6 Hz, 12H, 2 × -$CH(C\underline{H}_3)_2$).

^{13}C NMR (150.9 MHz, CD_2Cl_2, 295 K):

δ [ppm] = 173.6 (2 × quart.), 173.5 (2 × quart.), 172.1 (2 × quart.), 171.7 (6 × quart.), 168.1 (4 × quart.), 167.0 (2 × quart.), 166.6 (2 × quart.), 166.5 (4 × quart.), 144.3 (2 × quart.), 143.81 (2 × quart.), 143.77 (4 × quart.), 142.7 (2 × quart.), 141.90 (2 × quart.), 141.89 (2 × quart.), 141.78 (2 × quart.), 141.5 (4 × quart.), 137.8 (2 × quart.), 137.7 (2 × quart.), 137.6 (2 × quart.), 136.7 (2 × quart.), 132.1 (2 × tert.), 128.0 (4 × tert.), 127.4 (4 × tert.), 127.3 (2 × tert.), 125.5 (2 × tert.), 121.22 (2 × tert.), 121.19 (4 × tert.), 120.1 (2 × quart.), 119.1 (6 × quart.), 119.0 (2 × quart.), 118.1 (4 × tert.), 111.0 (2 × tert.), 110.9 (4 × tert.), 110.3 (2 × tert.), 106.4 (4 × tert.), 90.0 (2 × tert.), 89.9 (4 × tert.), 89.8 (2 × tert.), 50.0 (2 × quart.), 49.93 (2 × quart.), 49.92 (2 × quart.), 49.7 (2 × quart.), 43.53 (2 × sec.), 43.49 (4 × sec.), 43.3 (2 × sec.), 40.9 (2 × quart.), 40.8 (2 × quart.), 39.5 (8 × sec.), 37.50 (6 × sec.), 37.48 (2 × sec.), 34.5 (6 × sec.), 34.4 (2 × sec.), 31.33 (6 × tert.), 31.30 (2 × tert.), 28.41 (4 × tert.), 28.40 (4 × tert.), 26.90 (2 × prim.), 26.86 (6 × prim.), 26.82 (6 × prim.), 26.77 (2 × prim.), 25.1 (4 × sec.), 25.0 (4 × sec.), 22.8 (8 × prim.), 22.7 (8 × prim.), 19.84 (4 × prim), 19.82 (2 × prim.), 19.81 (2 × prim.).

The boron-bound carbon was not found due to quadrupolar relaxation.

MALDI-MS pos (high res.): [$M^{\cdot+}$]

calc.: 3226.13361

found: 3226.12600

Synthesis of **SQ_6**

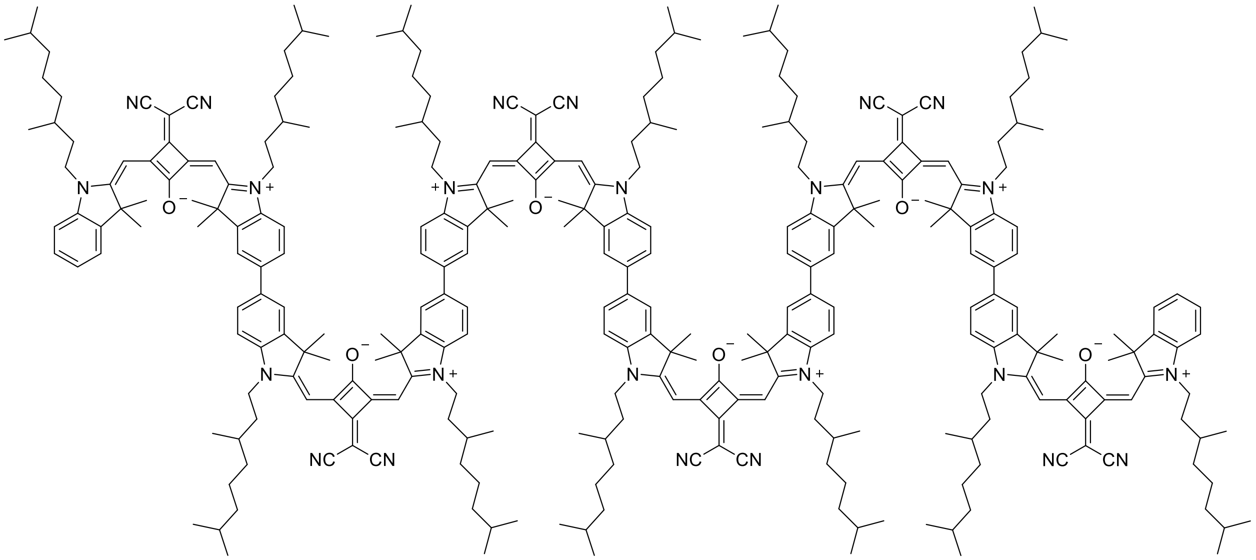

Synthesis following **GP I**:

SQ_4-$Bdan_2$ (100 mg, 31.0 µmol), 2 M HCl (372 µL, 745 µmol), THF (10 mL).

Synthesis following **GP II**:

SQ-Br (60.0 mg, 74.0 µmol), XPhos-Pd-G2 (2.44 mg, 3.10 µmol), XPhos (2.96 mg, 6.21 µmol), Cs_2CO_3 (40.0 mg, 124 µmol), THF/water 4:1 (10 mL); flash column chromatography (eluent: DCM + 0.5 → 0.75 % MeOH), recycling preparative GPC ($CHCl_3$), precipitation in *n*-hexane.
<u>Yield</u>: 32.0 mg (7.38 µmol, 24 %) of a green solid.

$C_{294}H_{374}N_{24}O_6$ [4340.39]

^{1}H NMR (600.1 MHz, CD_2Cl_2, 295 K):

δ [ppm] = 7.64 – 7.58 (-, 20H, 20 × -C$\underline{H}$-), 7.40 (dd, $^3J_{HH}$ = 7.3 Hz, $^4J_{HH}$ = 0.6 Hz, 2H, 2 × -C$\underline{H}$-), 7.36 (td, $^3J_{HH}$ = 7.8 Hz, $^4J_{HH}$ = 1.1 Hz, 2H, 2 × -C$\underline{H}$-), 7.21 (td, $^3J_{HH}$ = 7.4 Hz, $^4J_{HH}$ = 0.5 Hz, 2H, 2 × -C$\underline{H}$-), 7.19 – 7.12 (-, 10H, 10 × -C$\underline{H}$-), 7.09 (d, $^3J_{HH}$ = 8.0 Hz, 2H, 2 × -C$\underline{H}$-), 6.531 – 6.524 (-, 4H, 4 × -CC$\underline{H}$C-), 6.52 (s, 2H, 2 × -CC$\underline{H}$C-), 6.51 (s, 2H, 2 × -CC$\underline{H}$C-), 6.50 (s, 2H, 2 × -CC$\underline{H}$C-), 6.48 (s, 2H, 2 × -CC$\underline{H}$C-), 4.17 – 3.98 (-, 24H, 12 × -NC$\underline{H_2}$-), 1.92 – 1.73 (-, 60H, 12 × -NCH$_2$C$\underline{H_2}$, 8 × -C(C$\underline{H_3}$)$_2$), 1.81 (s, 12H, 2 × -C(C$\underline{H_3}$)$_2$), 1.76 (s, 12H, 2 × -C(C$\underline{H_3}$)$_2$), 1.73 – 1.58 (-, 24H, 12 × -NCH$_2$C$\underline{H_2}$, 12 × -C$\underline{H}$CH$_3$), 1.58 – 1.48 (-, 12H, 12 × -C$\underline{H}$(CH$_3$)$_2$), 1.46 – 1.32 (-, 24H, 12 × -C$\underline{H_2}$-), 1.32 – 1.20 (-, 24H,

12 × -C$\underline{H}_2$-), 1.20 – 1.13 (-, 24H, 12 × -C$\underline{H}_2$-), 1.07 – 1.01 (-, 36H, 12 × -CHC$\underline{H}_3$), 0.89 – 0.85 (-, 72H, 12 × -CH(C$\underline{H}_3$)$_2$).

^{13}C NMR (150.9 MHz, CD_2Cl_2, 295 K):

δ [ppm] = 173.55 (4 × quart.), 173.49 (2 × quart.), 172.4 (2 × quart.), 171.74 (2 × quart.), 171.70 (2 × quart.), 171.66 (2 × quart.), 171.61 (2 × quart.), 171.3 (2 × quart.), 168.03 (2 × quart.), 168.02 (2 × quart.), 167.9 (2 × quart.), 167.1 (2 × quart.), 166.6 (2 × quart.), 166.54 (2 × quart.), 166.50 (2 × quart.), 166.45 (2 × quart.), 166.2 (2 × quart.), 143.8 (8 × quart.), 143.7 (2 × quart.), 143.0 (2 × quart.), 142.3 (2 × quart.), 141.95 (2 × quart.), 141.89 (6 × quart.), 141.8 (2 × quart.), 137.70 (4 × quart.), 137.66 (4 × quart.), 137.4 (2 × quart.), 128.4 (2 × tert.), 127.36 (2 × tert.), 127.33 (4 × tert.), 127.31 (2 × tert.), 127.2 (2 × tert.), 125.0 (2 × tert.), 122.6 (2 × tert.), 121.22 (6 × tert.), 121.16 (4 × tert.), 119.13 (2 × quart.), 119.10 (10 × quart.), 110.91 (4 × tert.), 110.88 (2 × tert.), 110.85 (2 × tert.), 110.7 (2 × tert.), 110.6 (2 × tert.), 89.9 (6 × tert.), 89.8 (2 × tert.), 89.6 (4 × tert.), 49.93 (6 × quart.), 49.91 (2 × quart.), 49.90 (2 × quart.), 49.8 (2 × quart.), 43.50 (8 × sec.), 43.39 (2 × sec.), 43.37 (2 × sec.), 40.81 (2 × quart.), 40.79 (2 × quart.), 40.7 (2 × quart.), 39.5 (12 × sec.), 37.50 (10 × sec.), 37.47 (2 × sec.), 34.5 (8 × sec.), 34.40 (2 × sec.), 34.39 (2 × sec.), 31.3 (12 × tert.), 28.41 (10 × tert.), 28.39 (2 × tert.), 26.90 (2 × prim.), 26.86 (10 × prim.), 26.81 (8 × prim.), 26.63 (2 × prim.), 26.59 (2 × prim.), 25.1 (10 × sec.), 25.0 (2 × sec.), 22.84 (10 × prim.), 22.82 (2 × prim.), 22.75 (10 × prim.), 22.73 (2 × prim.), 19.83 (10 × prim.), 19.78 (2 × prim.).

ESI-MS pos (high res.): [$M^{\cdot 2+}$] + 2 Na^+

calc.: 2192.9790

found: 2192.9789 Δ = 0.03 ppm

Synthesis of **SQ_6-$Bdan_2$**

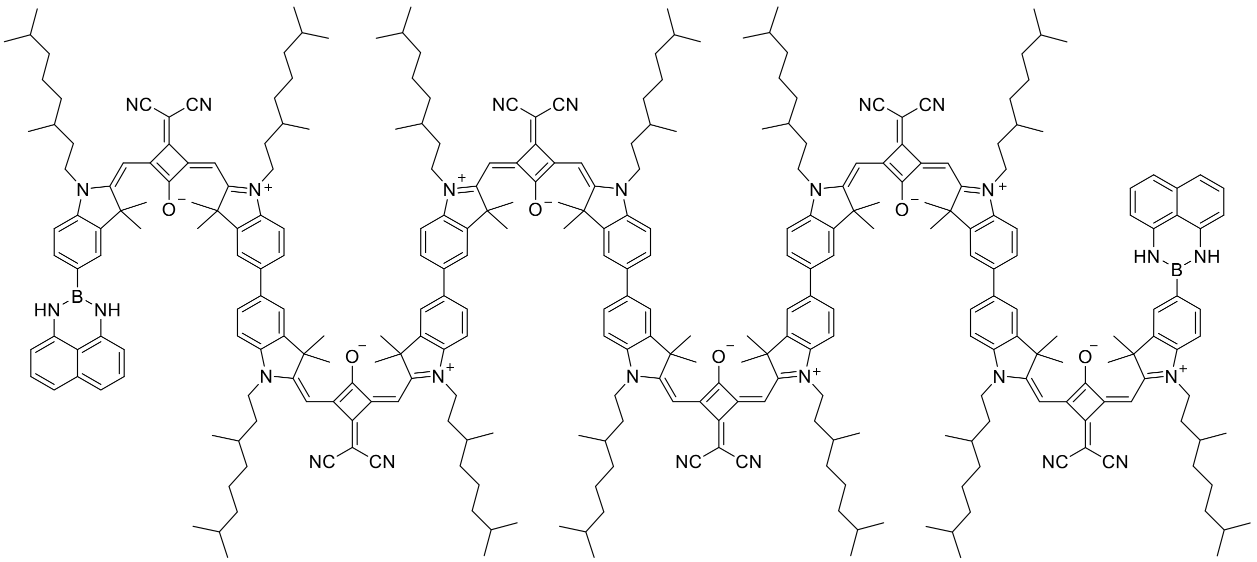

Synthesis following **GP I**:

SQ_4-$Bdan_2$ (140 mg, 43.0 µmol), 2 M HCl (521 µL, 1.04 mmol), THF (5 mL).

Synthesis following **GP II**:

SQ-BrBdan (101 mg, 104 µmol), XPhos-Pd-G2 (3.42 mg, 4.34 µmol), XPhos (4.14 mg, 8.69 µmol), Cs_2CO_3 (57.0 mg, 174 µmol), THF/water 4:1 (20 mL); flash column chromatography (eluent: DCM + 0.5 → 0.6 % MeOH), precipitation in *n*-hexane.
Yield: 155 mg (33.0 µmol, 76 %) of a green solid.

$C_{314}H_{388}B_2N_{28}O_6$ [4672.37]

^{1}H NMR (600.1 MHz, CD_2Cl_2, 295 K):

δ [ppm] = 7.71 – 7.66 (-, 4H, 4 × -CH-), 7.66 – 7.57 (-, 20H, 20 × -CH-), 7.19 – 7.12 (-,16H, 16 × -CH-), 7.03 (dd, $^3J_{HH}$ = 8.4 Hz, $^4J_{HH}$ = 0.7 Hz, 4H, 4 × -CH-), 6.55 – 6.50 (-, 12H, 12 × -CCHC-), 6.49 (dd, $^3J_{HH}$ = 7.3 Hz, $^4J_{HH}$ = 0.7 Hz, 4H, 4 × -CH-), 6.23 (s, 4H, 4 × -NH), 4.18 – 3.98 (-, 24H, 12 × -NCH_2-), 1.90 – 1.74 (-, 60H, 12 × -NCH_2CH_2, 8 × -$C(CH_3)_2$), 1.82 (s, 12H, 2 × -$C(CH_3)_2$), 1.81 (s, 12H, 2 × -$C(CH_3)_2$), 1.74 – 1.58 (-, 24H, 12 × -NCH_2CH_2, 12 × -$CHCH_3$), 1.58 – 1.48 (-, 12H, 12 × -$CH(CH_3)_2$), 1.46 – 1.32 (-, 24H, 12 × -CH_2-), 1.32 – 1.20 (-, 24H, 12 × -CH_2-), 1.20 – 1.12 (-, 24H, 12 × -CH_2-), 1.08 – 1.02 (-, 36H, 12 × -$CHCH_3$), 0.89 – 0.85 (-, 72H, 12 × -$CH(CH_3)_2$).

^{13}C NMR (150.9 MHz, CD_2Cl_2, 295 K):

δ [ppm] = 173.55 (4 × quart.), 173.47 (2 × quart.), 172.0 (2 × quart.), 171.72 (2 × quart.), 171.69 (2 × quart.), 171.67 (6 × quart.), 168.1 (2 × quart.), 168.0 (4 × quart.), 167.0 (2 × quart.), 166.6 (2 × quart.), 166.53 (2 × quart.), 166.50 (2 × quart.), 166.48 (4 × quart.), 144.3 (2 × quart.), 143.83 (2 × quart.), 143.77 (2 × quart.), 143.75 (4 × quart.), 143.72 (2 × quart.), 142.7 (2 × quart.), 141.90 (2 × quart.), 141.88 (2 × quart.), 141.87 (2 × quart.), 141.85 (2 × quart.), 141.79 (2 × quart.), 141.5 (4 × quart.), 137.8 (2 × quart.), 137.7 (6 × quart.), 137.6 (2 × quart.), 136.7 (2 × quart.), 132.1 (2 × tert.), 128.0 (4 × tert.), 127.3 (8 × tert.), 125.5 (2 × tert.), 121.2 (12 × tert.), 120.1 (2 × quart.), 119.1 (10 × quart.), 119.0 (2 × quart.), 118.1 (4 × tert.), 111.0 (2 × tert.), 110.92 (4 × tert.), 110.88 (4 × tert.), 110.3 (2 × tert.), 106.4 (4 × tert.), 90.0 (2 × tert.), 89.85 (8 × tert.), 89.80 (2 × tert.), 50.0 (2 × quart.), 49.9 (8 × quart.), 49.7 (2 × quart.), 43.53 (2 × sec.), 43.49 (8 × sec.), 43.3 (2 × sec.), 40.9 (2 × quart.), 40.8 (4 × quart.), 39.5 (12 × sec.), 37.5 (12 × sec.), 34.5 (6 × sec.), 34.45 (4 × sec.), 34.40 (2 × sec.), 31.3 (12 × tert.), 28.4 (12 × tert.), 26.90 (2 × prim.), 26.86 (10 × prim.), 26.82 (10 × prim.), 26.78 (2 × prim.), 25.0 (12 × sec.), 22.84 (12 × prim.), 22.75 (12 × prim.), 19.84 (10 × prim.), 19.81 (2 × prim.).

The boron-bound carbon was not found due to quadrupolar relaxation.

MALDI-MS pos (high res.): [$M^{\cdot +}$]

calc.: 4672.122

found: 4672.101

Synthesis of **SQ_8**

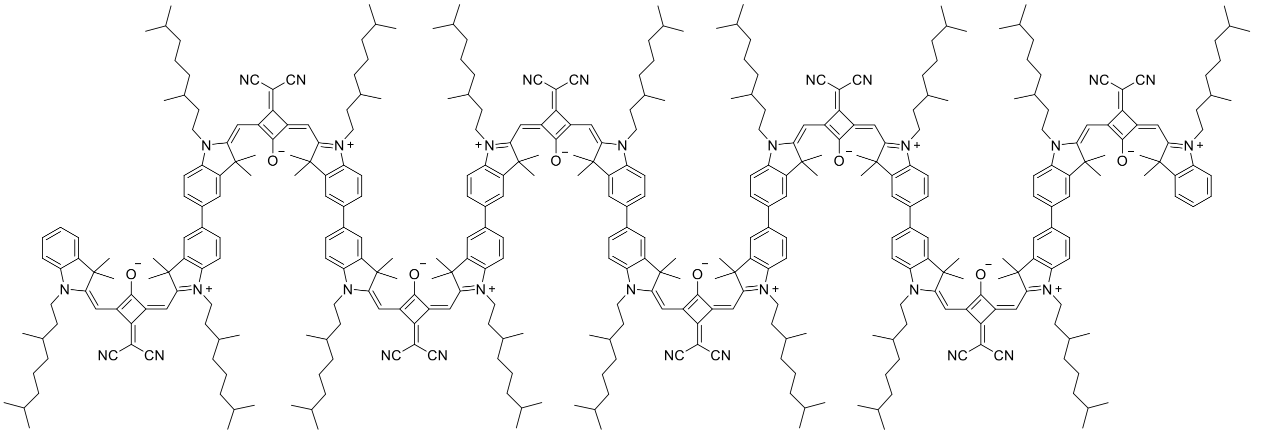

Synthesis following **GP I**:

SQ_6-$Bdan_2$ (50.0 mg, 10.7 µmol), 2 M HCl (129 µL, 257 µmol), THF (2 mL).

Synthesis following **GP II**:

SQ-Br (21.0 mg, 26.0 µmol), XPhos-Pd-G2 (8.43 µg, 1.07 µmol), XPhos (1.02 mg, 2.14 µmol), Cs_2CO_3 (14.0 mg, 43.0 µmol), THF/water 4:1 (5 mL); flash column chromatography (eluent: DCM + 0.5 → 0.7 % MeOH), recycling HPLC (eluent: DCM + 0.2 % MeOH), precipitation in *n*-hexane.

Yield: 26.0 mg (4.50 µmol, 42 %) of a green solid.

$C_{392}H_{498}N_{32}O_8$ [5786.51]

^{1}H NMR (600.1 MHz, CD_2Cl_2, 295 K):

δ [ppm] = 7.64 – 7.58 (-, 28H, 28 × -C$\underline{H}$-), 7.40 (d, $^3J_{HH}$ = 7.4 Hz, 2H, 2 × -C$\underline{H}$-), 7.36 (t, $^3J_{HH}$ = 7.7 Hz, 2H, 2 × -C$\underline{H}$-), 7.22 (t, $^3J_{HH}$ = 7.4 Hz, 2H, 2 × -C$\underline{H}$-), 7.19 – 7.11 (-, 14H, 14 × -C$\underline{H}$-), 7.09 (d, $^3J_{HH}$ = 8.0 Hz, 2H, 2 × -C$\underline{H}$-), 6.538 – 6.524 (-, 4H, 4 × -CC$\underline{H}$C-), 6.521 (s, 2H, 2 × -CC$\underline{H}$C-), 6.520 – 6.505 (-, 6H, 6 × -CC$\underline{H}$C-), 6.50 (s, 2H, 2 × -CC$\underline{H}$C-), 6.48 (s, 2H, 2 × -CC$\underline{H}$C-), 4.16 – 3.99 (-, 32H, 32 × -NC$\underline{H_2}$-), 1.96 – 1.73 (-, 100H, 16 × -$NCH_2C\underline{H_2}$, 14 × -C(C$\underline{H_3}$)$_2$), 1.76 (s, 12H, 2 × -C(C$\underline{H_3}$)$_2$), 1.73 – 1.58 (-, 32H, 16 × -$NCH_2C\underline{H_2}$, 16 × -C$\underline{H}CH_3$), 1.58 – 1.48 (-, 16H, 12 × -C$\underline{H}(CH_3)_2$), 1.48 – 1.32 (-, 32H, 16 × -C$\underline{H_2}$-), 1.32 – 1.20 (-, 32H, 16 × -C$\underline{H_2}$-), 1.20 – 1.11 (-, 32H, 16 × -C$\underline{H_2}$-), 1.08 – 0.99 (-, 48H, 16 × -CHC$\underline{H_3}$), 0.89 – 0.84 (-, 96H, 16 × -CH(C$\underline{H_3}$)$_2$).

^{13}C NMR (150.9 MHz, CD_2Cl_2, 295 K):

δ [ppm] = 173.55 (6 × quart.), 173.49 (2 × quart.), 172.5 (2 × quart.), 171.75 (2 × quart.), 171.71 (2 × quart.), 171.66 (6 × quart.), 171.61 (2 × quart.), 171.3 (2 × quart.), 168.0 (6 × quart.), 167.9 (2 × quart.), 167.1 (2 × quart.), 166.57 (2 × quart.), 166.54 (2 × quart.), 166.50 (6 × quart.), 166.45 (2 × quart.), 166.2 (2 × quart.), 143.8 (12 × quart.), 143.7 (2 × quart.), 143.0 (2 × quart.), 142.3 (2 × quart.), 141.95 (2 × quart.), 141.88 (10 × quart.), 141.83 (2 × quart.), 137.71 (4 × quart.), 137.65 (8 × quart.), 137.4 (2 × quart.), 128.4 (2 × tert.), 127.3 (12 × tert.), 127.2 (2 × tert.), 125.0 (2 × tert.), 122.6 (2 × tert.), 121.21 (8 × tert.), 121.2 (6 × tert.), 119.1 (16 × quart.), 110.9 (12 × tert.), 110.7 (2 × tert.), 110.6 (2 × tert.), 89.9 (12 × tert.), 89.6 (4 × tert.), 49.93 (6 × quart.), 49.91 (6 × quart.), 49.90 (2 × quart.), 49.8 (2 × quart.), 43.5 (12 × sec.), 43.39 (2 × sec.), 43.37 (2 × sec.), 40.83 (2 × quart.), 40.81 (2 × quart.), 40.80 (2 × quart.), 40.7 (2 × quart.), 39.5 (16 × sec.), 37.50 (14 × sec.), 37.47 (2 × sec.), 34.45 (12 × sec.), 34.41 (2 × sec.), 34.39 (2 × sec.), 31.3 (16 × tert.), 28.4 (16 × tert.), 26.90 (2 × prim.), 26.86 (14 × prim.), 26.82 (12 × prim.), 26.63 (2 × prim.), 26.59 (2 × prim.), 25.1 (14 × sec.), 25.0 (2 × sec.), 22.84 (14 × prim.), 22.82 (2 × prim.), 22.75 (14 × prim.), 22.74 (2 × prim.), 19.84 (14 × prim.), 19.79 (2 × prim.).

ESI-MS pos (high res.): $[M^{\cdot\cdot\cdot\cdot\cdot\cdot 6+}]$

calc.: 963.65854

found: 963.65768 Δ = 0.90 ppm

3 Absorption Spectroscopic Data of Squaraine Oligomers

Steady-State Absorption Spectroscopy

- Jasco V670 UV/vis/NIR spectrophotometer (software: SpectraManager v.2.08.04)
- Agilent Technologies Cary 5000 UV/vis/NIR spectrophotometer (software: Agilent Cary WinUV Analysis and Bio v.4.2)

Spectroscopic grade solvents were used as received (Acros Organics, Supelco, VWR). Absorption measurements were carried out in silylated quartz cuvettes from Starna (10 mm, Pfungstadt, Germany) at rt. Silylation procedure was carried out according to literature.[6] Aggregation of oligomers could be excluded by measuring a series of samples with different concentrations (10^{-7} – 10^{-5}). Variable temperature absorption experiments were performed with an external cryostat OptistatDN (Oxford Instruments Nanoscience, England). The cryostat was placed into the beam path of the spectrophotometer (Cary 5000). The temperature was manually regulated with an electronic temperature control unit while the sample is being stirred. Stirring was stopped while measuring the spectra. For sub rt temperatures the measuring cell was purged with argon while inserting the sample and sealed with a cuff to exclude moisture. Transition dipole moments were calculated by eq. (S2):

$$\mu_{\mathrm{eg}}^2 = \frac{3hc\varepsilon_0 \ln(10)\, 9n}{2000\pi^2 N_{Av}(n^2+2)^2} \int \frac{\varepsilon(\tilde{\mathrm{v}})\mathrm{d}\tilde{\mathrm{v}}}{\tilde{\mathrm{v}}} \qquad \text{S2}$$

where h is the Planck's constant, c is the speed of light (3×10^{10} cm s^{-1}), ε_0 is the electric field constant, n is the refractive index of the solvent, N_{Av} is the Avogadro constant and $\varepsilon(\tilde{v})$ is the extinction coefficient as a function of the wavenumer $\tilde{v}$.

Table S1. Spectroscopic data of all squaraine oligomers in selected solvents at rt.

Compound	Solvent	$\tilde{\nu}_{abs}$ / cm^{-1} (nm)	ε_{max} / 10^5 M^{-1}cm^{-1}	μ^2_{eg} / D^2
**SQ**	toluene	14300 (700)	2.11	97.7
	$CHCl_3$	14600 (687)	2.03	103
	THF	14500 (691)	2.10	109
	acetone	14700 (681)	2.04	115
	PhCN	14500 (690)	1.83	88.4
	DMF	14700 (682)	1.95	104
**SQ_2**	toluene	13400 (748)	3.53	218
	$CHCl_3$	13600 (735)	3.35	233
	THF	13500 (739)	3.24	238
	acetone	13700 (732)	3.22	260
	PhCN	13400 (744)	2.85	214
	DMF	13600 (736)	2.79	236
**SQ_3**	toluene	12900 (773)	4.05	350
	$CHCl_3$	13100 (761)	3.78	363
	THF	13100 (765)	3.73	387
	acetone	13200 (759)	2.91	336
	PhCN	13000 (772)	3.01	339
	DMF	13100 (763)	2.77	341
**SQ_4**	toluene	12700 (785)	5.30	504
	$CHCl_3$	12900 (773)	4.69	480
	THF	12900 (778)	4.45	508
	acetone	13000 (771)	3.76	536
	PhCN	12700 (785)	3.23	407
	DMF	12900 (777)	3.59	566
**SQ_5**	toluene	12600 (793)	5.75	601
	$CHCl_3$	12800 (780)	5.99	636
	THF	12800 (784)	5.46	659
	acetone	15000 (666)	4.19	680
	PhCN	12600 (792)	4.22	587
	DMF	15000 (668)	3.98	648
**SQ_6**	toluene	12600 (796)	5.89	682
	$CHCl_3$	12800 (784)	6.76	738
	THF	12700 (788)	6.18	780
	acetone	15300 (656)	5.81	757
	PhCN	13000 (772)	4.20	664
	DMF	15200 (659)	5.34	727
**SQ_7**	toluene	12600 (796)	6.82	830
	$CHCl_3$	12700 (785)	8.35	912
	THF	12700 (790)	7.59	961
	acetone	15400 (648)	7.23	906
	PhCN	14900 (672)	5.20	824
	DMF	15400 (651)	6.64	868
**SQ_8**	toluene	12700 (787)	7.56	953
	$CHCl_3$	12700 (785)	9.49	1037
	THF	12700 (789)	8.41	1054
	acetone	15500 (644)	8.23	995
	PhCN	15000 (666)	6.27	903
	DMF	15500 (646)	8.04	969
**SQ_9**	toluene	12700 (789)	8.38	1079
	$CHCl_3$	12700 (785)	10.8	1158
	THF	12700 (787)	10.0	1237
	acetone	15500 (644)	9.95	1123
	PhCN	15100 (661)	7.61	1014
	DMF	15500 (645)	9.26	1071

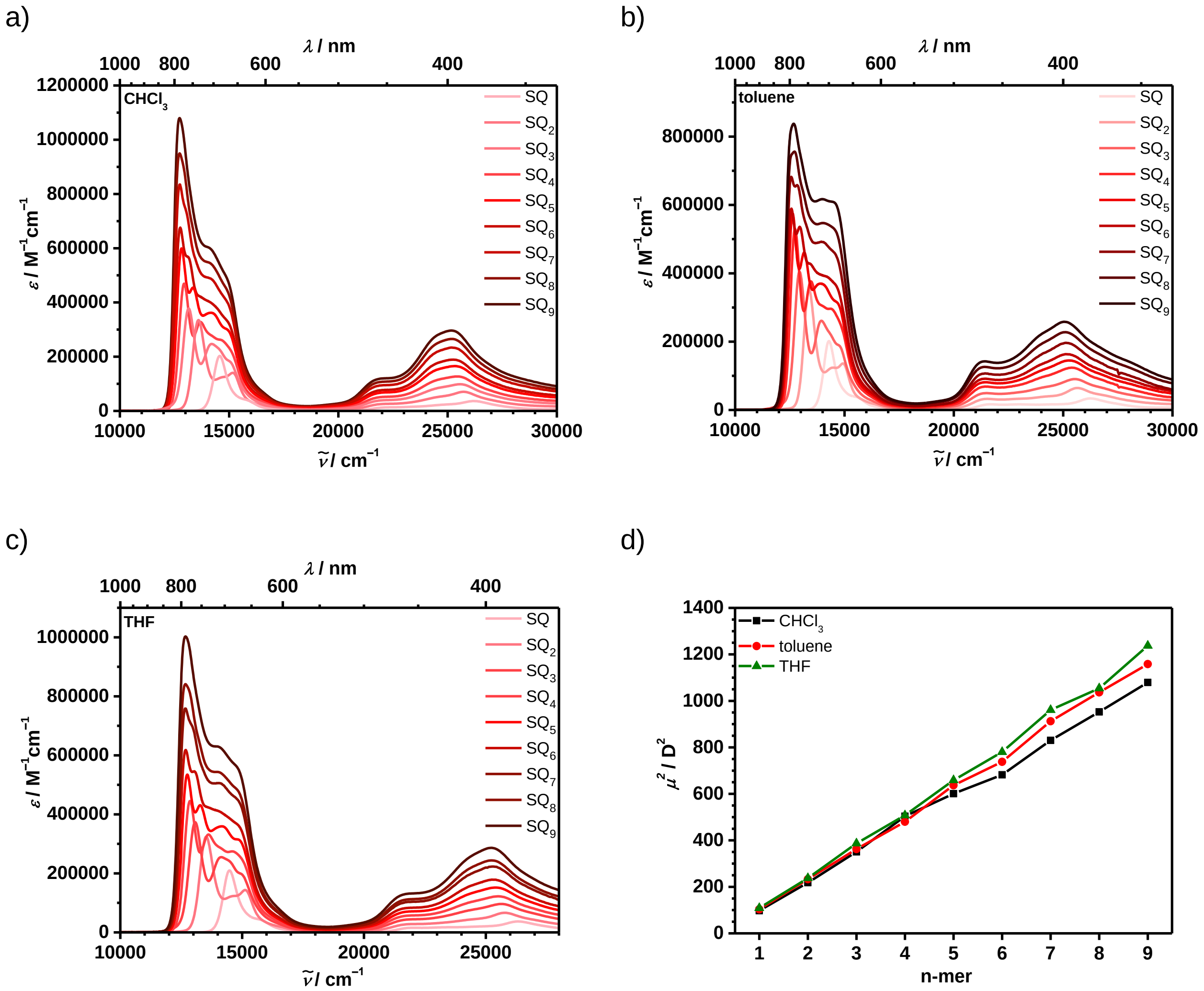

Figure S1. a)-c) Absorption spectra of **SQ** oligomers in selected solvents where J-type behavior is observed. d) Correlation of squared transition dipole moments of the exciton manifold vs oligomer length. Concentrations of about 4×10^{-8} M – 2×10^{-6} M were used. No concentration dependencies could be observed.

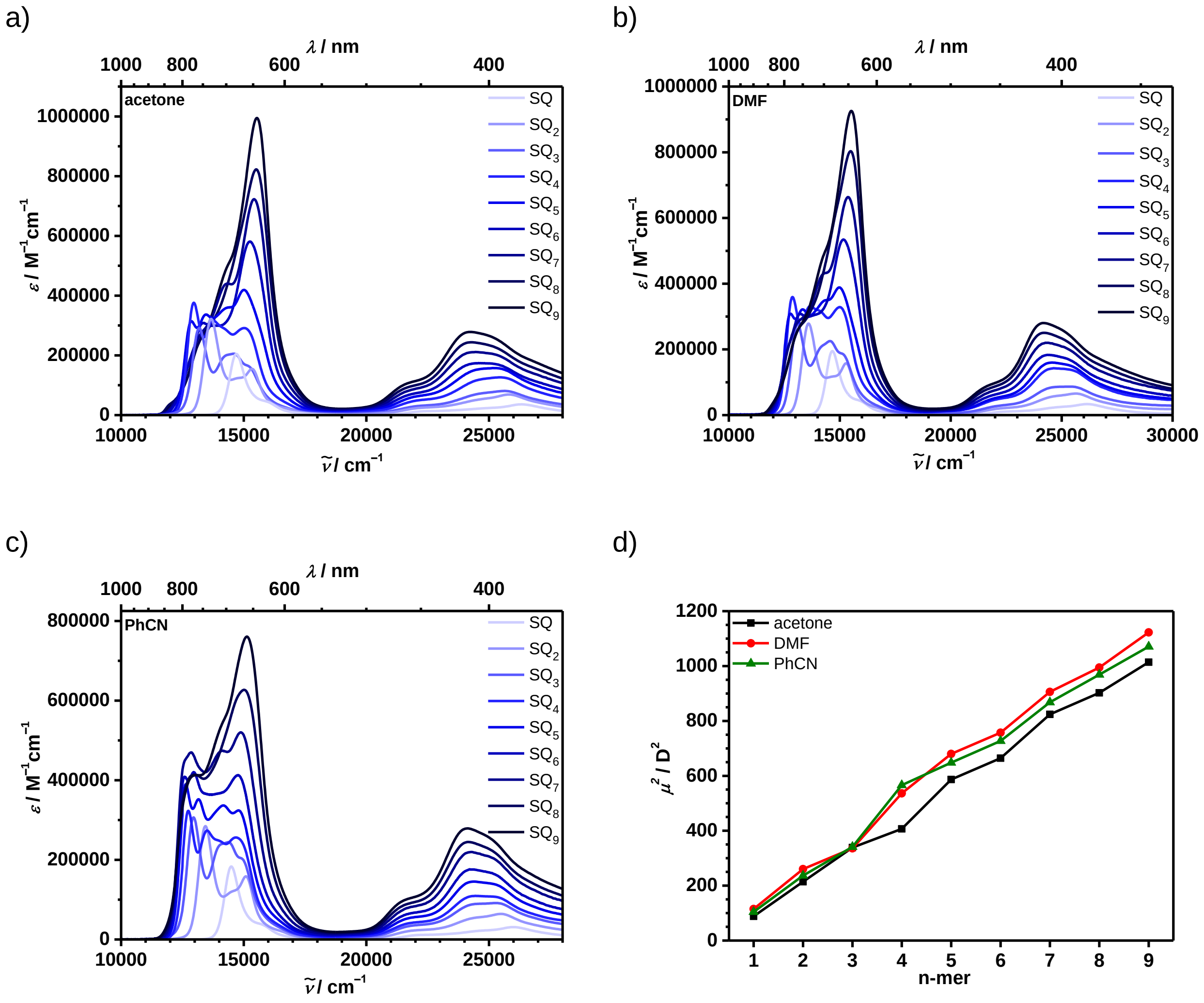

Figure S2. a)-c) Absorption spectra of **SQ** oligomers in selected solvents where H-type behavior is observed. d) Correlation of squared transition dipole moments of the exciton manifold vs oligomer length. Concentrations of about 4×10^{-8} M – 2×10^{-6} M were used. No concentration dependencies could be observed.

Table S2. Comparison of physical parameters of PhCN and $PhNO_2$.

	$PhNO_2$	PhCN
**m.p.**	5.8	-12.7
**b.p.**	210.9	191.1
μ **/ D**	14.0	13.9
ε	34.78	25.20
n	1.5562	1.5282
η **/mPa s**	1.6190	1.148
E_T^N	0.324	0.333
$f(n^2)$**-f(**ε**)**[a]	0.53	0.52
**Hildebrandt**		
δ_H **/Mpa**$^{1/2}$	22.6	22.7
**Hansen parameter**		
δ_D	20.0	17.4
δ_P	8.6	9.0
δ_H	4.1	3.3

a) $f(n^2) = (n^2 - 1)/(n^2 + 1)$ and $f(\varepsilon) = (\varepsilon - 1)/(\varepsilon + 1)$

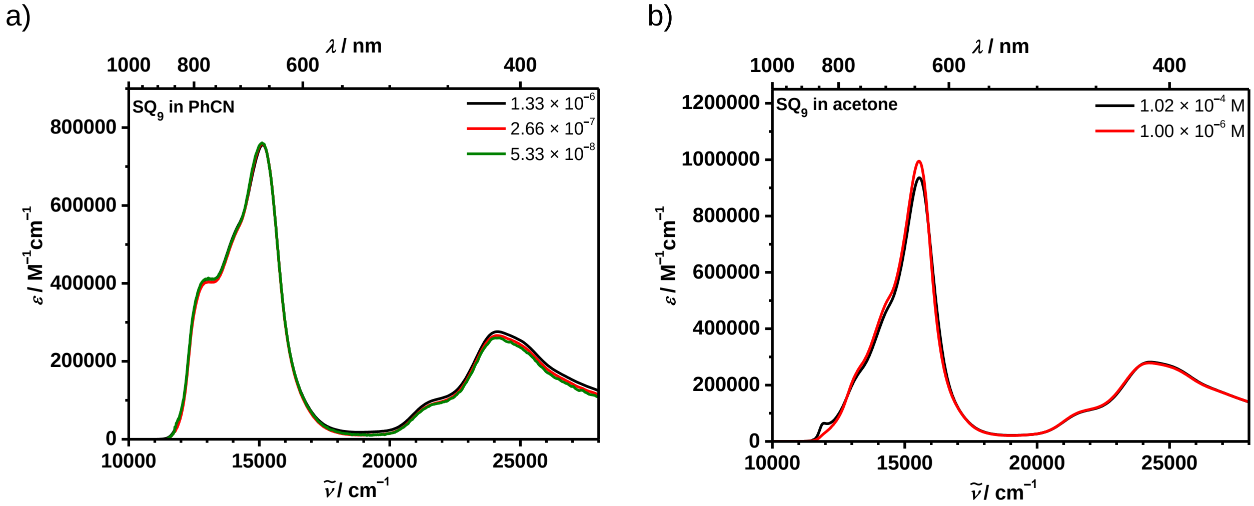

Figure S3. Proof of concentration independent absorption spectra of $\mathbf{SQ_9}$ in (a) PhCN and (b) acetone. The minor difference in extinction coefficient in acetone are due to an imperfect alignment of the 200 µm cuvette in the spectrometer.

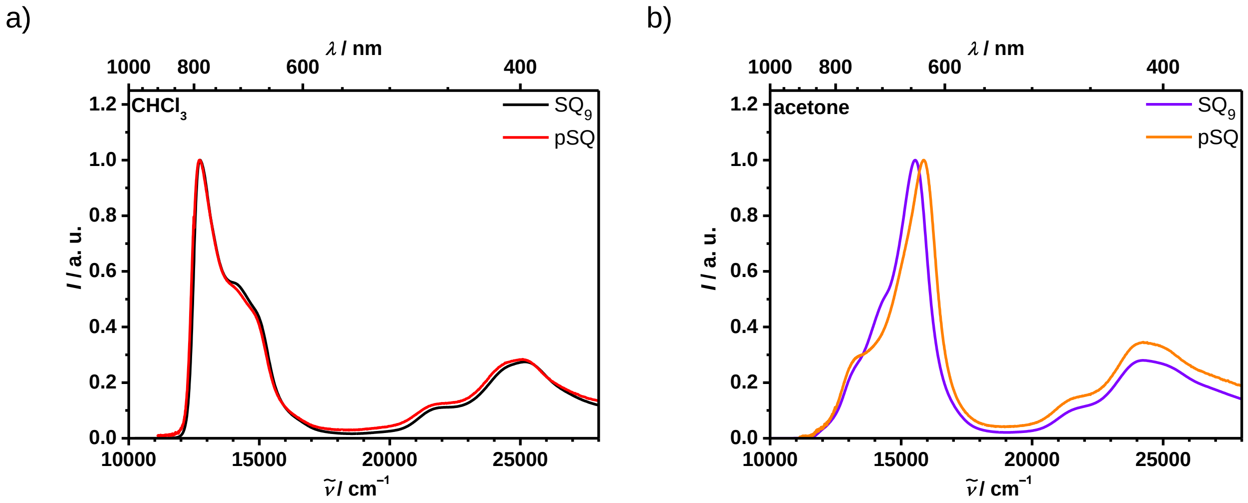

Figure S4. Comparison of absorption spectra of **SQ₉** and **SQ-polymer** in (a) $CHCl_3$ and in (b) acetone.[7]

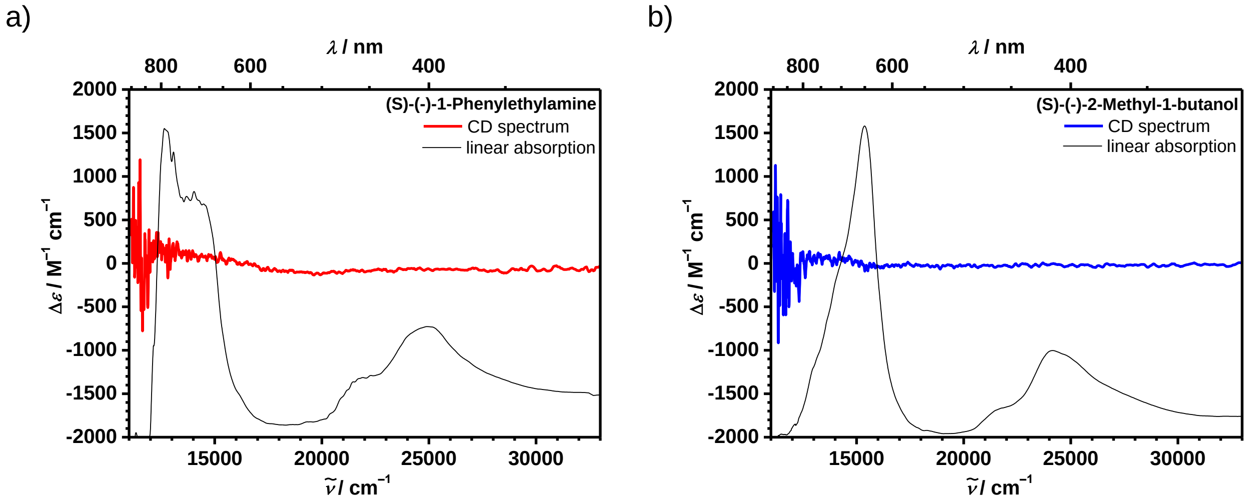

Figure S5. CD and normalised linear absorption spectra of **SQ₉** in (a) (S)-(-)-1-phenylethylamine (J-type, δ_D = 17.5) and (b) (S)-(-)-2-methyl-1-butanol (H-type, δ_D = 16.0). Scanning speed: 200 nm/min, dwell: 1 s, slid width: 2 nm, pitch: 0.5 nm.

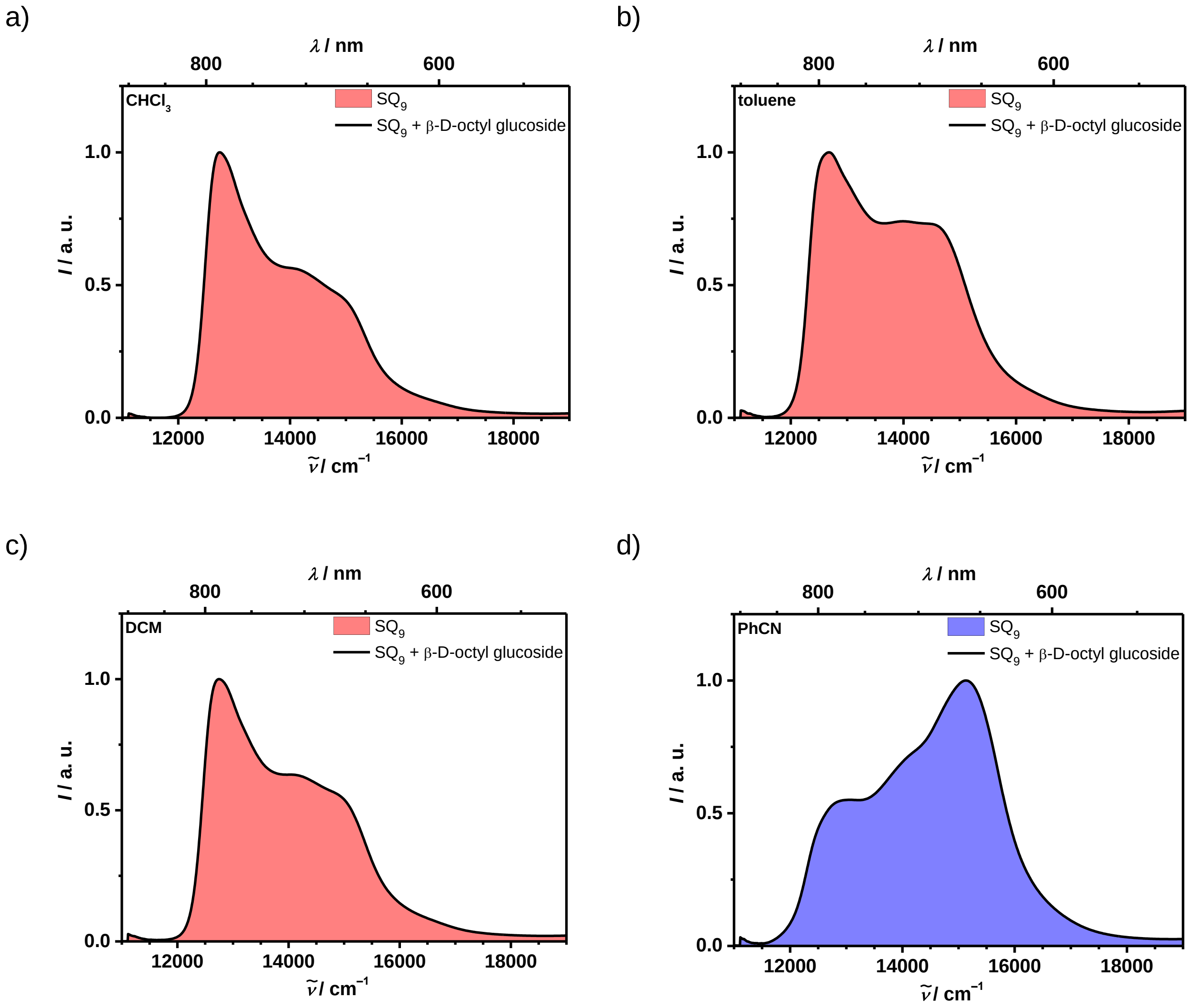

Figure S6. Absorption of pure **SQ$_9$** and **SQ$_9$** with added β-D-octyl glucoside in (a) $CHCl_3$, (b) toluene and (c) DCM and (d) PhCN. No change could be observed up to glucoside concentrations of 1.71 × 10^{-3} M.

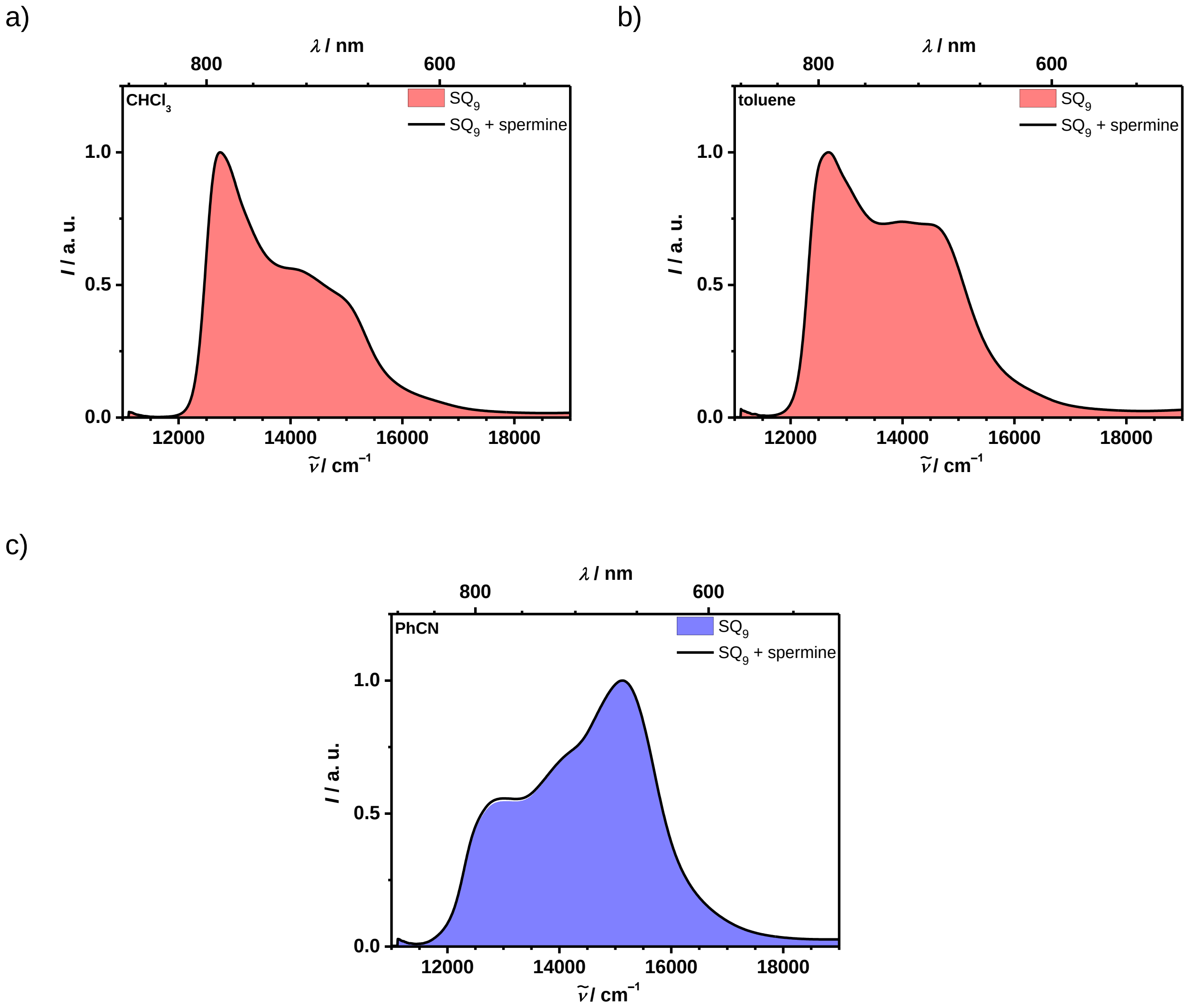

Figure S7. Absorption of pure **SQ_9** and **SQ_9** with added spermine in (a) $CHCl_3$, (b) toluene and (c) PhCN. No change could be observed up to spermine concentrations of 2.47×10^{-3} M.

3.1 Temperature Dependent Absorption Spectroscopy

Below the temperature dependent UV/Vis spectra (Figure S8) are given. The relative temperature dependency increases as the oligomer length increases and reaches a maximum at **SQ_9** (Figure S8f). The spectra were recorded with the above mentioned spectrometer/cryostat set-up. The Samples were first cooled and then heated up in 10 K steps. The sample were equilibrated for 30 min prior measuring. Each spectrum was density and baseline corrected and normalized to 0 at 10000 cm^{-1} (1000 nm). Detector and grating changeover were set to 870 nm, 1 nm steps with a scan rate of 400 nm min^{-1}. **SQ – SQ_3** were excluded since they show little or no temperature dependent behaviour.

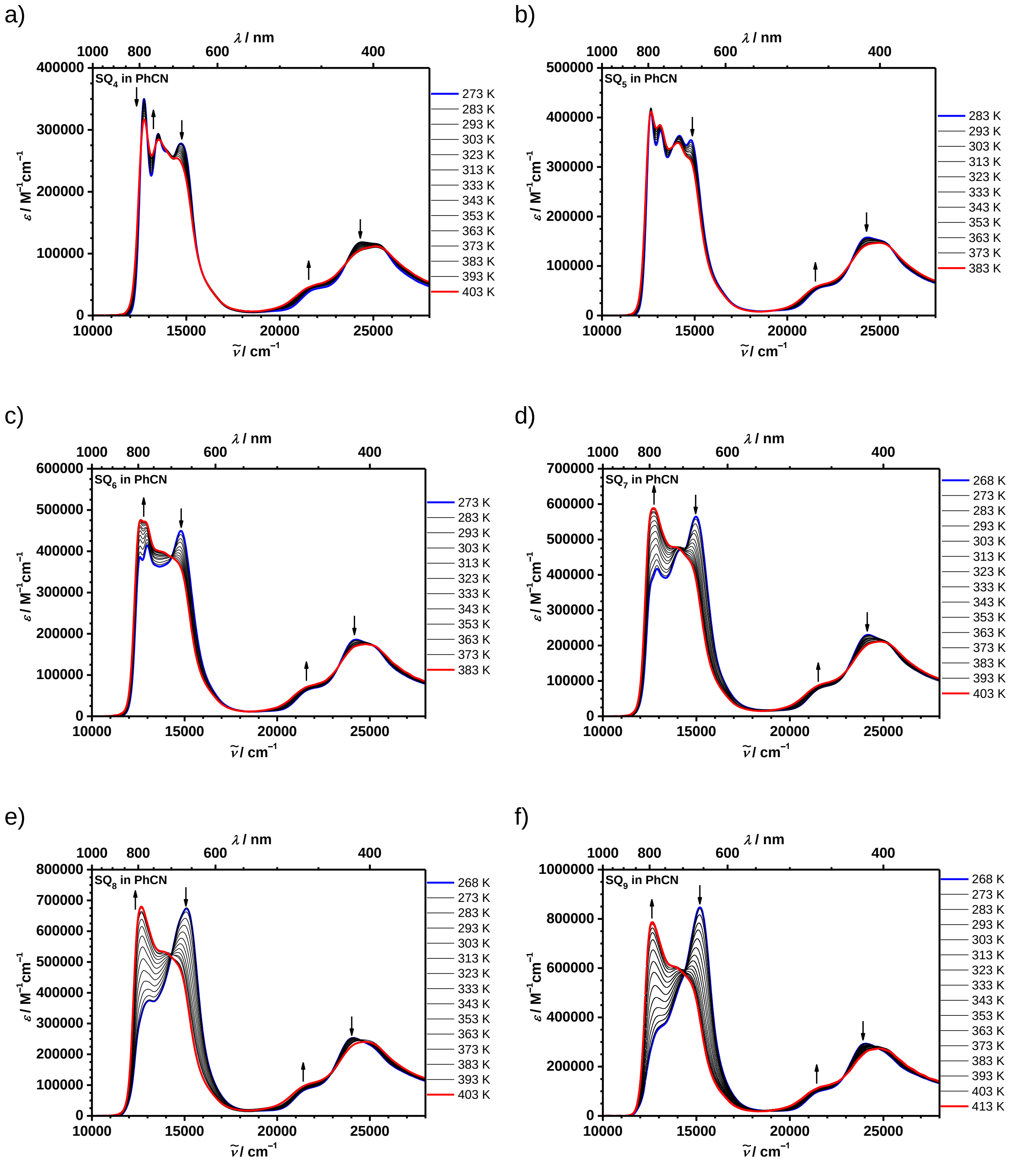

Figure S8. Temperature dependent absorption spectra of a) **SQ$_4$** (1.58×10^{-6} M), b) **SQ$_5$** (1.60×10^{-6} M), c) **SQ$_6$** (1.33×10^{-6} M), d) **SQ$_7$** (1.29×10^{-6} M), e) **SQ$_8$** (9.71×10^{-7} M) and f) **SQ$_9$** (1.07×10^{-6} M) in PhCN.

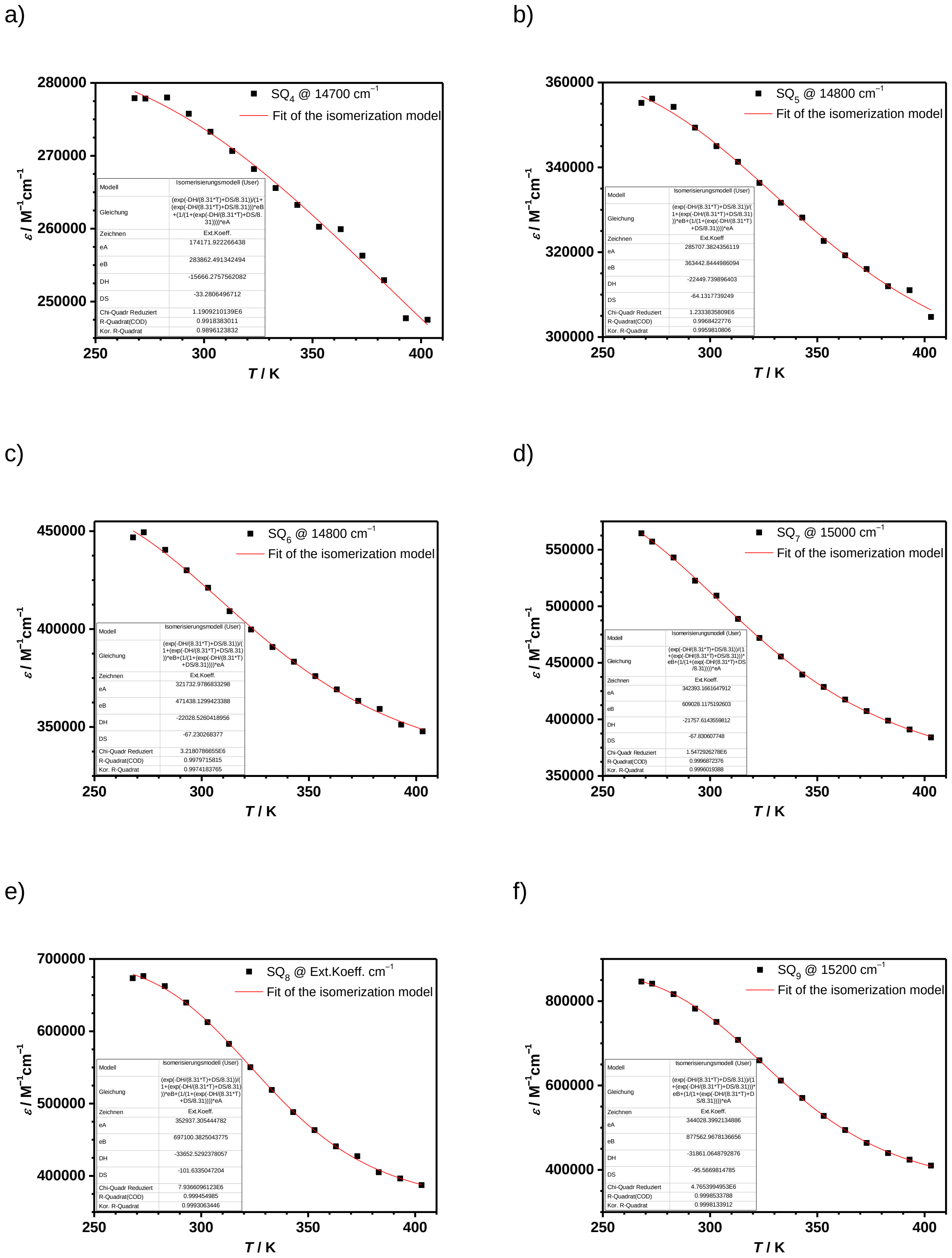

Figure S9. Fit of the temperature dependent extinction coefficient of **SQ⁴** to **SQ_9** in PhCN to Equation 1 in the main text. The resulting fit values are given in Table S3.

Table S3. Enthalpy and entropy for the coil-helix transformation determined by the fit of Equation 1 (main text) to the data points in Figure S9.

	ΔH / kJ mol^{-1}	ΔS / J mol^{-1} K^{-1}
**SQ$_4$**	−15.7	−33.3
**SQ$_5$**	−22.5	−64.1
**SQ$_6$**	−22.0	−67.2
**SQ$_7$**	−21.8	−67.8
**SQ$_8$**	−33.7	−101.6
**SQ$_9$**	−31.9	−95.6

3.2 Absorption Spectroscopy in Solvent Mixtures

To elucidate the Gibbs free energies of **SQ$_9$** in pure $CHCl_3$ and acetone, we measured absorption spectra in a series of solvent mixtures with known %vol. These mixtures were prepared by mixing stock solutions with known concentration. Here, we determined the %vol of the mixtures by weighing the stock solutions and recalculating the %vol using the density. Since mixing acetone and $CHCl_3$ is exothermic it was necessary to thermalise the solvent mixtures before measuring the absorption spectra (Figure S10. *Absorption spectra of **SQ9** in acetone/CHCl3 solvent mixture at rt.*).[8]

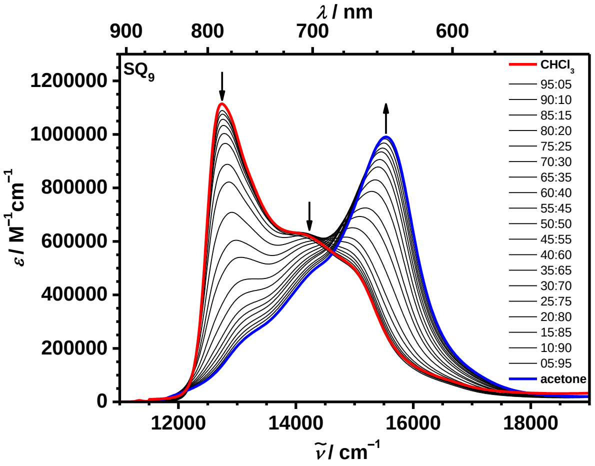

Figure S10. Absorption spectra of **SQ$_9$** in acetone/$CHCl_3$ solvent mixture at rt. A concentration of about 7×10^{-7} M was used.

The mole fraction of the coil f_c was determined using the extinction coefficients at 12746 cm^{-1}:

$$f_C = \frac{(\varepsilon_H - \varepsilon)}{(\varepsilon_H - \varepsilon_C)} \qquad \text{S4}$$

where ε_H is the extinction coefficient of the helix, ε_C is the extinction coefficient of the coil and ε is the extinction coefficient at 12746 cm^{-1}.

Vice versa, the mole fraction of the helix f_H was determined using the extinction coefficients at 15532 cm^{-1}:

$$f_H = 1 - \frac{(\varepsilon_H - \varepsilon)}{(\varepsilon_H - \varepsilon_C)} \qquad \text{S5}$$

The equilibrium constant K for each solvent mixture was then calculated by Equation S6:

$$K = \frac{f_H}{f_C} \qquad \text{S6}$$

and K then was used to calculate the Gibbs free energy ΔG:

$$\Delta G = -RT\ln K \qquad \text{S7}$$

where R is the gas constant and T the temperature. Ploting ΔG vs. the solvent volume fraction yields the Gibbs free energies of **SQ$_9$** for the pure solvents by extrapolation, see Figure S11.

a)

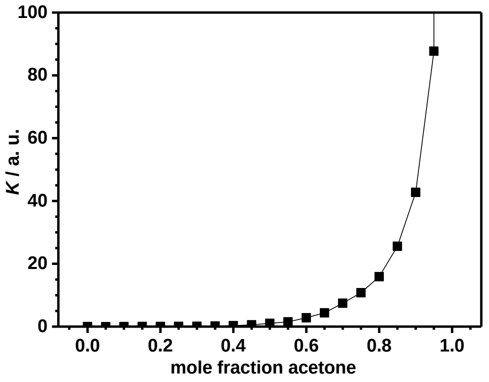

b)

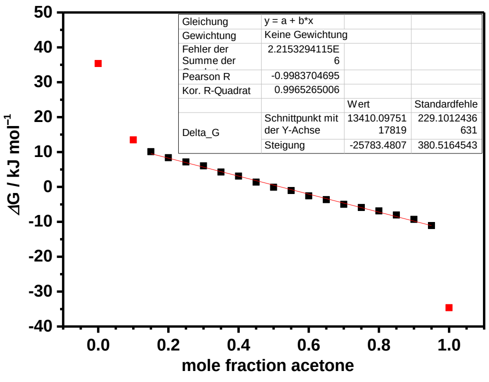

Figure S11. a) Equilibrium constant determined by eq. (S4-S6) depending on acetone/$CHCl_3$ solvent mixture. (b) Gibbs free energy of helix formation determined by eq. (S7) and extrapolation to pure $CHCl_3$ and acetone by linear least squared fit. The red data points in (b) are excluded from the linear fit because for equilibrium constants approaching either zero or infinity the associated Gibbs free energy is unreliable.

4 NMR Spectroscopy

For the characterisation of purity and identity we performed ^{1}H and ^{13}C NMR spectroscopy of the oligomers, see peak list in the synthetic part of the SI and the following figures.

2-(2,3,3-Trimethyl-3*H*-indol-5-yl)-2,3-dihydro-1*H*-naphtho[1,8-de][1,3,2]diaza-borinine (2)

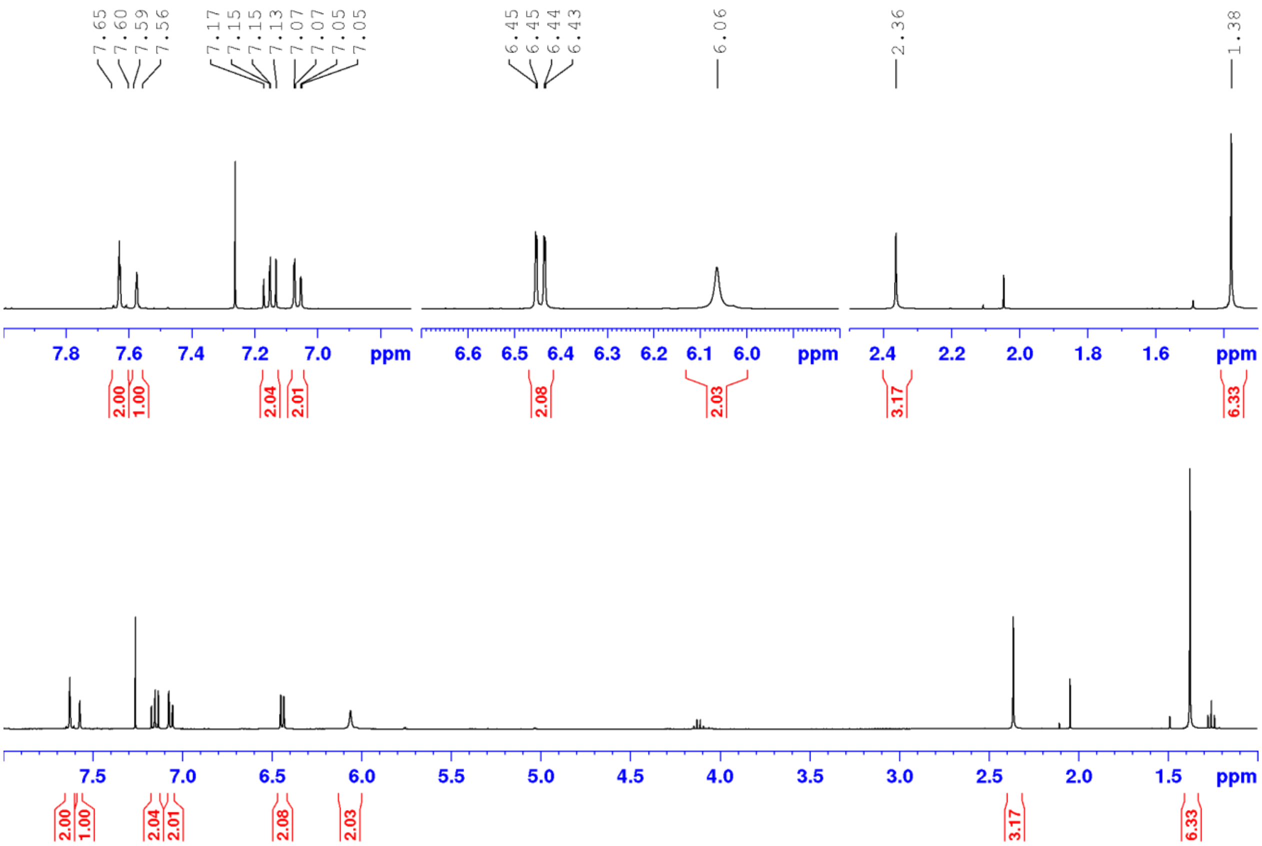

Figure S12. ^{1}H NMR ($CDCl_3$, 400 MHz) of compound **2**.

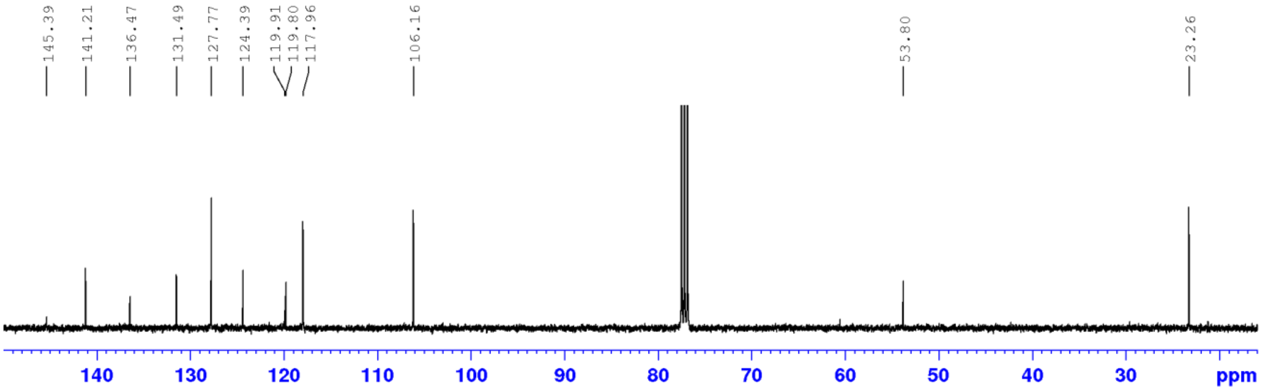

Figure S13. ^{13}C NMR ($CDCl_3$, 100 MHz) of compound **2**.

1-(3,7-dimethyloctyl)-2,3,3-trimethyl-5-(1*H*-naphtho[1,8-de][1,3,2]diazaborinin-2(3*H*)-yl)-3*H*-indol-1-ium iodide (3)

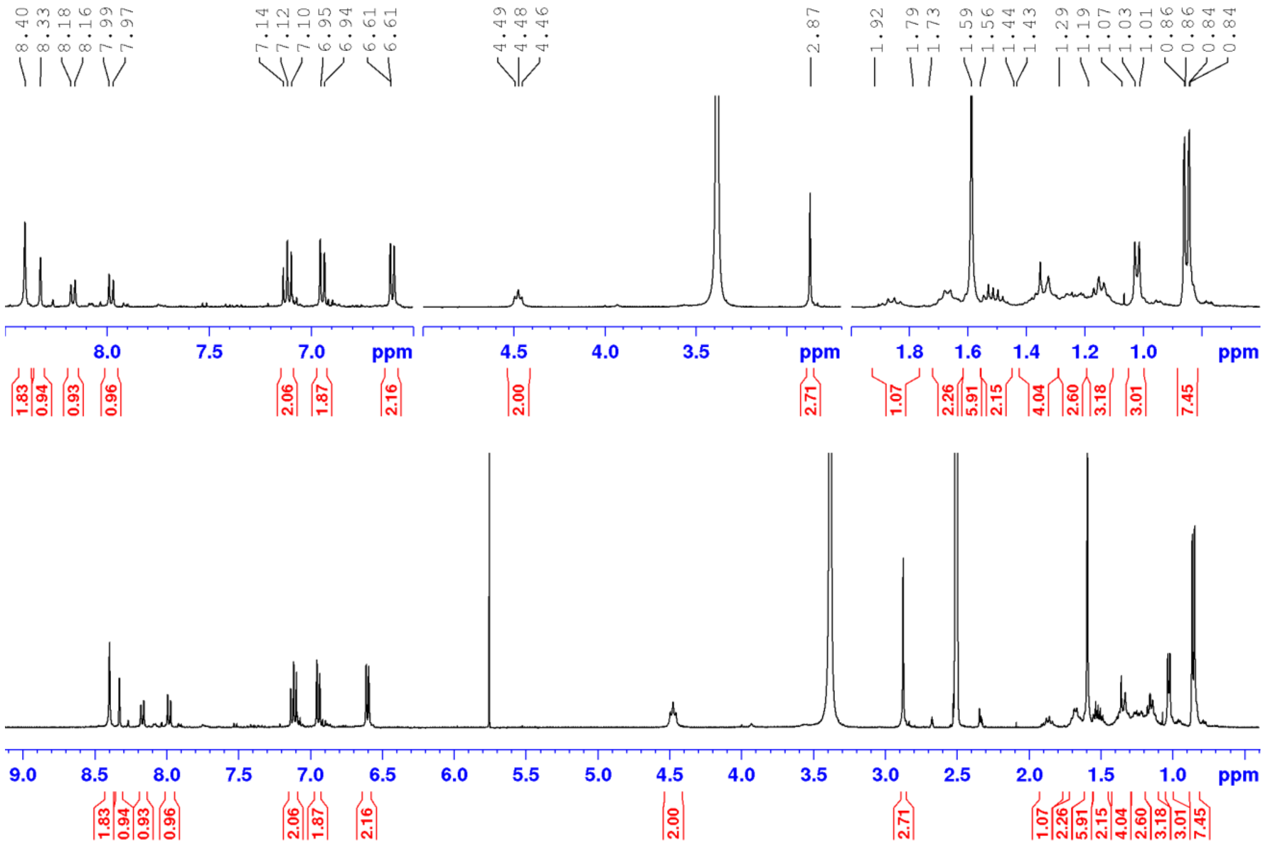

Figure S14. ^{1}H NMR (DMSO-d_6, 400 MHz) of compound **3**.

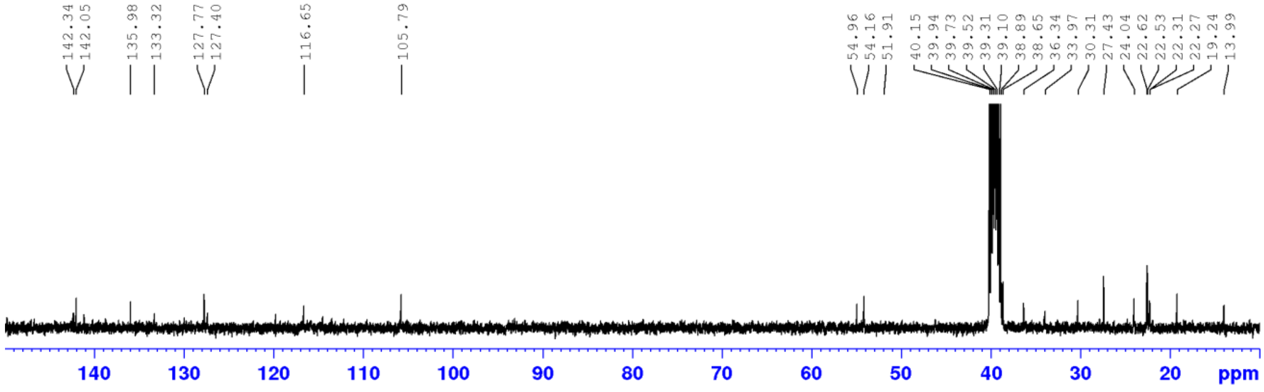

Figure S15. ^{13}C NMR (DMSO-d_6, 100 MHz) of compound **3**.

SQ-BrBdan

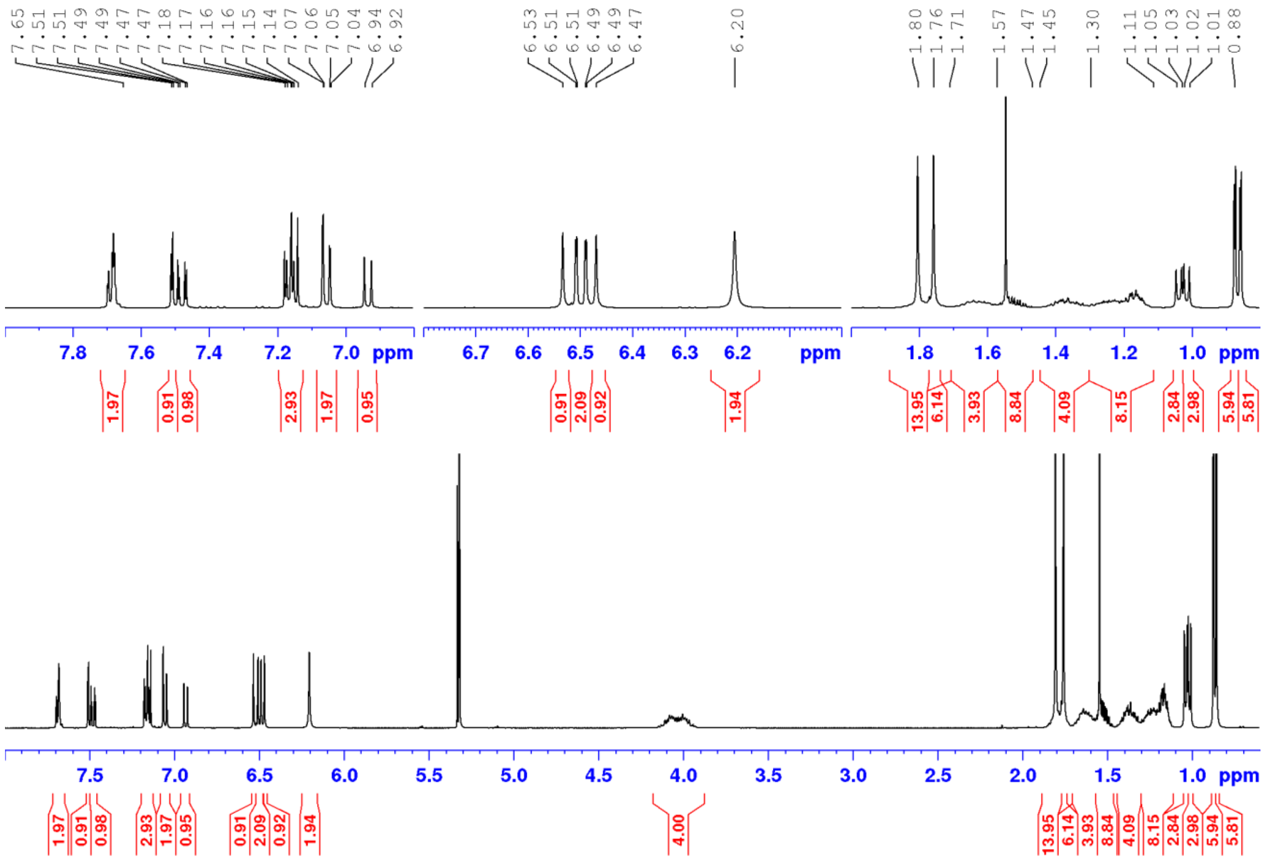

Figure S16. ^{1}H NMR (CD_2Cl_2, 400 MHz) of **SQ-BrBdan**.

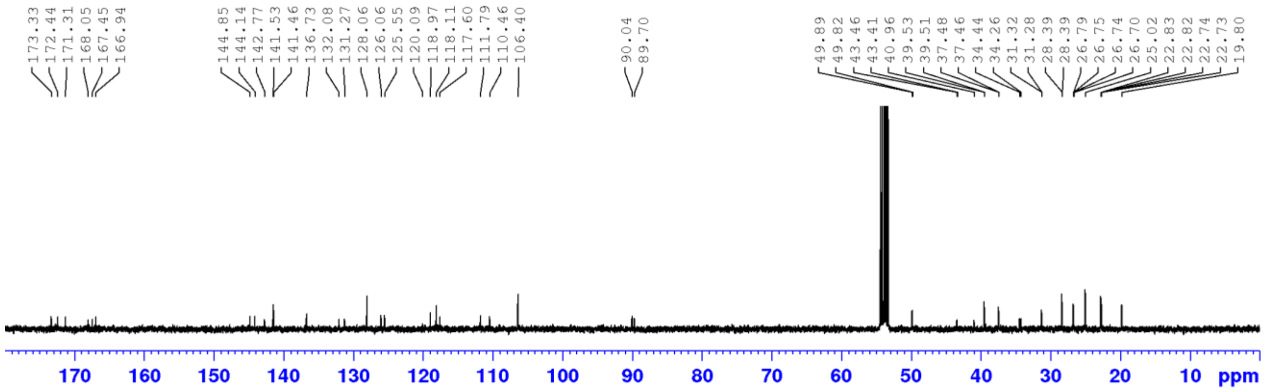

Figure S17. ^{13}C NMR (CD_2Cl_2, 100 MHz) of **SQ-BrBdan**.

SQ-BpinBdan

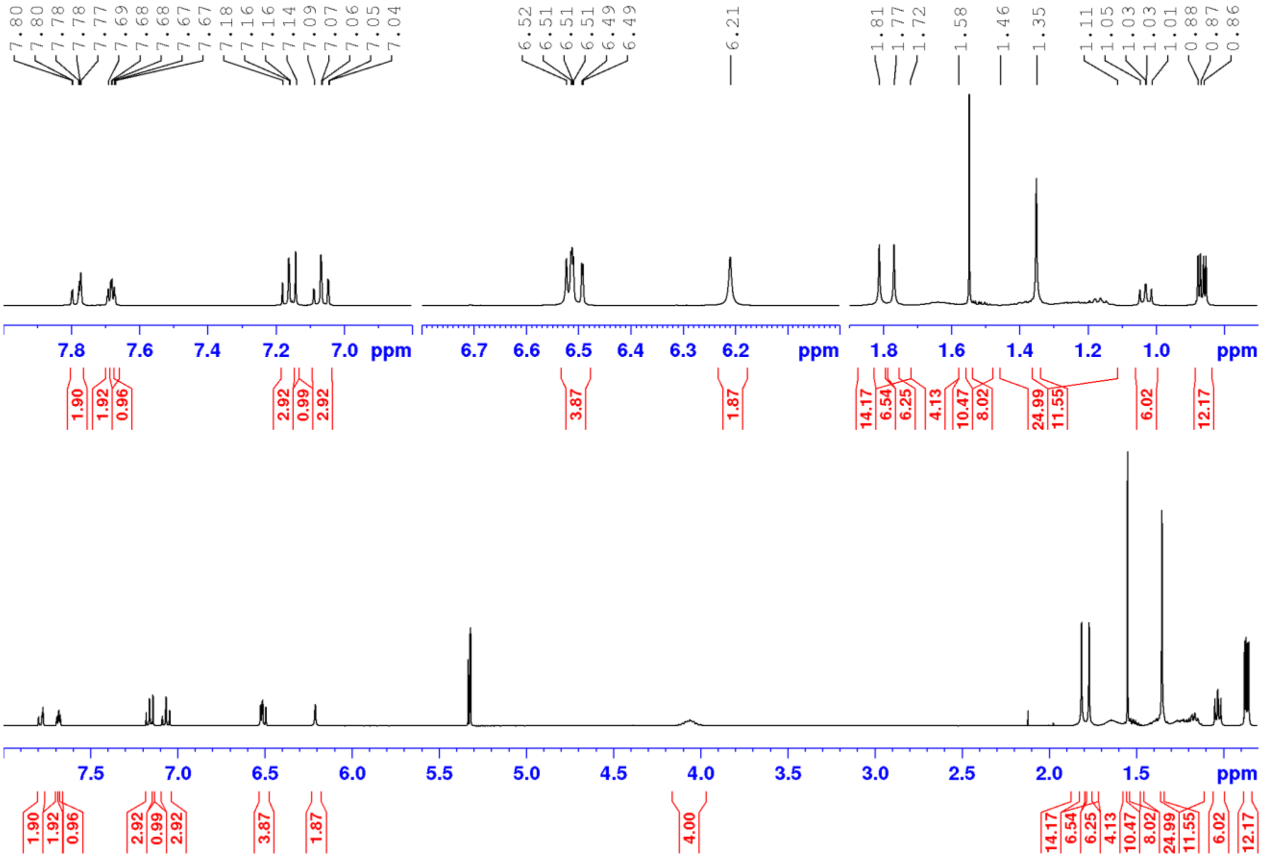

Figure S18. ^{1}H NMR (CD_2Cl_2, 400 MHz) of **SQ-BpinBdan**.

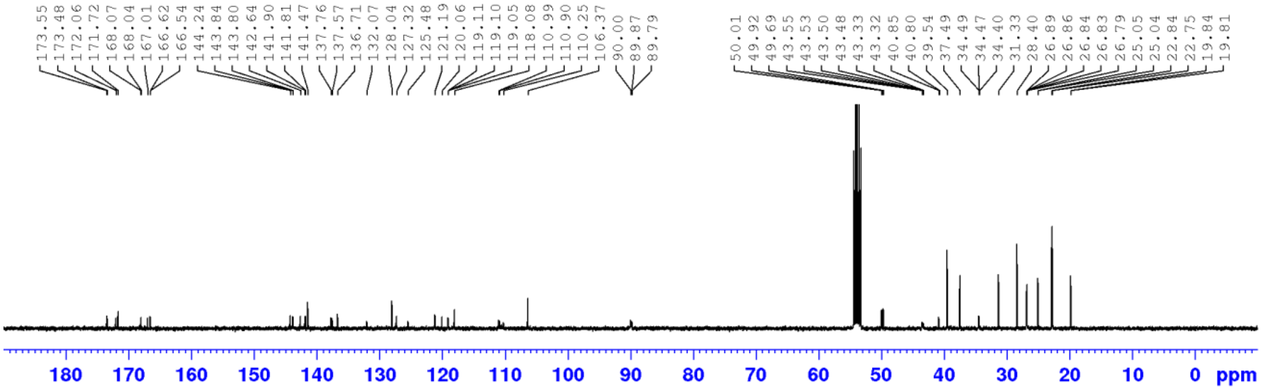

Figure S19. ^{13}C NMR (CD_2Cl_2, 100 MHz) of **SQ-BpinBdan**.

SQ_2-Bdan$_2$

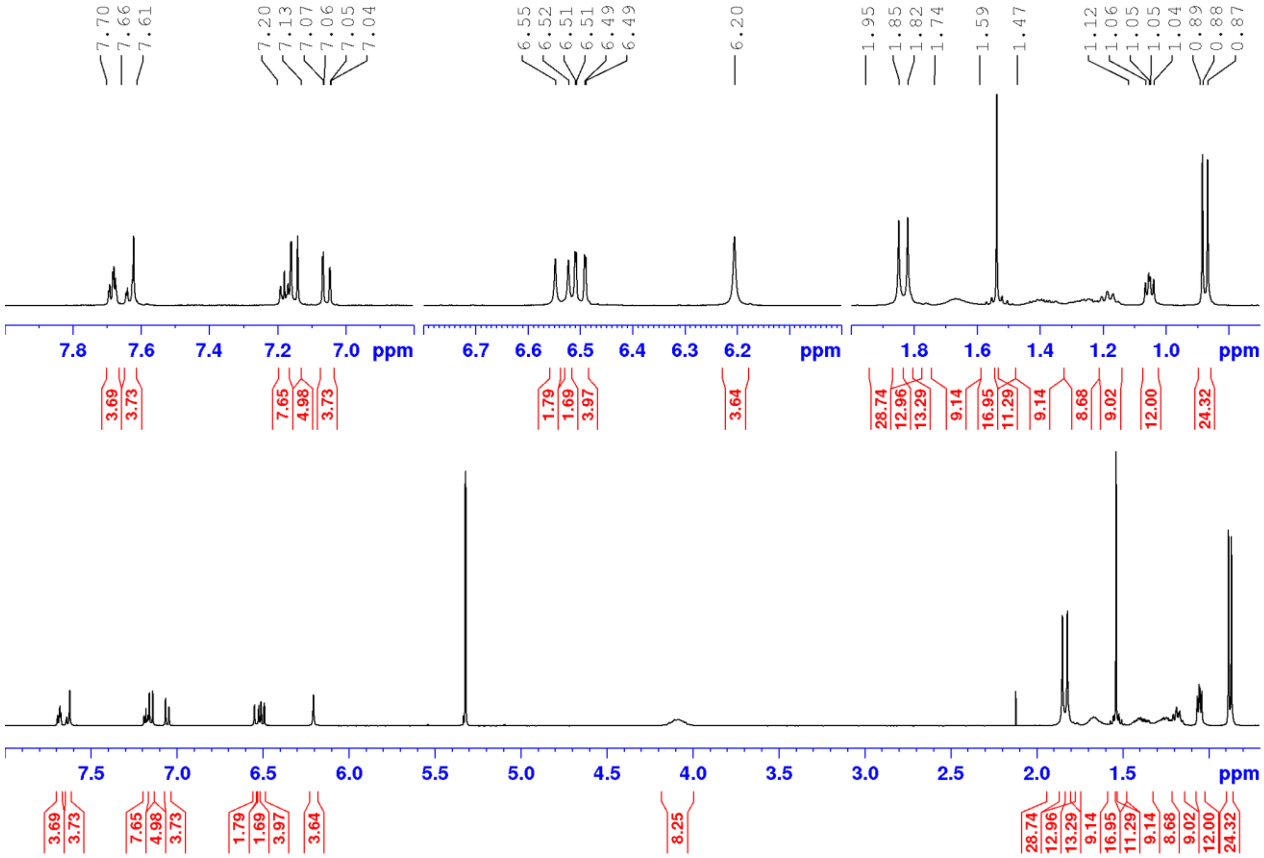

Figure S20. ^{1}H NMR (CD_2Cl_2, 400 MHz) of **SQ_2-Bdan$_2$**.

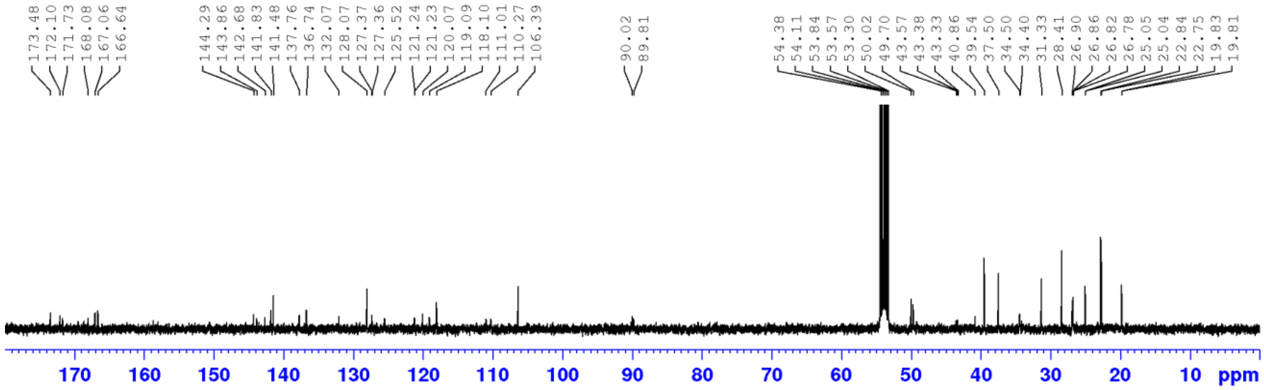

Figure S21. ^{13}C NMR (CD_2Cl_2, 100 MHz) of **SQ_2-Bdan$_2$**.

SQ_3

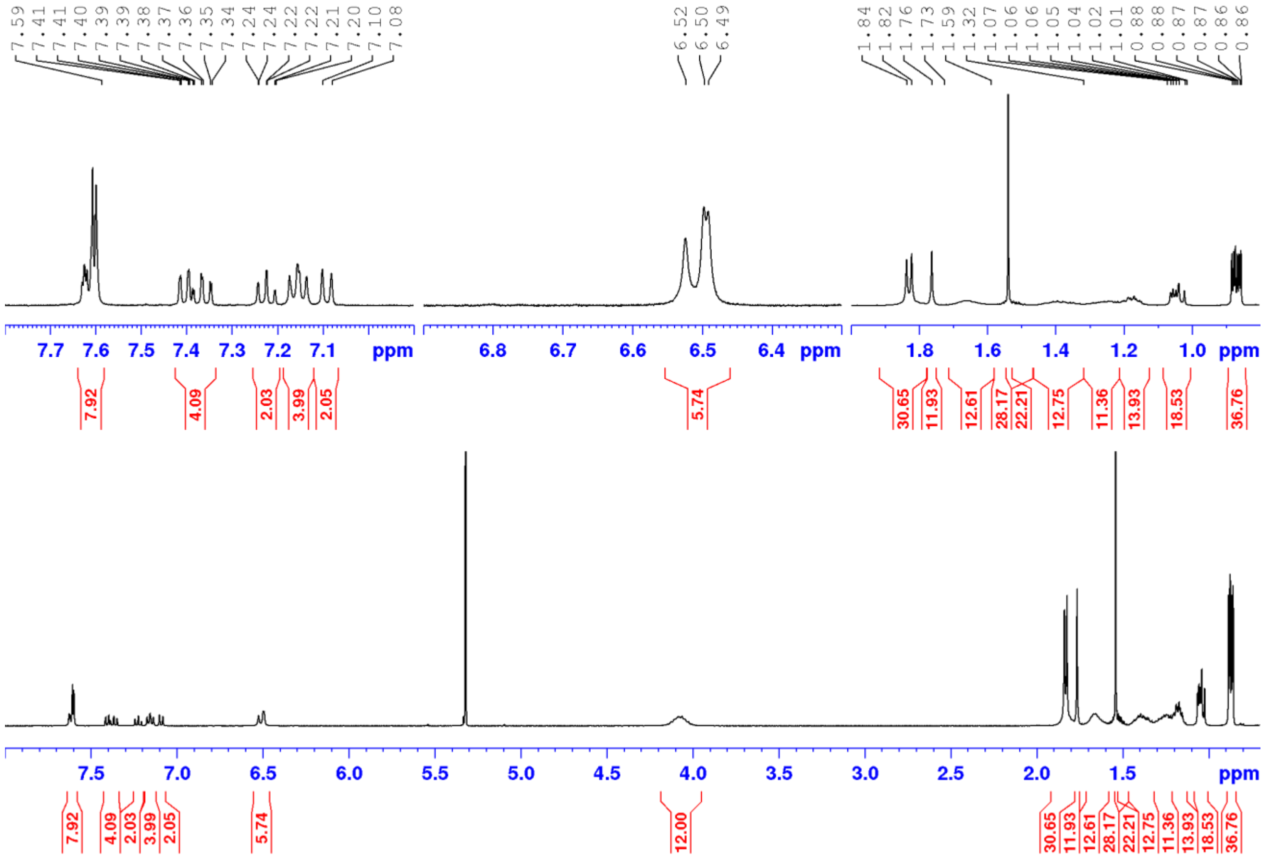

Figure S22. ^1H NMR (CD_2Cl_2, 400 MHz) of **SQ_3**.

SQ₃-Bdan₂

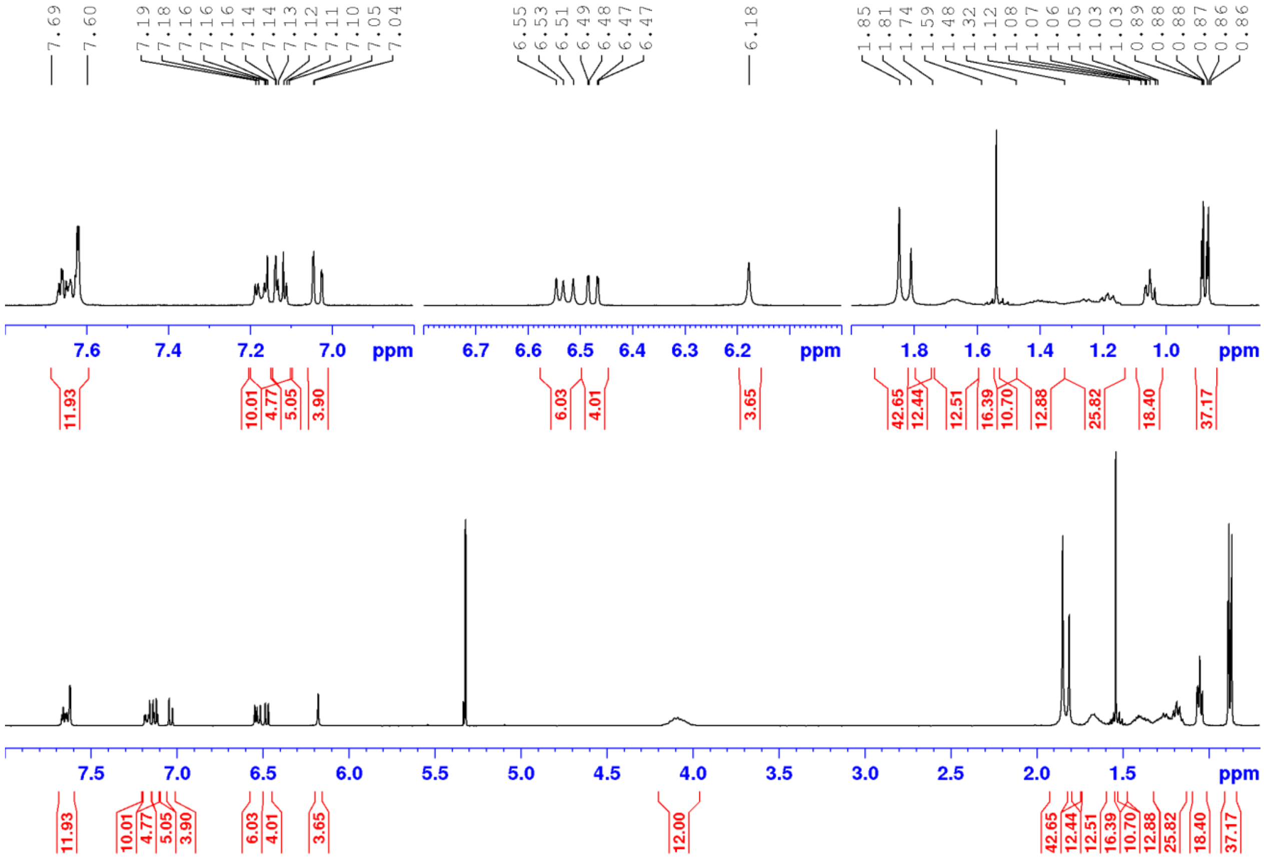

Figure S23. ^{1}H NMR (CD_2Cl_2, 400 MHz) of **SQ_3-Bdan$_2$**.

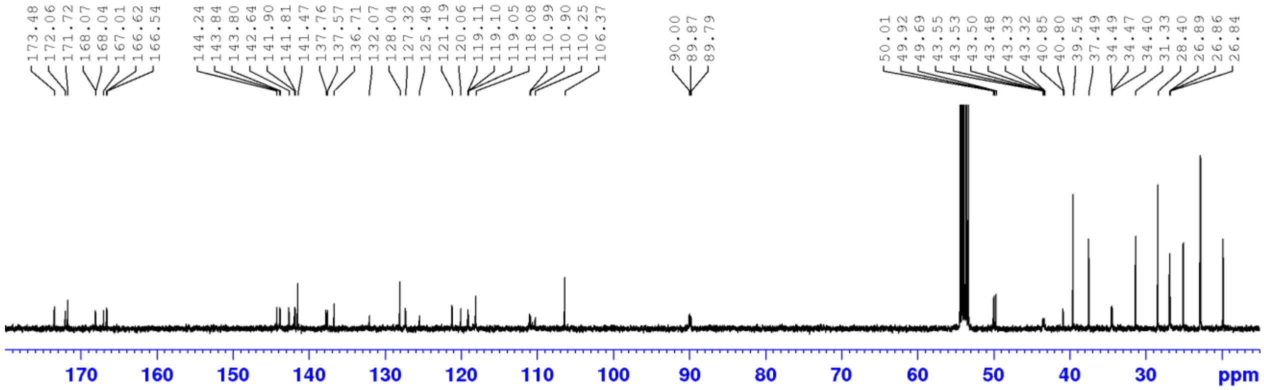

Figure S24. ^{13}C NMR (CD_2Cl_2, 100 MHz) of **SQ_3-Bdan$_2$**.

SQ4

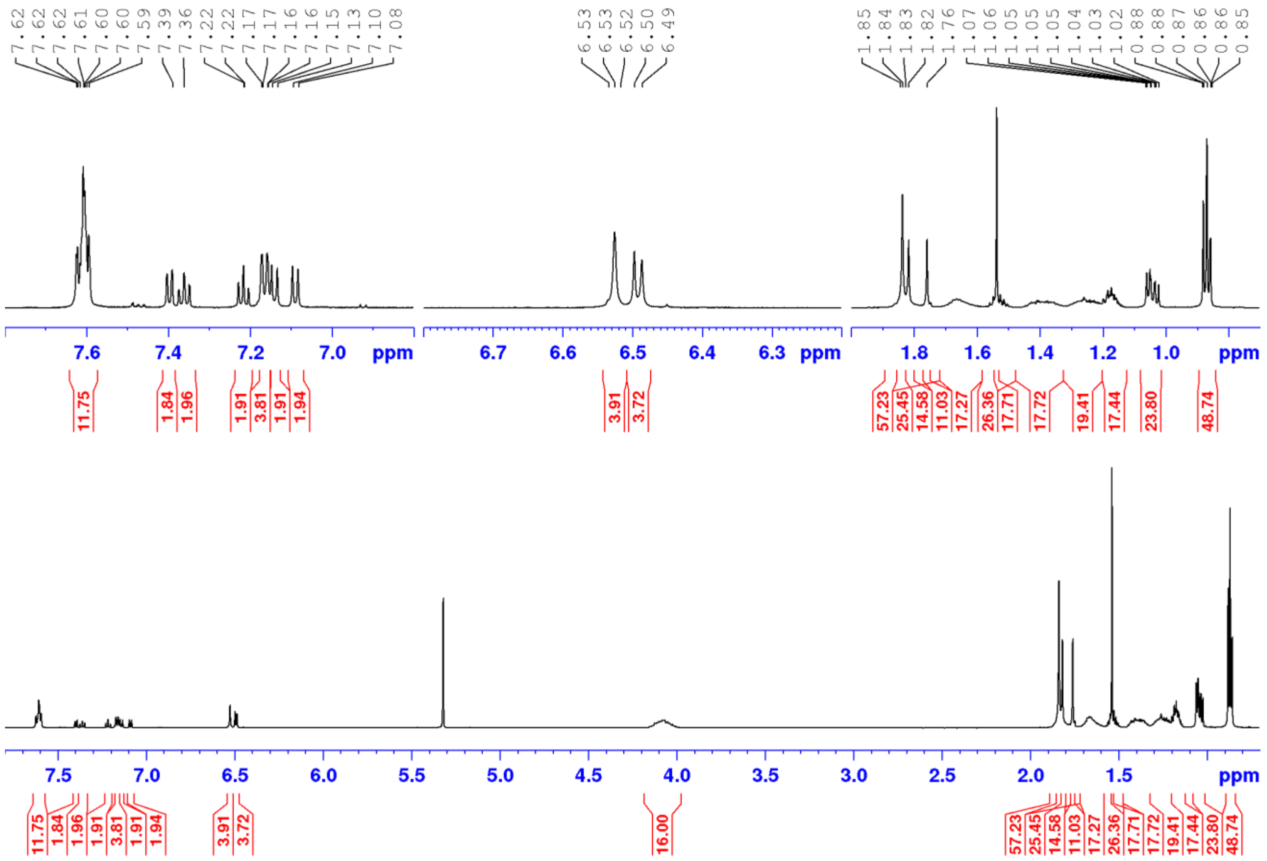

Figure S25. ¹H NMR (CD_2Cl_2, 600 MHz) of **SQ4**.

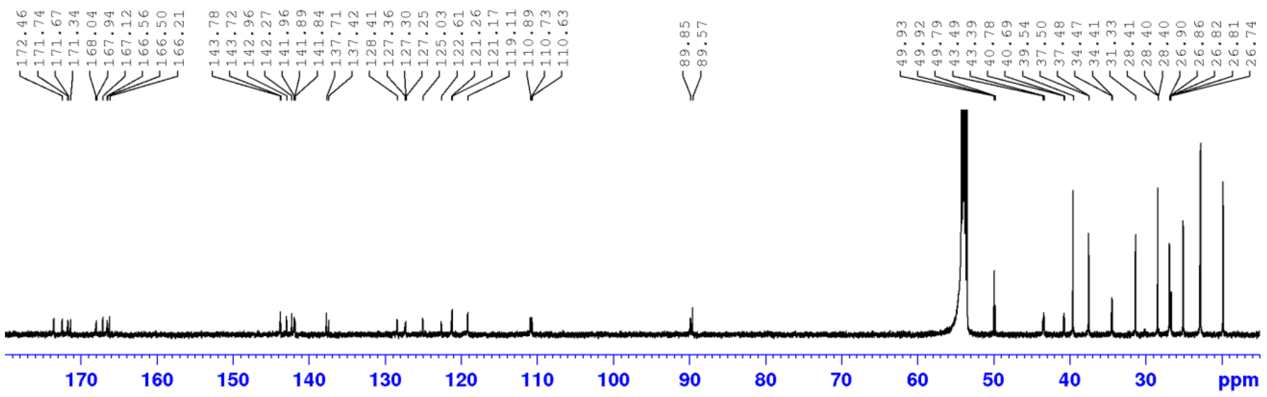

Figure S26. ¹³C NMR (CD_2Cl_2, 150 MHz) of **SQ4**.

SQ_4-Bdan$_2$

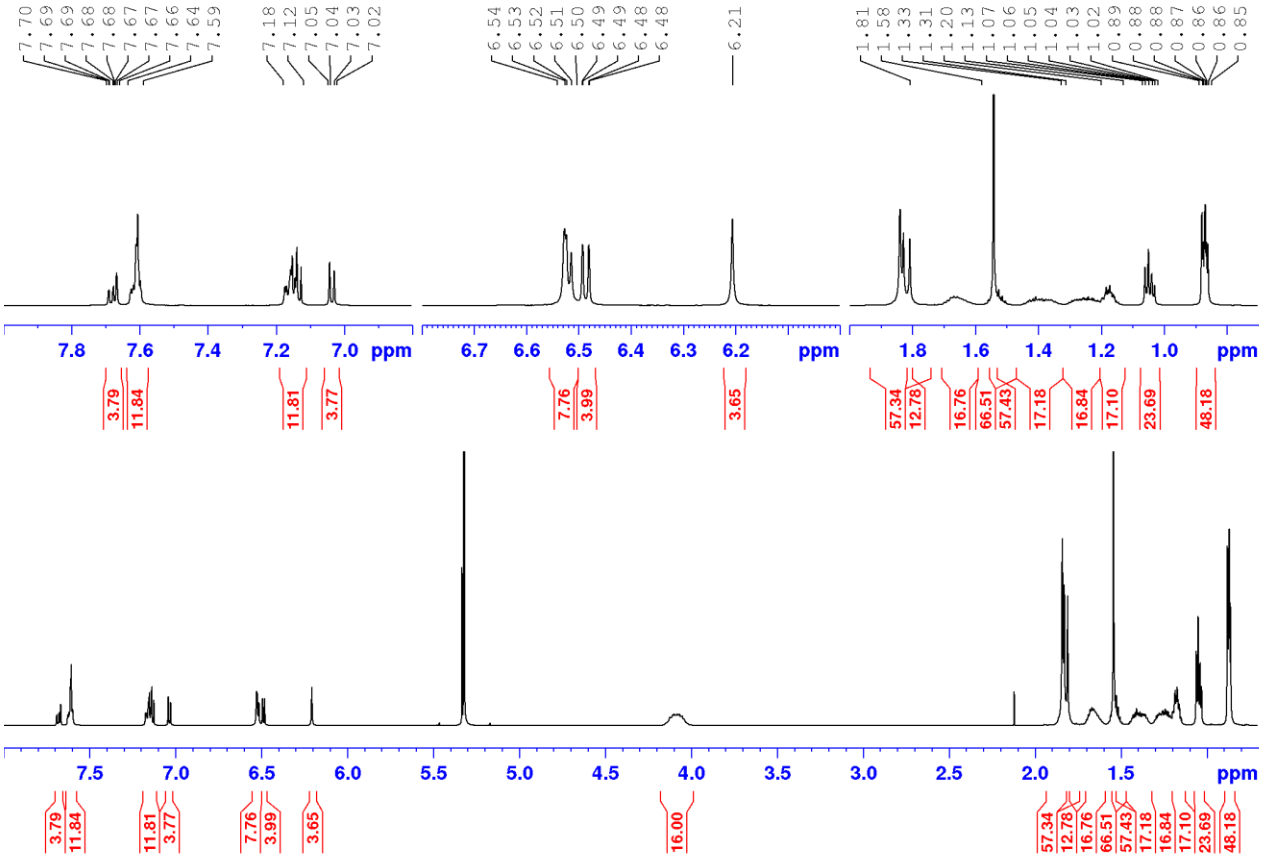

Figure S27. ^{1}H NMR (CD_2Cl_2, 600 MHz) of **SQ_4-Bdan$_2$**.

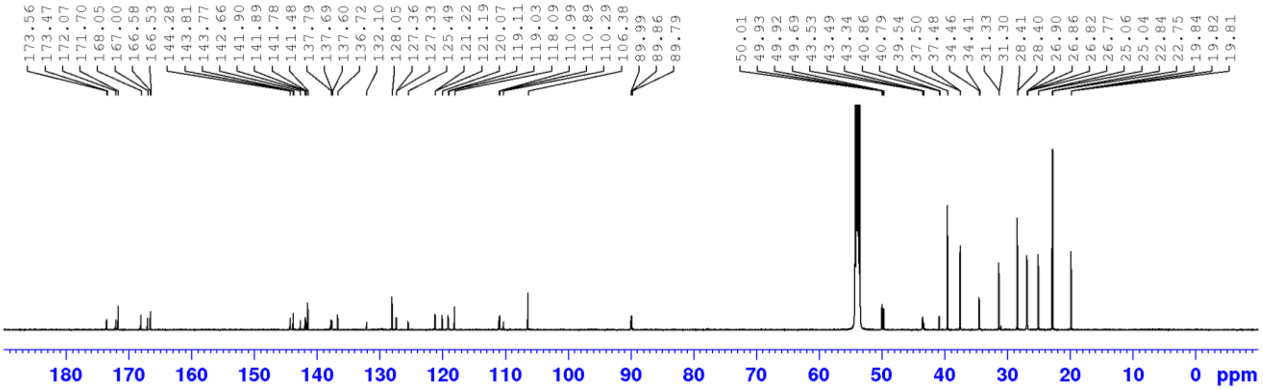

Figure S28. ^{13}C NMR (CD_2Cl_2, 150 MHz) of **SQ_4-Bdan$_2$**.

SQ5

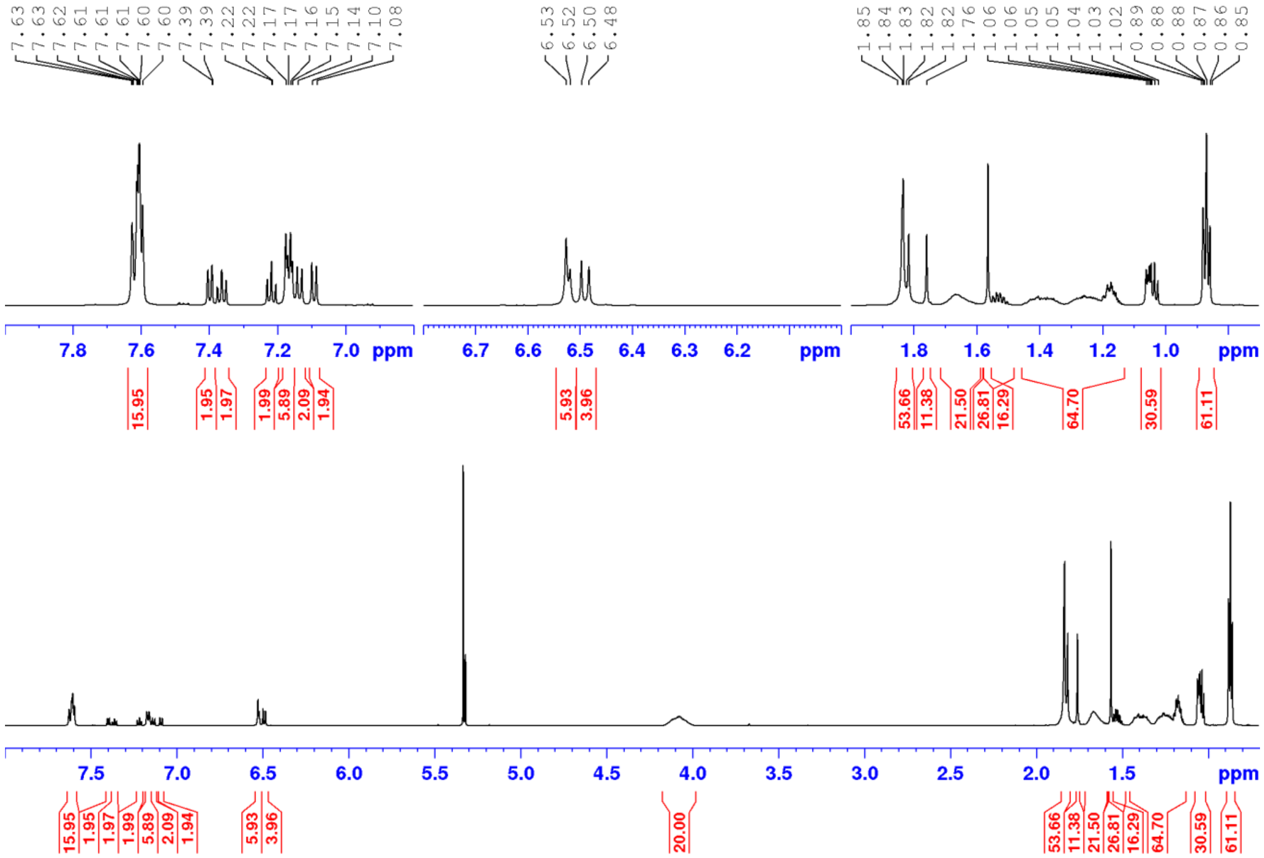

Figure S29. ^{1}H NMR (CD_2Cl_2, 600 MHz) of **SQ5**.

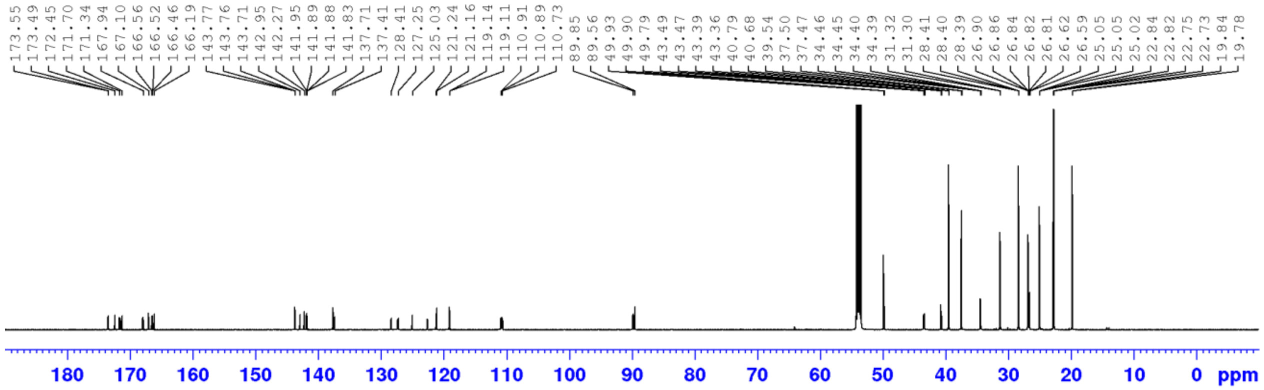

Figure S30. ^{13}C NMR (CD_2Cl_2, 150 MHz) of **SQ5**.

SQ_5-$Bdan_2$

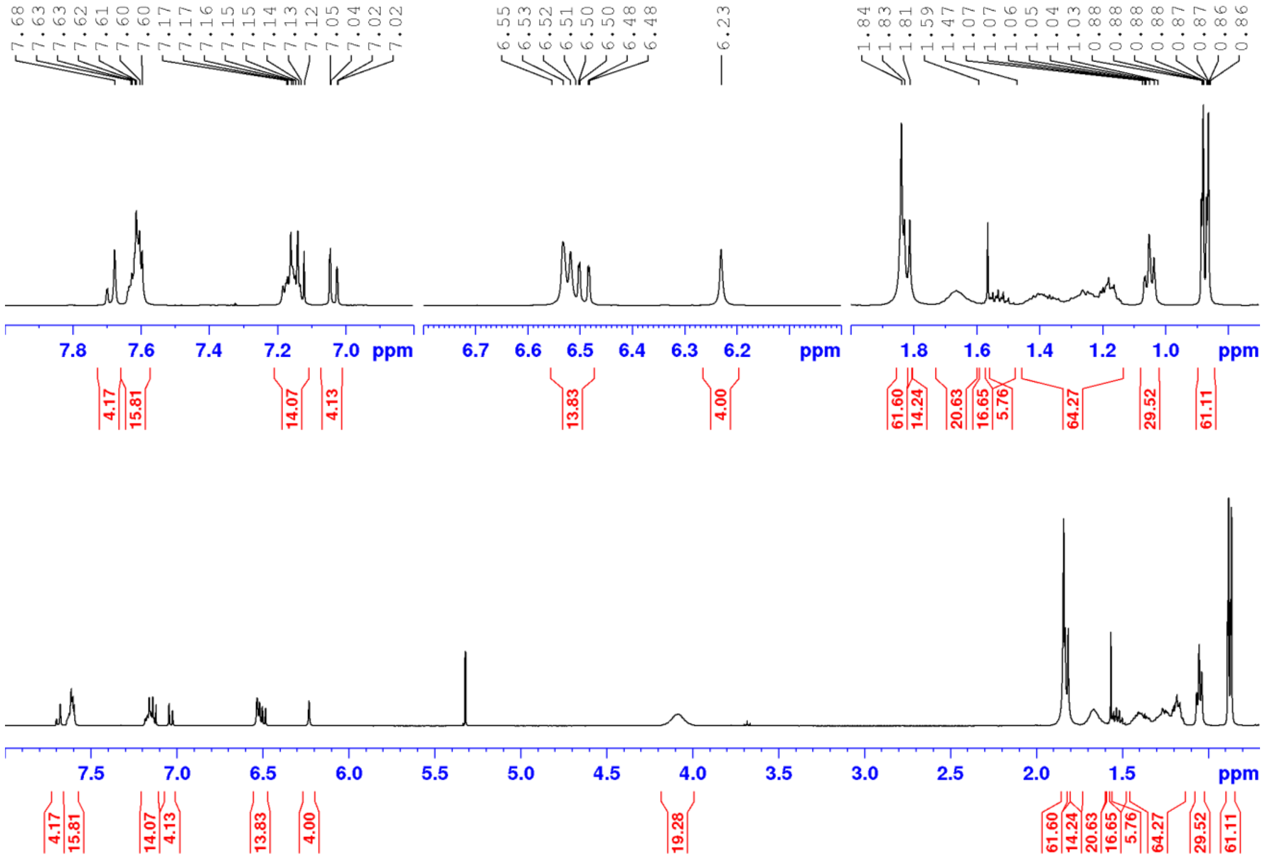

Figure S31. ^{1}H NMR (CD_2Cl_2, 400 MHz) of **SQ_5-$Bdan_2$**.

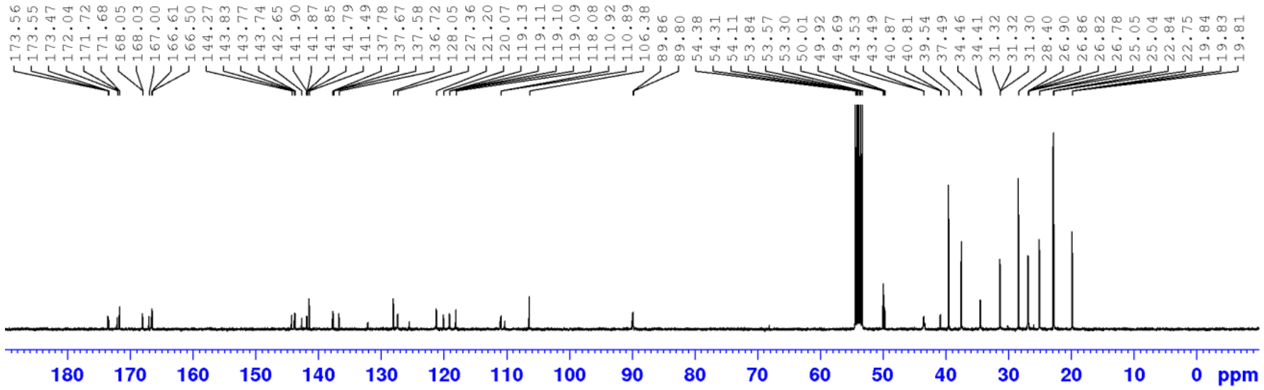

Figure S32. ^{13}C NMR (CD_2Cl_2, 100 MHz) of **SQ_5-$Bdan_2$**.

SQ₆

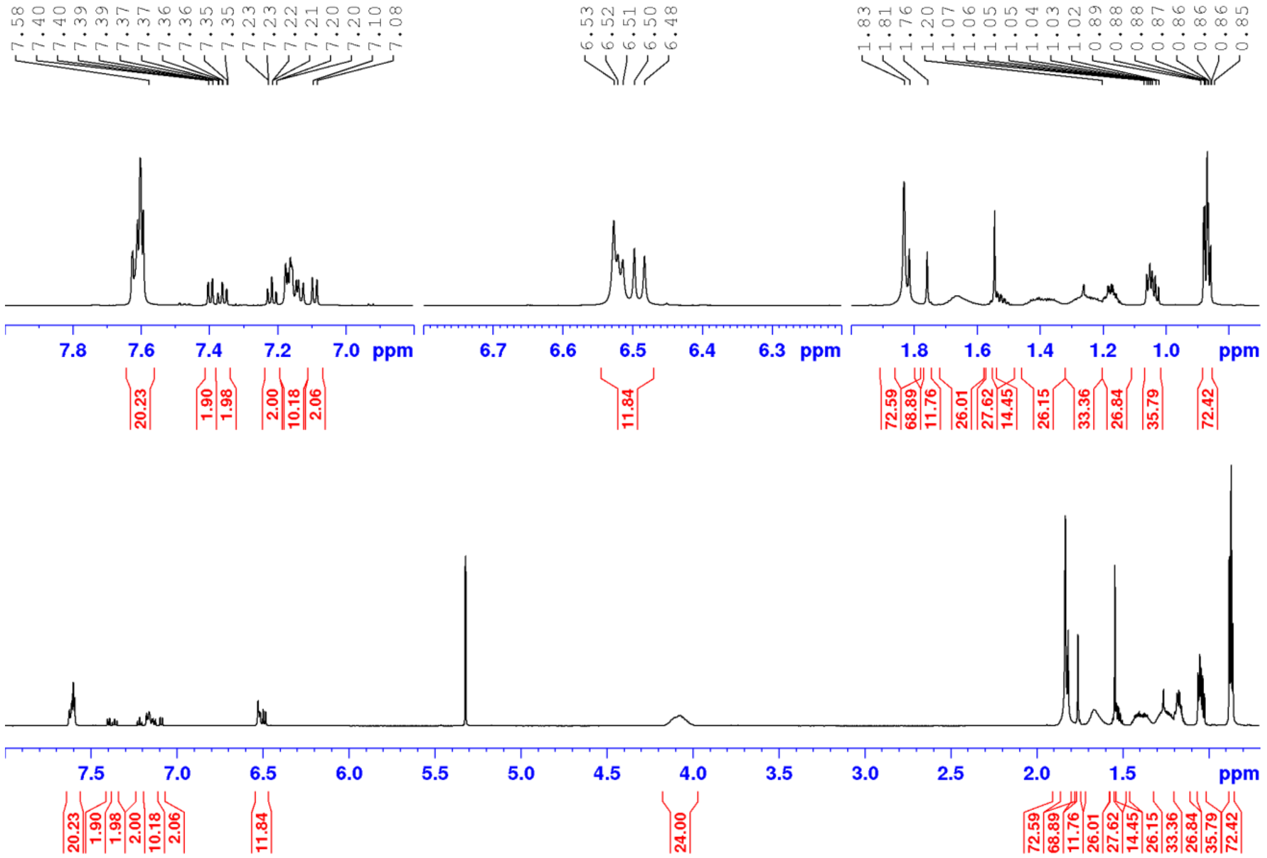

Figure S33. ^{1}H NMR (CD_2Cl_2, 600 MHz) of **SQ_6**.

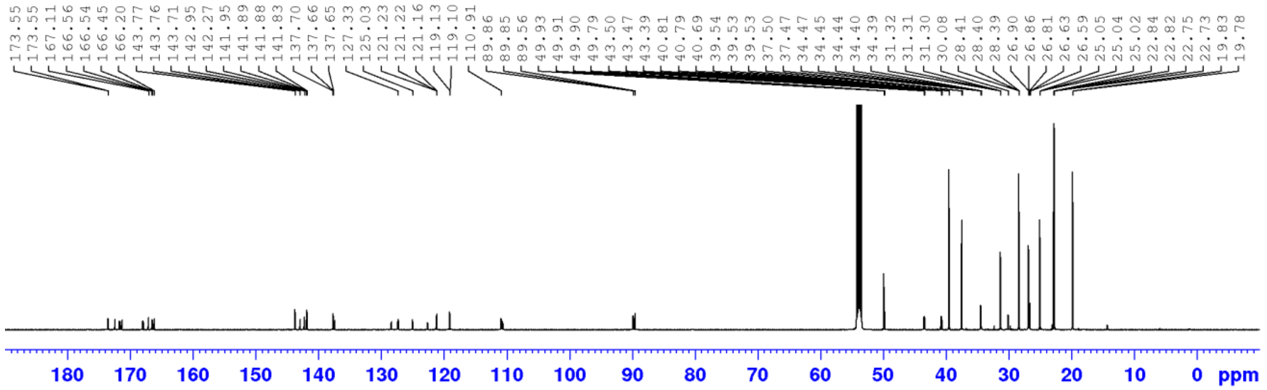

Figure S34. ^{13}C NMR (CD_2Cl_2, 150 MHz) of **SQ_6**.

SQ_6-Bdan$_2$

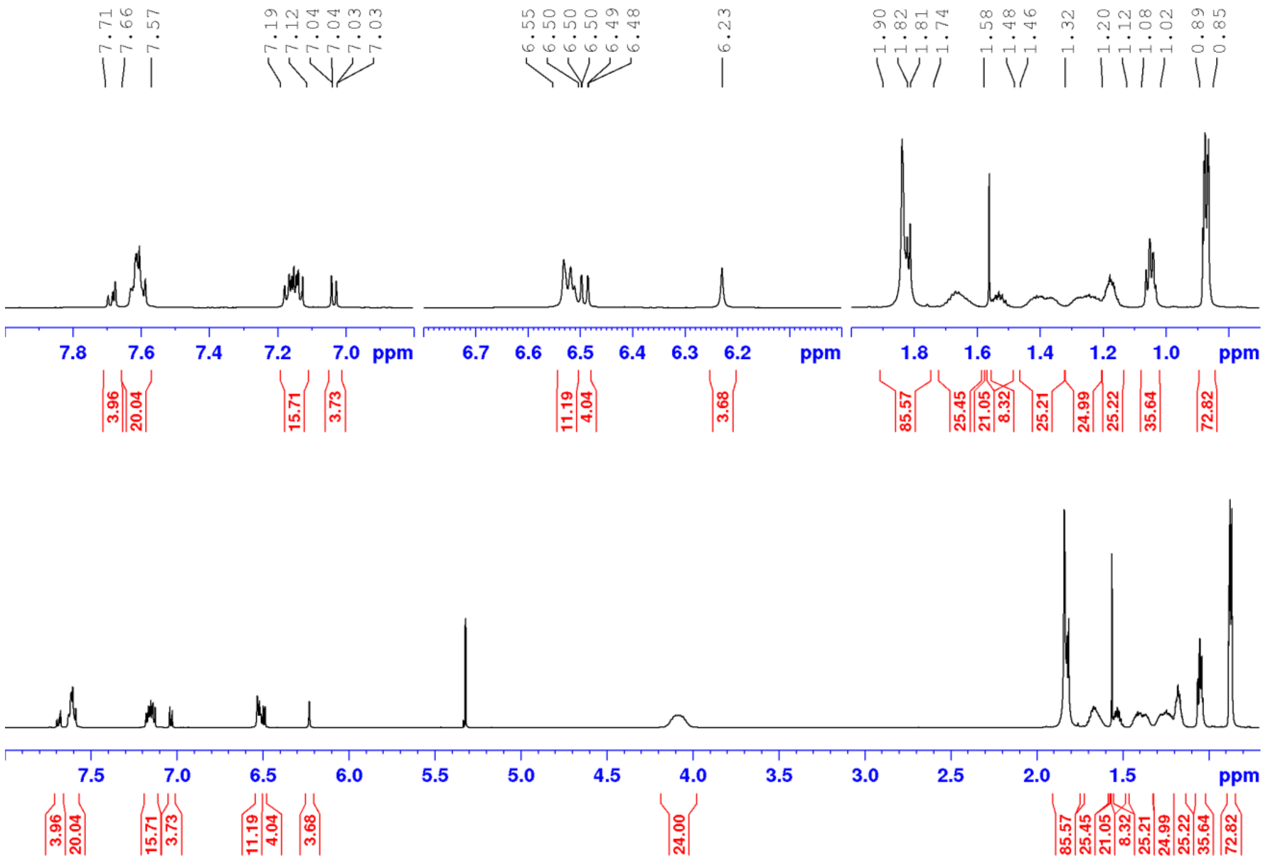

Figure S35. ^{1}H NMR (CD_2Cl_2, 600 MHz) of **SQ_6-Bdan$_2$**.

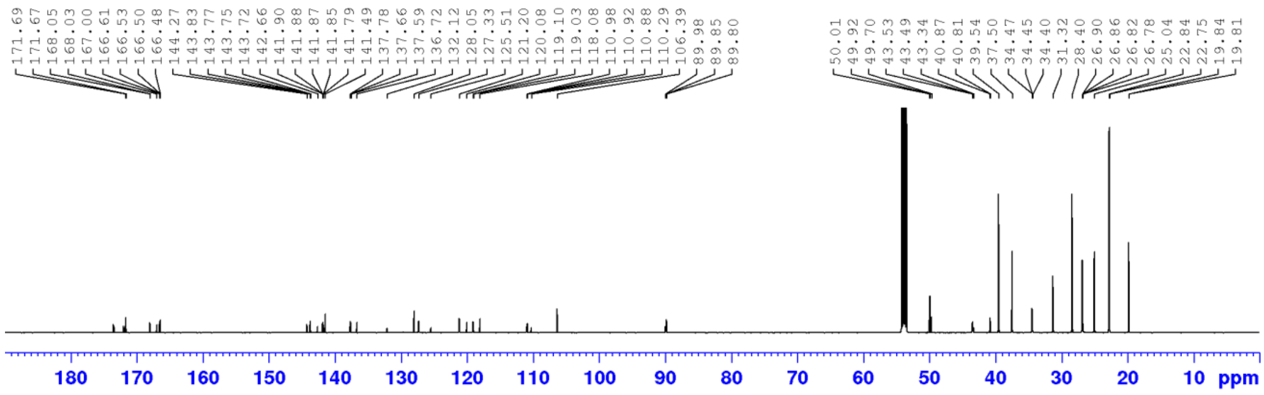

Figure S36. ^{13}C NMR (CD_2Cl_2, 150 MHz) of **SQ_6-Bdan$_2$**.

SQ₇

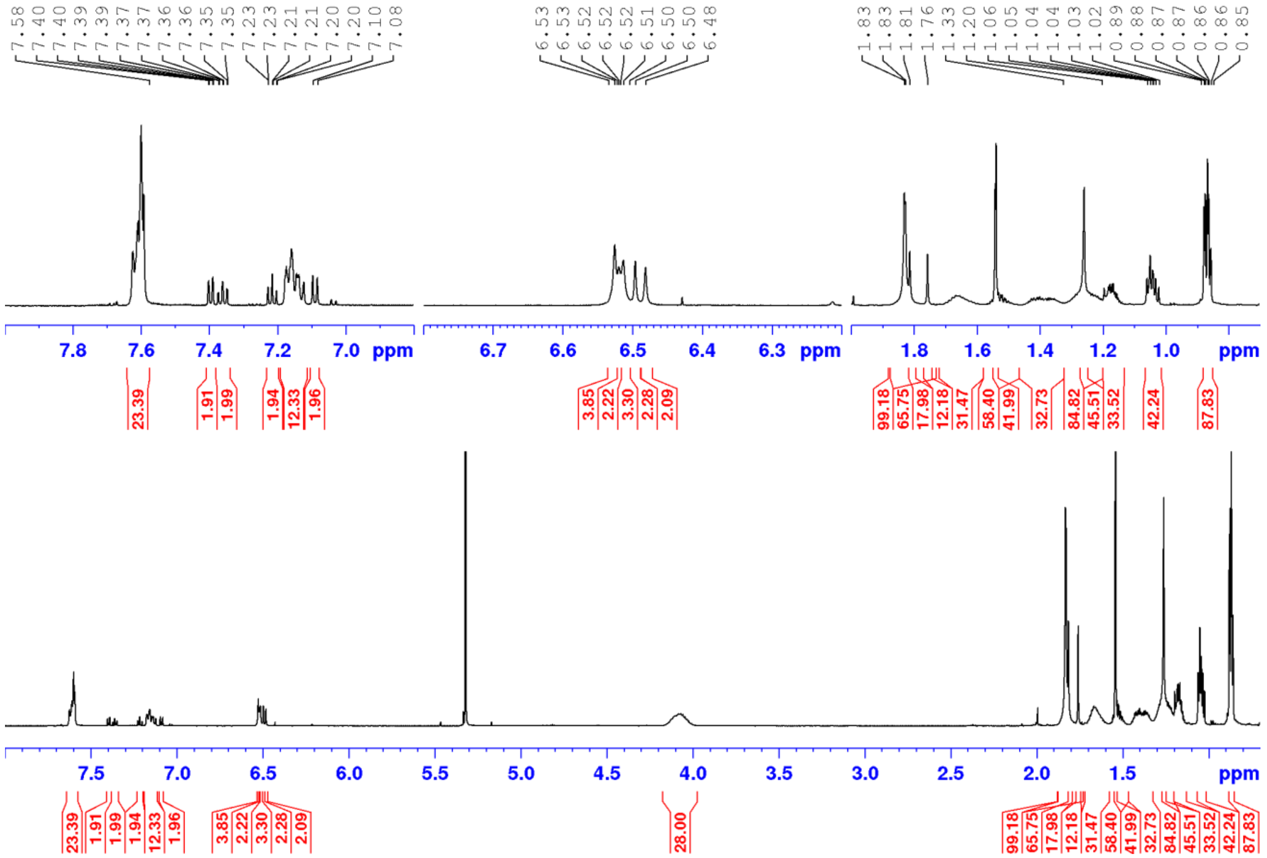

Figure S37. ^{1}H NMR (CD_2Cl_2, 600 MHz) of **SQ_7**.

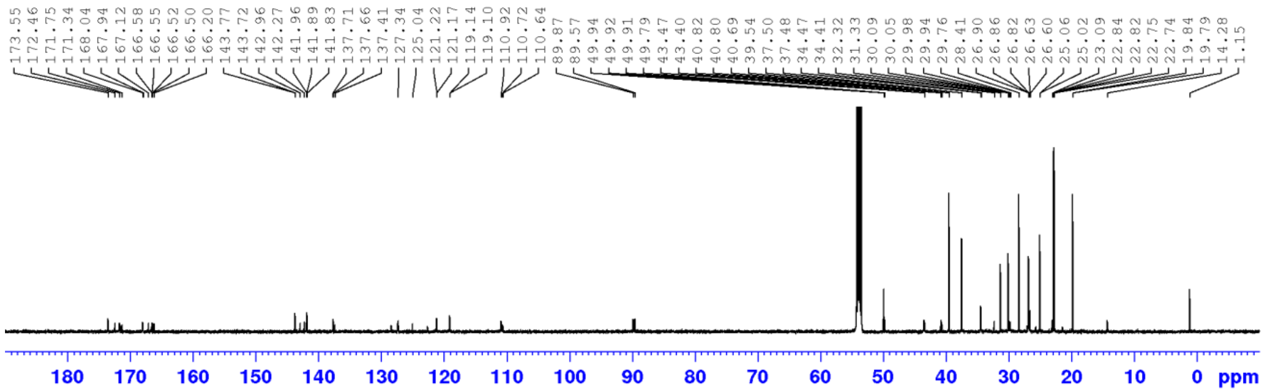

Figure S38. ^{13}C NMR (CD_2Cl_2, 150 MHz) of **SQ_7**.

SQ_7-$Bdan_2$

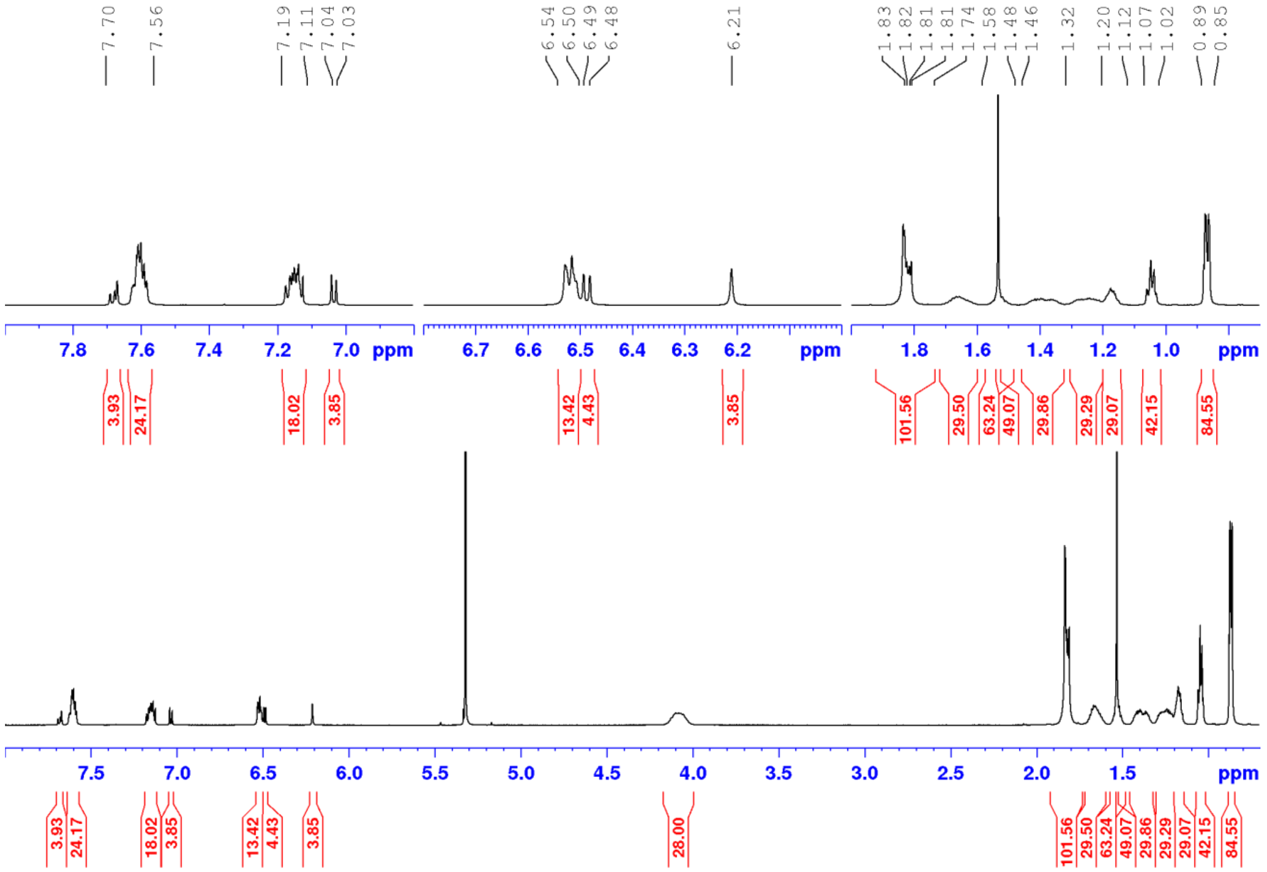

Figure S39. ^{1}H NMR (CD_2Cl_2, 600 MHz) of **SQ_7-$Bdan_2$**.

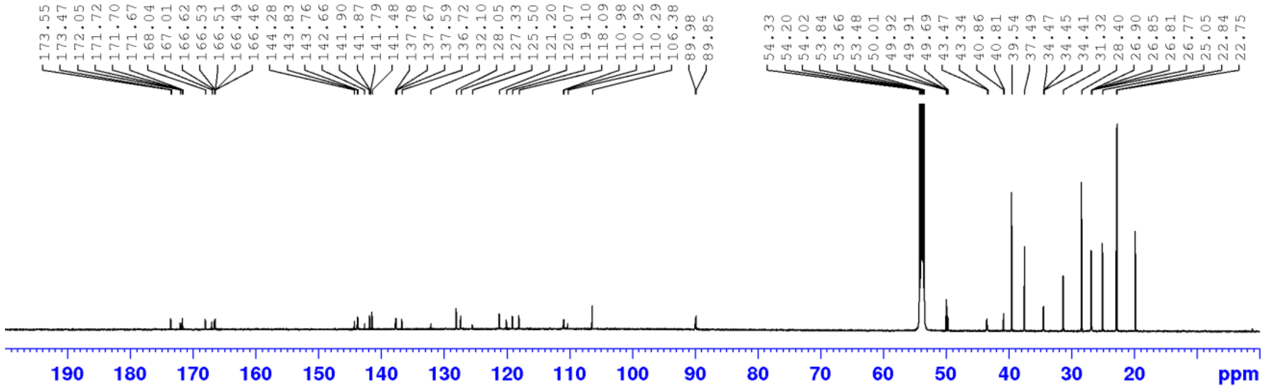

Figure S40. ^{13}C NMR (CD_2Cl_2, 150 MHz) of **SQ_7-$Bdan_2$**.

SQ8

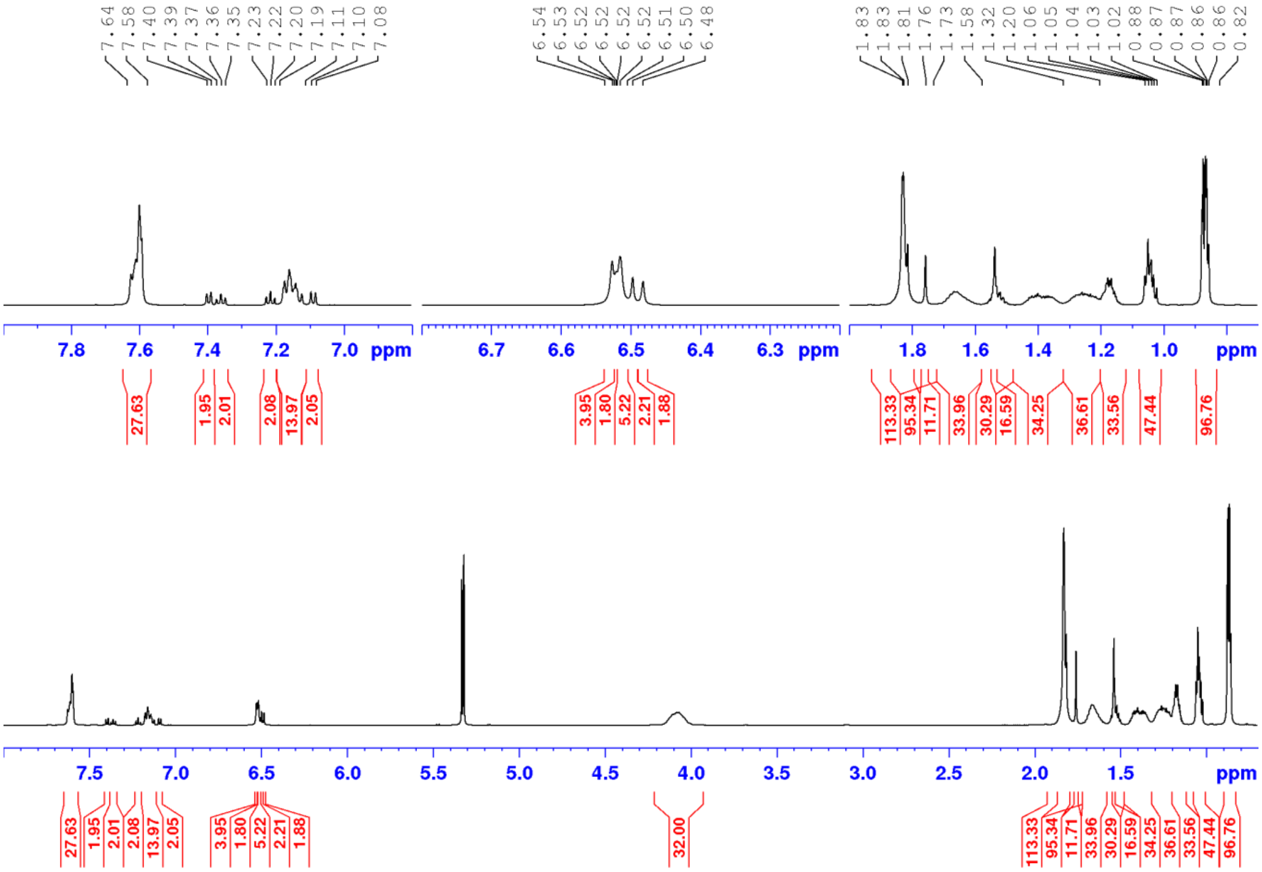

Figure S41. ^{1}H NMR (CD_2Cl_2, 600 MHz) of **SQ8**.

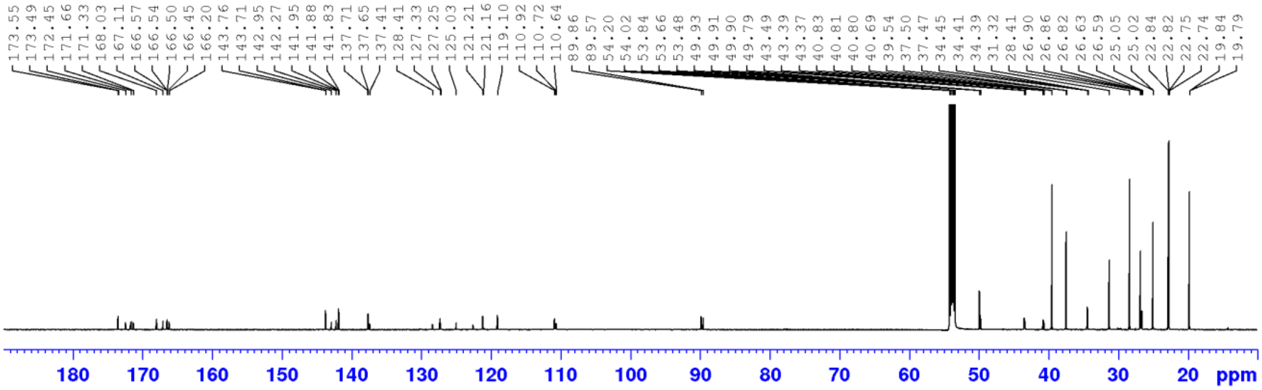

Figure S42. ^{13}C NMR (CD_2Cl_2, 150 MHz) of **SQ8**.

SQ9

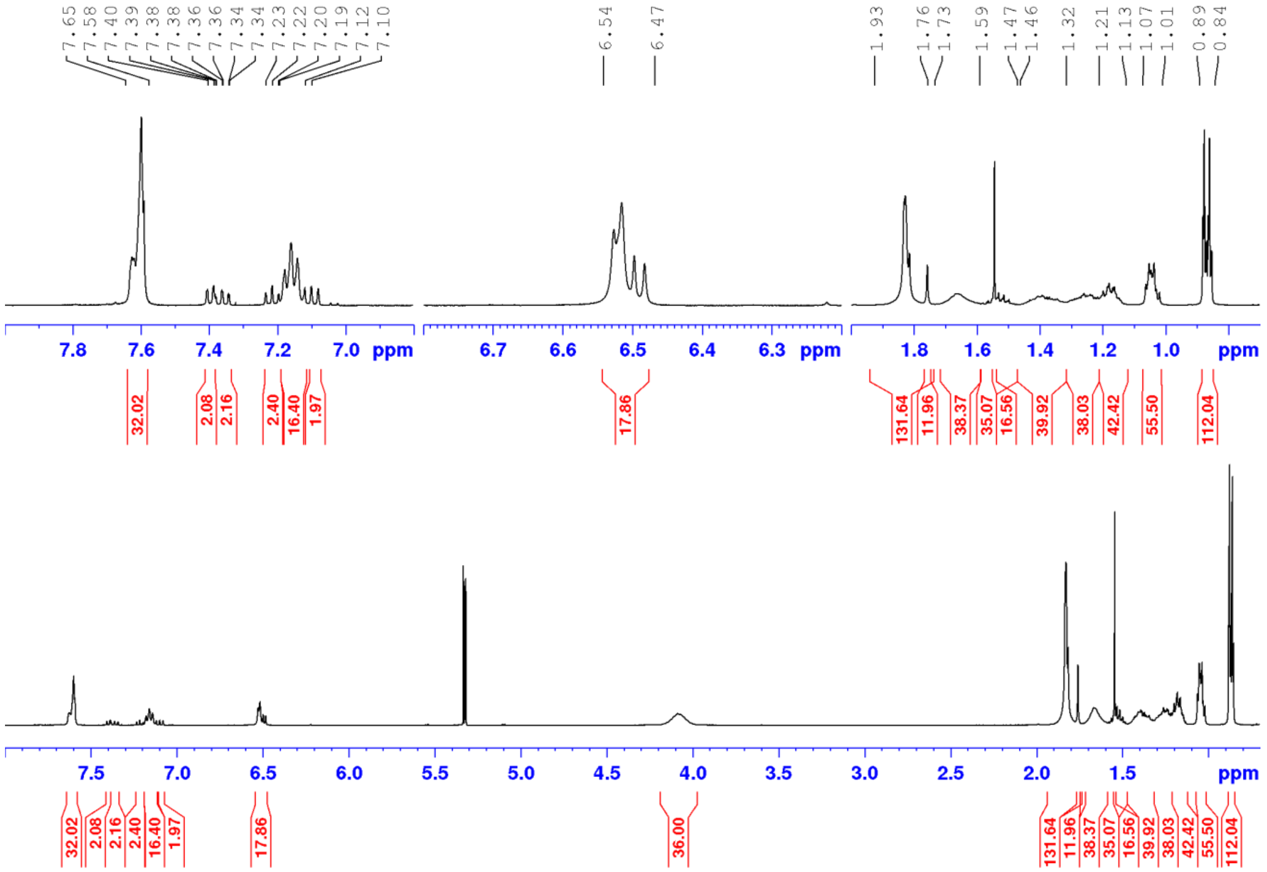

Figure S43. ^{1}H NMR (CD_2Cl_2, 600 MHz) of **SQ9**.

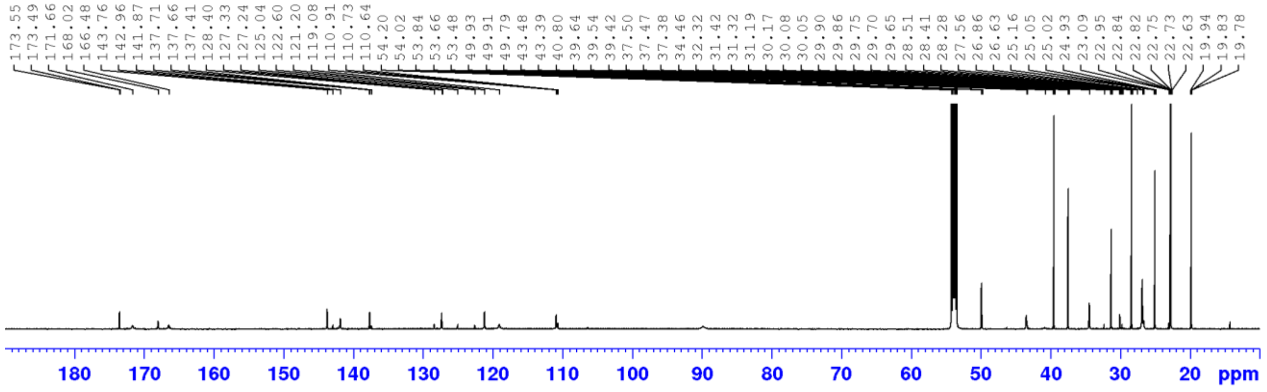

Figure S44. ^{13}C NMR (CD_2Cl_2, 150 MHz) of **SQ9**.

However, NMR spectroscopy proved not to be helpful in order to elucidate the coil-helix transformation. This is due to the fact that even the nonamer does not behave like a polymer, that is, we can discern a large number of e.g. methine protons (see proton H5 in the upper panel of Figure S46) even in CD_2Cl_2 and even more so in acetone-d_6. This is caused by the presence of – in case of the highest oligomer symmetry – at least nine formally chemically different methine positions. For the other aromatic indolenine protons this leads to a plethora of signals which could not specifically be assigned (see Figure S45).

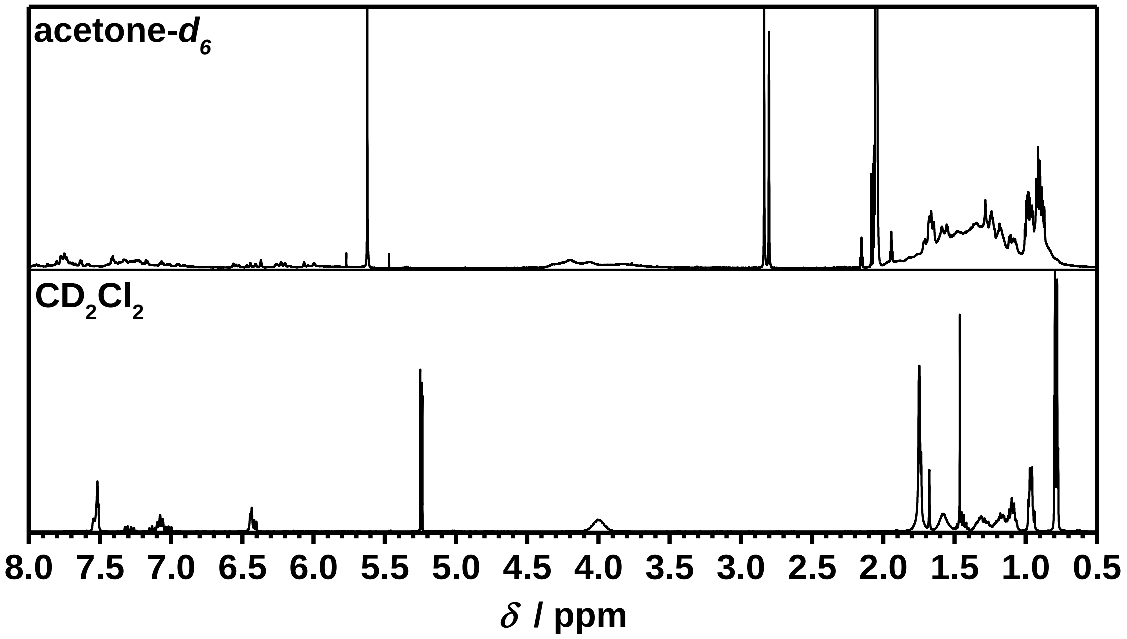

Figure S45. Comparison of ^{1}H NMR spectra of **SQ$_9$** in acetone-d_6 and CD_2Cl_2.

Variable Temperature NMR

In order to prove the coil-helix transition we measured proton NMR spectra in PhCN-d_5 at various temperatures ranging from 268 K to 423 K, see Figure S47. At high temperature, these spectra are very similar to the one in CD_2Cl_2 and at low temperature to the one in acetone-d_6 (see Figure S45). The spectra in-between reflect the coil-helix transition. However, upon rising the temperature, we do not see a decrease of a set of signals that is associated with the helix and, concomitantly, an increasing set of signals for the coil. This is because of exchange phenomena on the NMR time scale which leads to a smooth change of broadened signals upon changing the temperature.

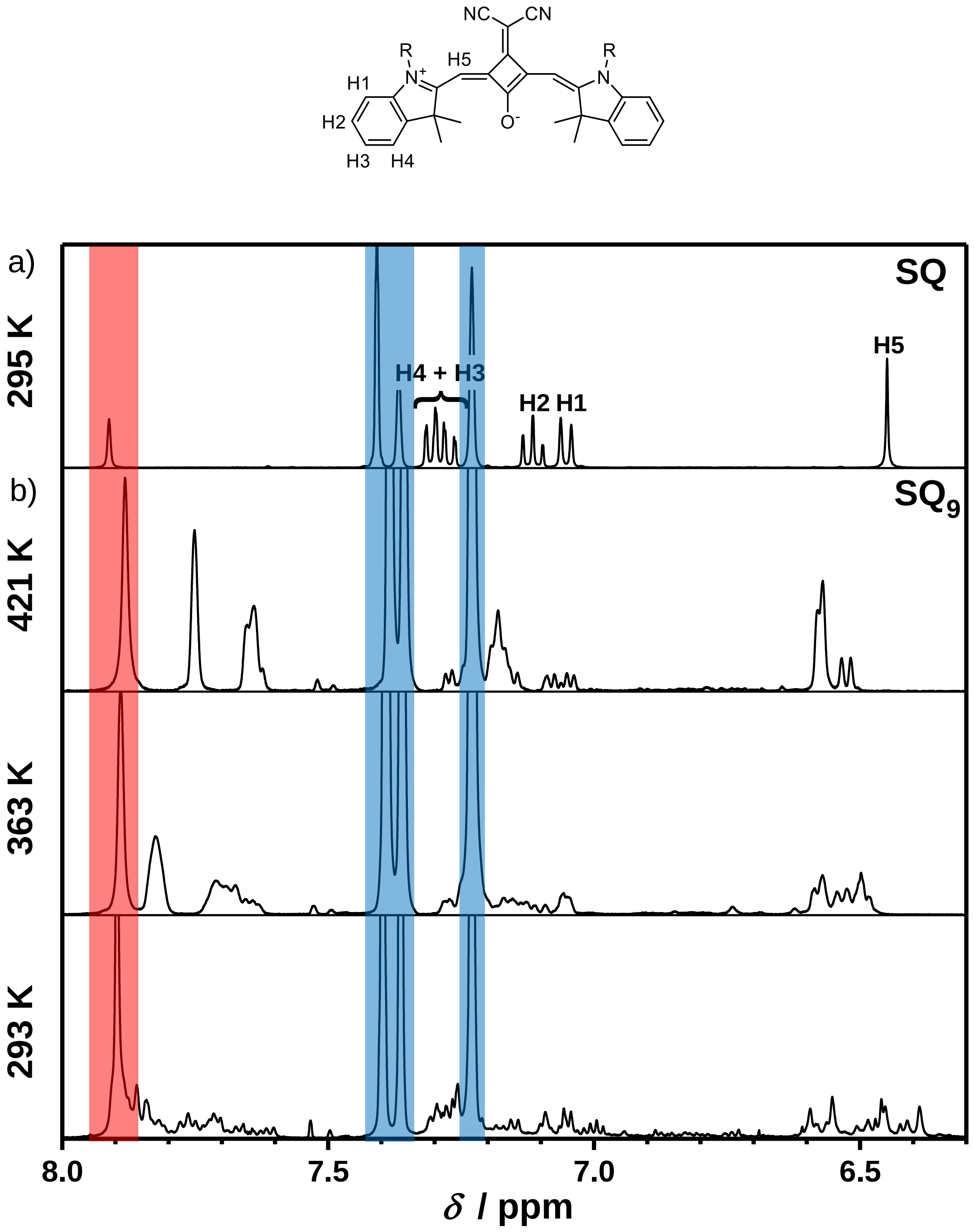

Figure S46. a) ^{1}H NMR spectrum of **SQ** in PhCN-d_5 at 295 K and b) ^{1}H NMR spectra of **SQ$_9$** in PhCN-d_5 at a concentration of ca. 6.58×10^{-4} M. Peaks of PhCN are marked in blue and an unknown contamination in the solvent is marked in red.

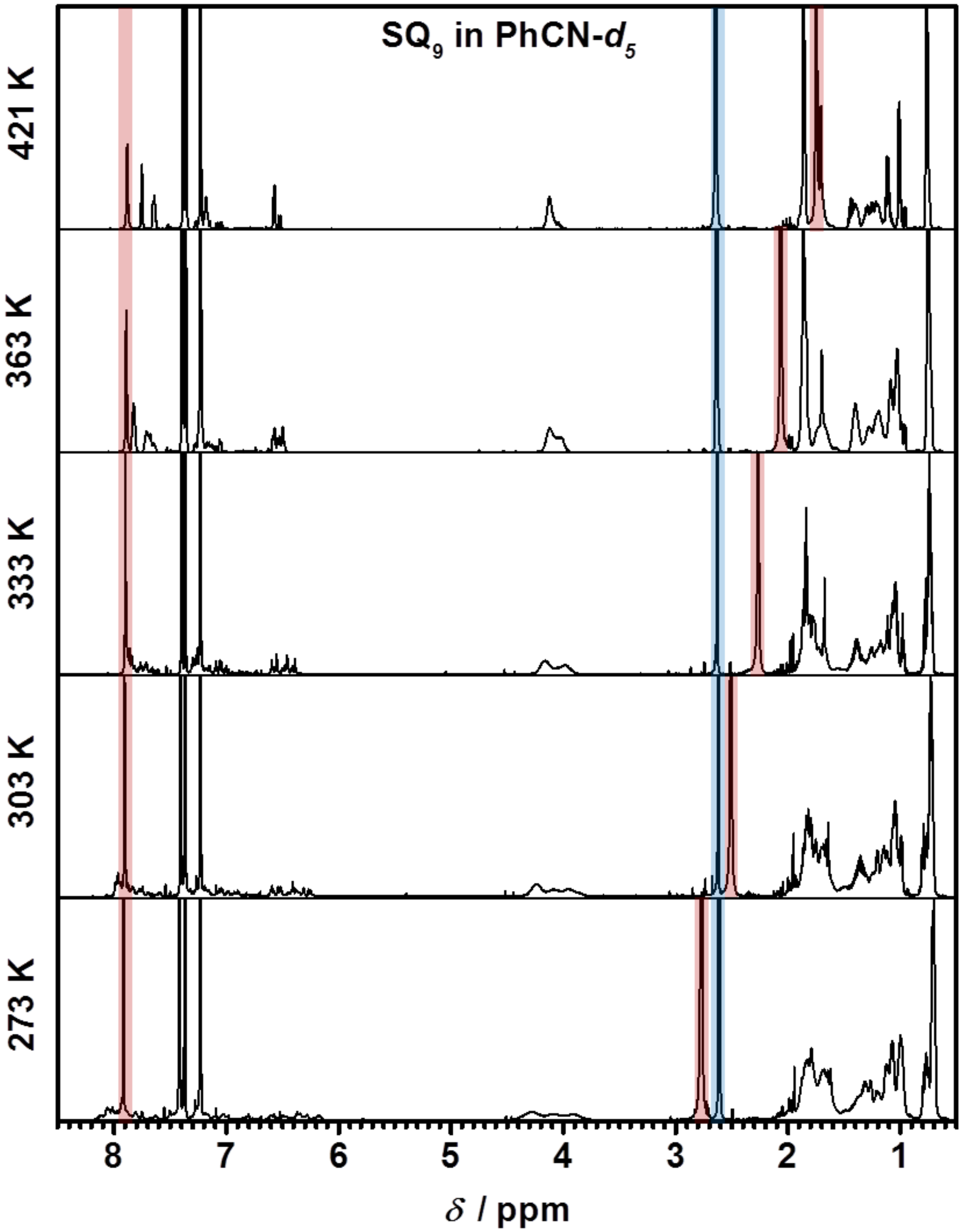

Figure S47. ^{1}H NMR spectra of **SQ9** at selected temperatures in PhCN-*d*5. Marked in blue: water at 2.6 ppm (273 K), and an unknown contamination marked in red at 2.8 ppm (273 K) that is shifting towards lower ppm with increasing temperature.

DOSY NMR

The DOSY NMR spectra were handled with the methode described above. The recorded spectra in CD_2Cl_2 (a) as well as in acetone-d_6 (b) are displayed below. The concentration of 1.29×10^{-3} M was kept throughout all experiments.

a) b)

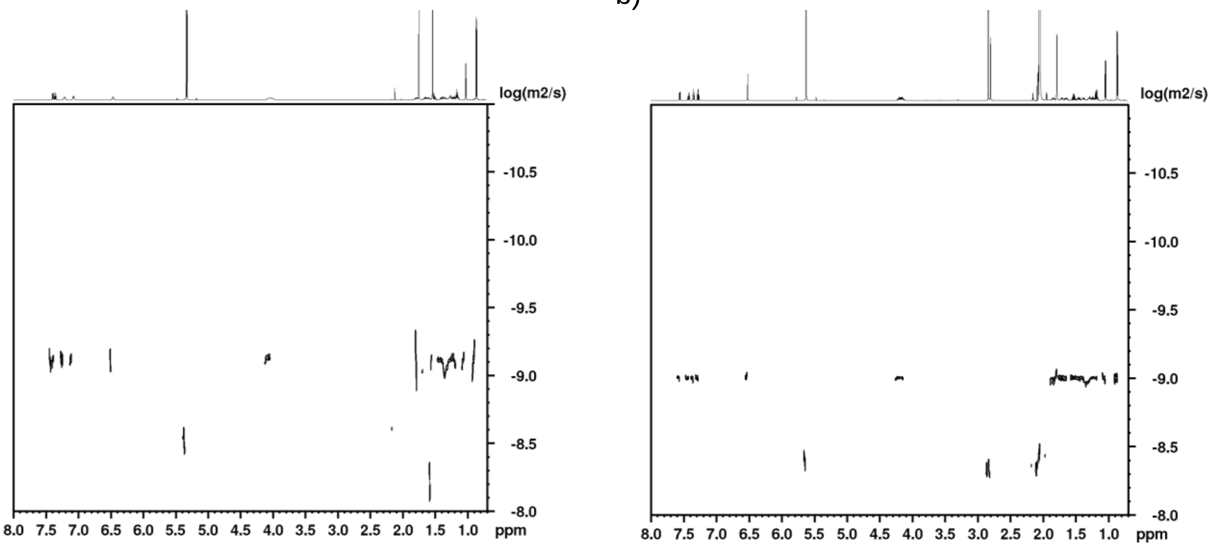

Figure S48. DOSY NMR spectra of **SQ** in (a) CD_2Cl_2 and (b) acetone-d_6.

a) b)

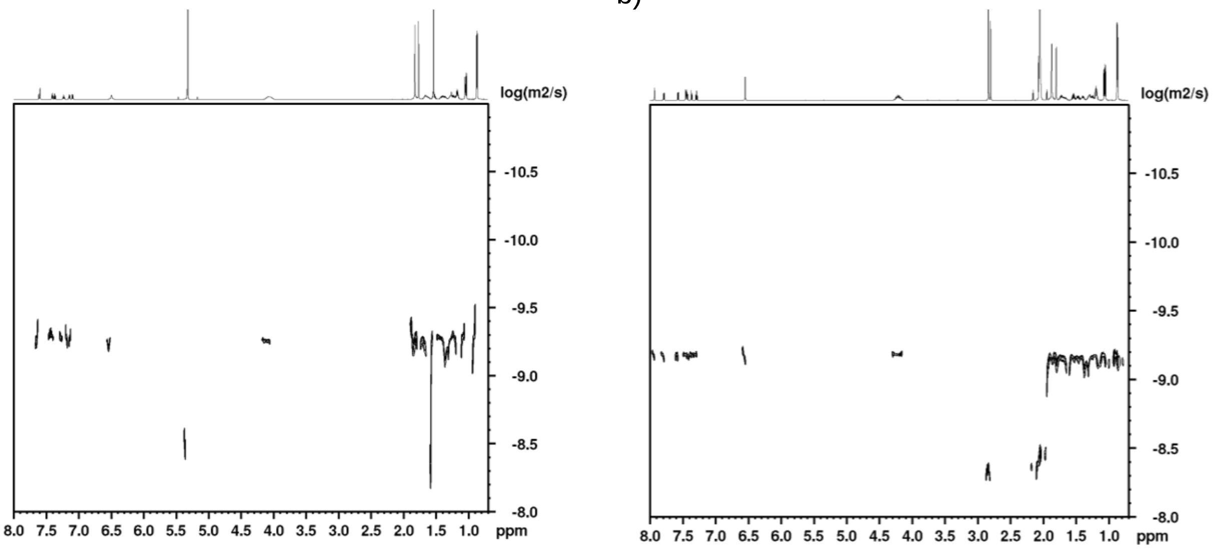

Figure S49. DOSY NMR spectra of $\mathbf{SQ_2}$ in (a) CD_2Cl_2 and (b) acetone-d_6.

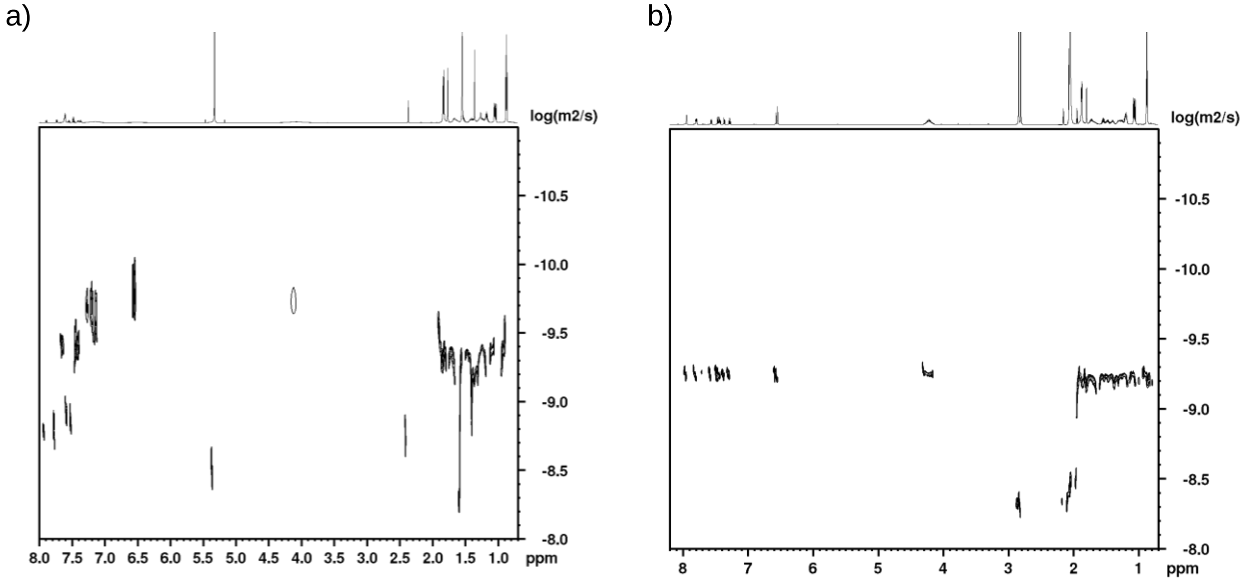

Figure S50. DOSY NMR spectra of **SQ_3** in (a) CD_2Cl_2 and (b) acetone-d_6.

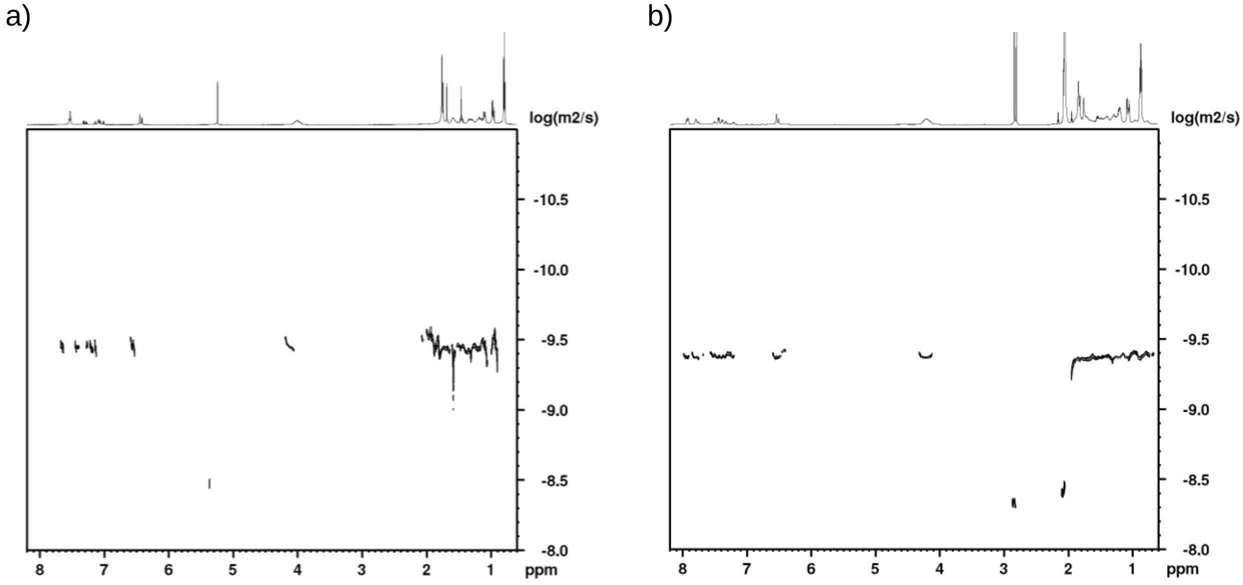

Figure S51. DOSY NMR spectra of **SQ_4** in (a) CD_2Cl_2 and (b) acetone-d_6.

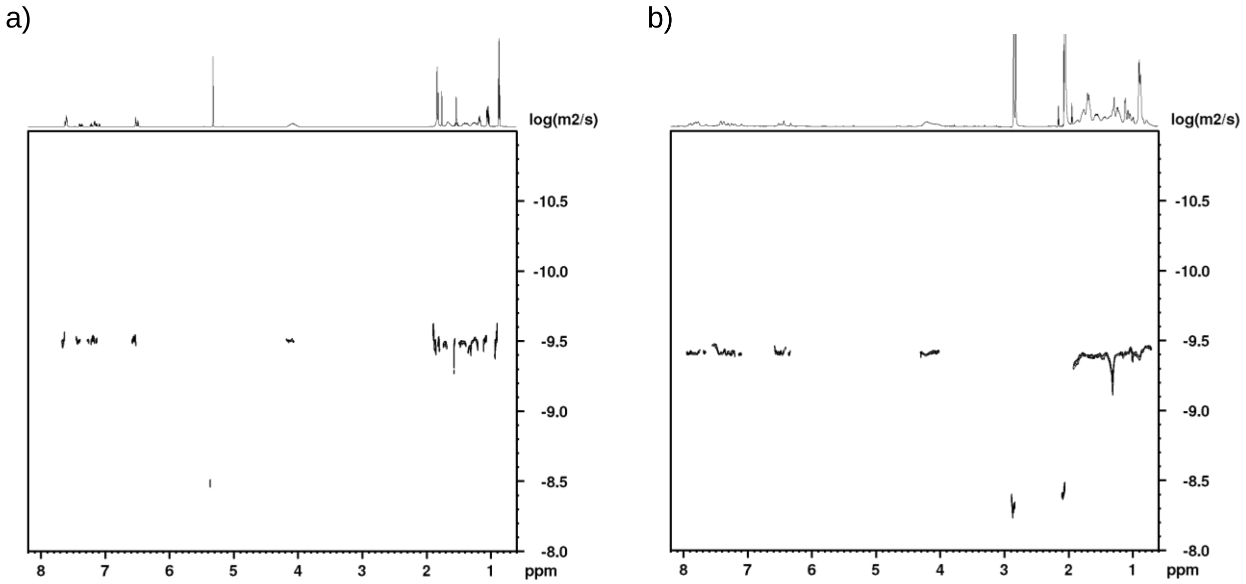

Figure S52. DOSY NMR spectra of **SQ_5** in (a) CD_2Cl_2 and (b) acetone-d_6.

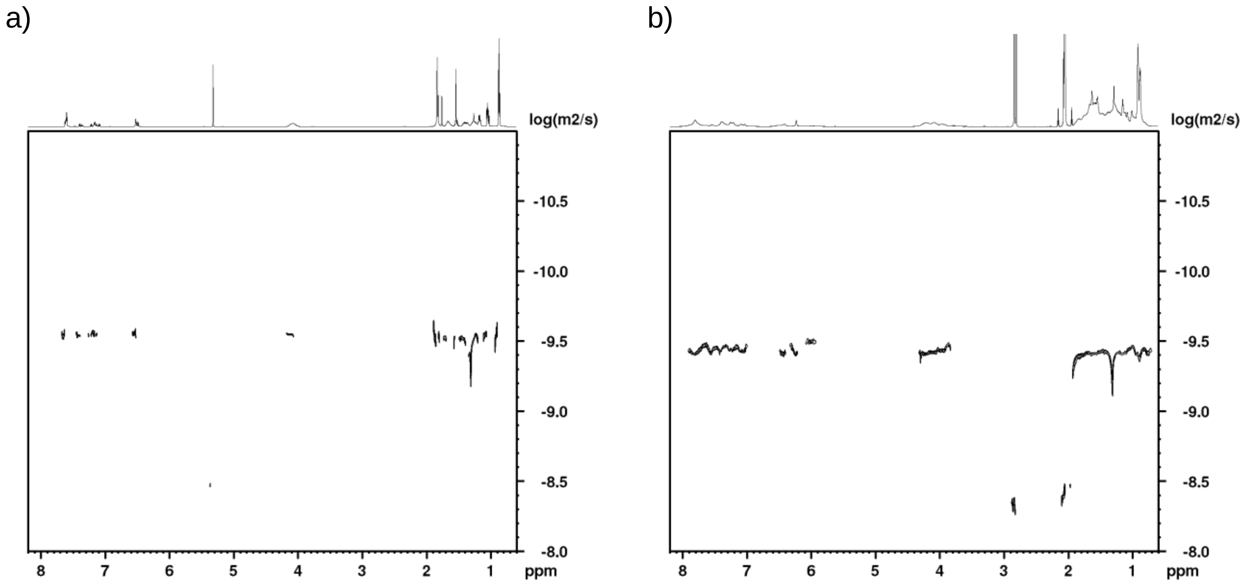

Figure S53. DOSY NMR spectra of **SQ_6** in (a) CD_2Cl_2 and (b) acetone-d_6.

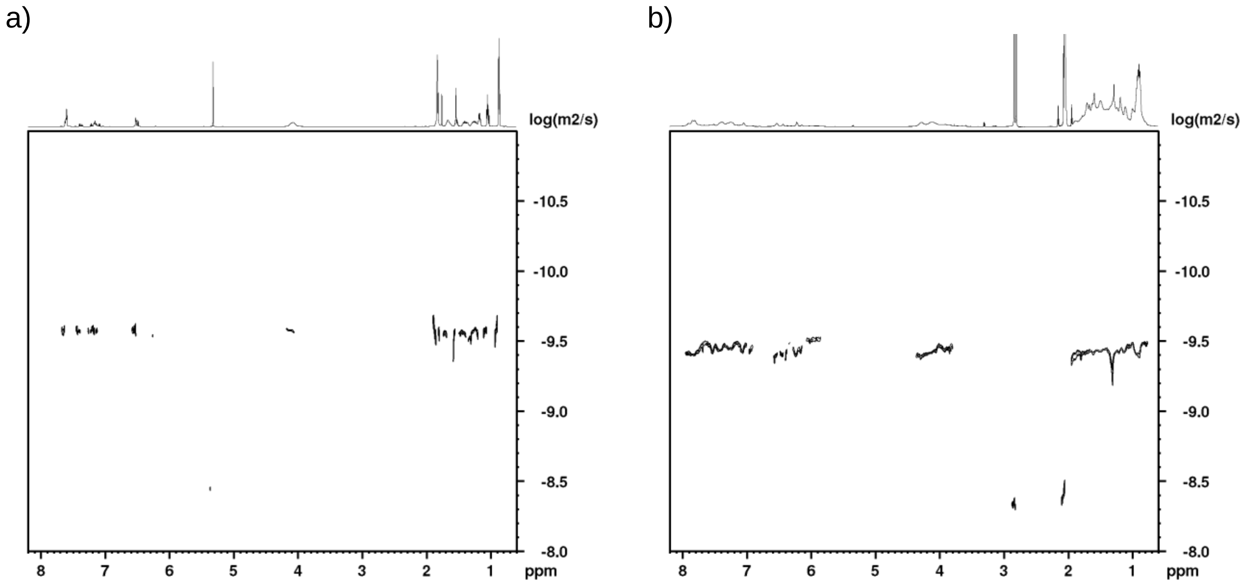

Figure S54. DOSY NMR spectra of **SQ_7** in (a) CD_2Cl_2 and (b) acetone-d_6.

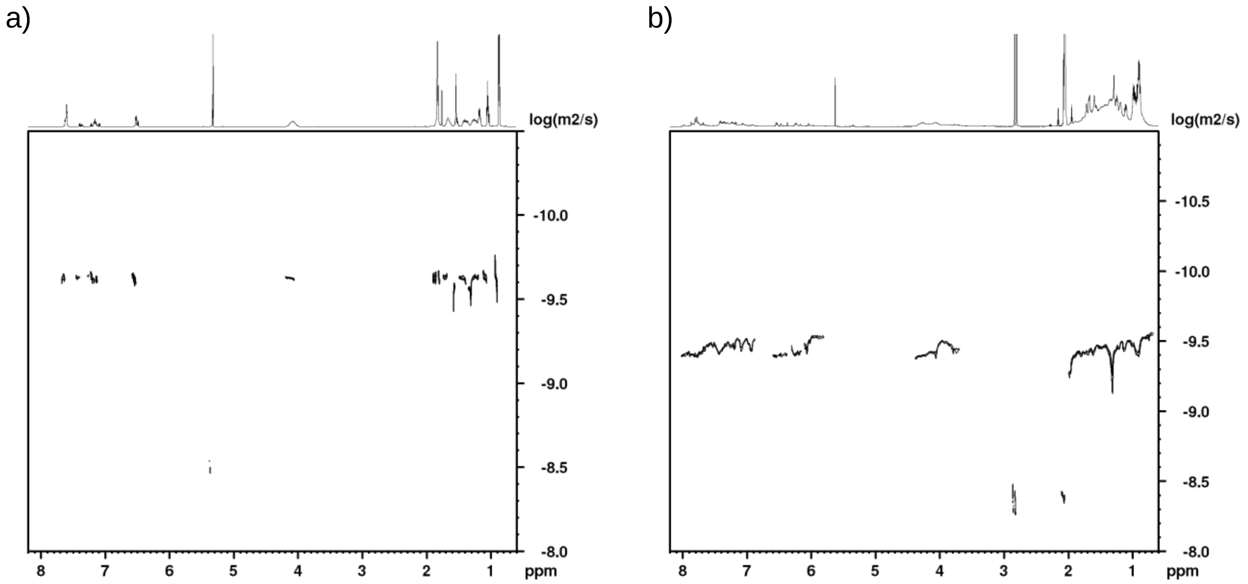

Figure S55. DOSY NMR spectra of **SQ_8** in (a) CD_2Cl_2 and (b) acetone-d_6.

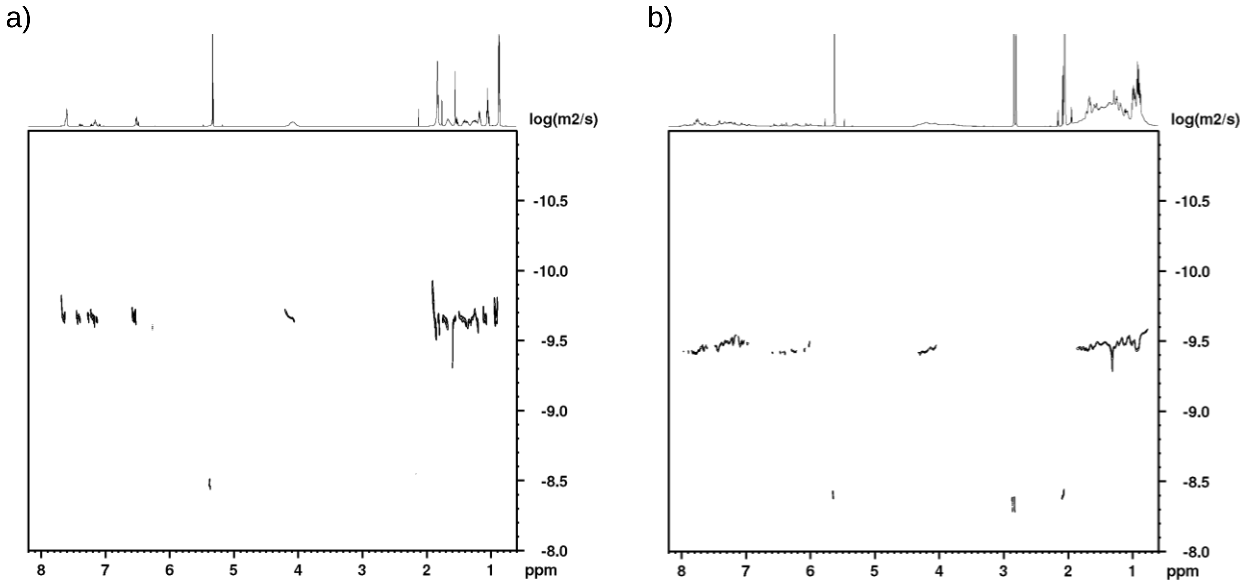

Figure S56. DOSY NMR spectra of **SQ$_9$** in (a) CD_2Cl_2 and (b) acetone-d_6.

5 Small Angle Neutron Scattering (SANS)

The measurement was performed at the beamline D11 at Institut Laue Langevin (ILL), Grenoble, France.[9] A sample of the octamer **SQ$_8$** with a concentration of c = 1 g L^{-1} (=1.7 x 10^{-4} M) was prepared in acetone-d_6 and transferred into a Hellma 120-QS quartz cell with 5 mm path length. Two configurations with a neutron wavelength of λ = 6 Å (FWHM 9%) and sample detector distances of d = 1.4 m and d = 8 m were used. The collimation distance was 8.0 m for both the sample detector distances and the employed neutron beam was 15 mm in diameter. A total scattering vector range of 0.1 nm^{-1} $\leq q \leq$ 4.5 nm^{-1} was investigated. Scattered neutrons were detected using a ^{3}He gas detector (CERCA) consisting of 256 x 256 pixels of 3.75 mm $\times$ 3.75 mm size. Data were put on an absolute scale by measuring a 1 mm H_2O cuvette as secondary calibration standard, with the known differential scattering cross-section of 0.983 cm^{-1}.

The SANS intensity after incoherent background subtraction (see below) is

$$I(q) = \phi V_{NP} (\Delta\rho_{SLD})^2 P(q) S(q) \qquad \text{S8}$$

where ϕ is the volume fraction, V_{NP} the particle volume, $\Delta\rho_{SLD}$ the difference in scattering length density between the solute and the solvent and $P(q)$ the particle form factor characteristic of the particle shape. The structure factor $S(q)$ determined by interparticle correlations is approximated as $S(q)$ = 1, as the sample has been diluted such that no interparticle correlations are expected to play a role in the investigated q-range. SasView 4.2.2 is used to fit the scattering data to

model form factors.[10] The incoherent background scattering is determined as part of this fitting procedure.

From a Guinier plot, i.e. ln(*I*) *vs.* q^2 for low q (see Figure S57) the radius of gyration R_G can be determined. Figure S57 shows a linear behavior in the Guinier region for $q\ R_G \leq 1.17$ with a resulting radius of gyration R_G = (1.56 ± 0.02) nm.

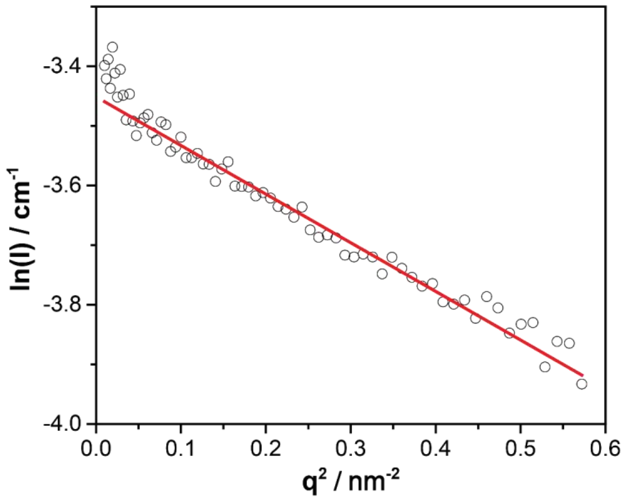

3

Figure S57. SANS of **SQ$_8$** in acetone-d_6: Guinier plot. Error bars lie within the symbol size. The slope of the linear dependency in the low q-region up to q_{max} = 0.75 nm^{-1} yields the radius of gyration R_G = (1.56 ± 0.02) nm.

Figure S58 shows the complete SANS scattering curve of **SQ$_8$** after background subtraction as a log-log plot. Data could be fitted with the form factor of a monodisperse flexible cylinder,[10-11] as seen from the red line in Figure S58. The fitting yields a cross-section radius R_c = (0.9 ± 0.1) nm, a total length (contour length) of L = (8.5 ± 0.5) nm, and a Kuhn segment length of l_K = (2.1 ± 0.1) nm. Based on form factor fitting and scaling behavior, the presence of a Gauss coil, excluded volume coil and a stiff cylinder could excluded.

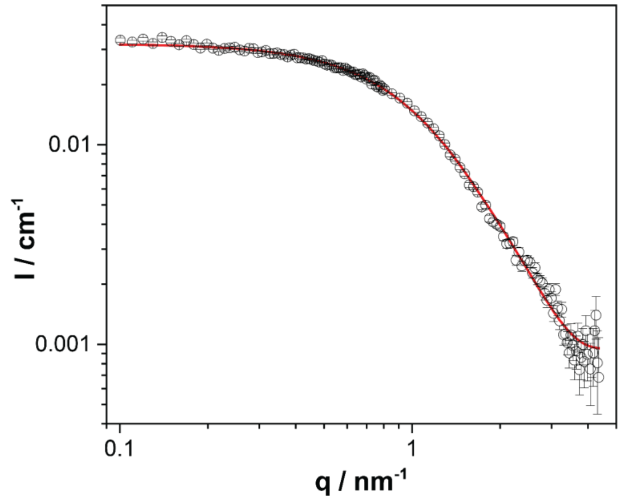

Figure S58. SANS of **SQ₈** in acetone-d_6: data points (open symbols) and flexible cylinder model fit. (red line, reduced χ^2 = 2.305.) The fit reveals a cross-section radius R_c = (0.9 ± 0.1) nm, a total length (contour length) L = (8.5 ± 0.5) nm and a Kuhn segment length l_K = (2.1 ± 0.1) nm.

6 Calculations

Semiempirical Calculations

The structures of **SQ₈** were optimised with the AM1 hamiltonian using MOPAC2016.[12] Long alkyl chains were replaced by methyl groups. Using these optimised geometries, we computed the transition energies and oscillator strengths by ZINDO using Gaussian09.[13]

Table S4. Cartesian coordinates of AM1 optimised **SQ₈** helix

C	0	0.000000	0.000000	0.000000	C	0	-9.137047	7.568064	34.804319
C	0	0.000000	1.399246	0.000000	C	0	-9.285912	6.240012	35.311546
C	0	1.187484	2.137232	0.000000	C	0	-8.224495	5.361761	35.306800
C	0	2.393688	1.437487	-0.004858	N	0	-10.358993	8.260422	34.923104
C	0	2.402730	0.008471	-0.009308	C	0	-11.335021	7.442139	35.509525
C	0	1.222633	-0.702733	-0.003513	C	0	-10.710598	6.059266	35.798228
N	0	3.719761	1.919351	-0.009599	C	0	-10.715541	5.758788	37.284592
C	0	4.630557	0.856449	-0.010879	C	0	-12.609141	7.910159	35.744345
C	0	3.841003	-0.471324	-0.027371	C	0	-13.741145	7.327736	36.301642
C	0	4.108766	-1.243896	-1.305122	C	0	-15.047016	7.945119	36.527043
C	0	5.991386	1.083277	0.007440	C	0	-15.538388	6.689017	37.107126
C	0	7.088069	0.233294	0.008385	C	0	-14.215675	6.050782	36.873662
C	0	8.504704	0.596794	0.066087	C	0	-16.792498	6.426679	37.622634
C	0	8.868868	-0.822340	0.000661	O	0	-13.737318	4.933596	37.072653
C	0	7.435409	-1.204078	-0.052251	C	0	-17.358464	5.294373	38.188022
C	0	10.137510	-1.374219	0.003445	N	0	-18.675882	5.296666	38.638922
O	0	6.810950	-2.263547	-0.115937	C	0	-19.024569	4.031572	39.170207
C	0	10.594801	-2.677092	-0.088028	C	0	-17.904414	3.150535	39.073936
N	0	11.959533	-2.965966	-0.056893	C	0	-16.748737	3.895855	38.436498
C	0	12.177936	-4.355877	-0.191934	C	0	-20.228498	3.597944	39.722901
C	0	10.922893	-5.027317	-0.311558	C	0	-20.298231	2.275104	40.177171
C	0	9.810091	-3.999828	-0.240555	C	0	-19.207710	1.415285	40.084076
C	0	13.371747	-5.075037	-0.217683	C	0	-17.995852	1.850383	39.528272

C	0	13.297895	-6.463576	-0.364466
C	0	12.073144	-7.129799	-0.486460
C	0	10.868888	-6.396491	-0.459160
C	0	13.000486	-1.993411	0.071809
C	0	4.079293	3.302857	0.013055
C	0	9.201203	1.768714	0.148797
C	0	8.946527	-4.259035	0.979313
C	0	3.399921	-14.183151	-10.161736
C	0	3.335741	-15.560649	-10.350334
C	0	4.110692	-16.441228	-9.585373
C	0	4.955155	-15.892742	-8.621521
C	0	5.022458	-14.479305	-8.426811
C	0	4.249316	-13.630060	-9.192469
N	0	5.837299	-16.535028	-7.719594
C	0	6.494743	-15.599621	-6.924649
C	0	6.017830	-14.184085	-7.322475
C	0	5.319208	-13.502076	-6.162095
C	0	7.410060	-15.995036	-5.961455
C	0	8.182576	-15.290917	-5.058671
C	0	9.132250	-15.825777	-4.075064
C	0	9.466740	-14.464178	-3.660899
C	0	8.498951	-13.903729	-4.626653
C	0	10.399929	-14.068677	-2.710303
O	0	8.105575	-12.779698	-4.940145
C	0	10.787178	-12.834458	-2.236336
N	0	11.777061	-12.714695	-1.250732
C	0	11.989907	-11.360332	-0.923155
C	0	11.125439	-10.534784	-1.706873
C	0	10.293540	-11.420456	-2.613964
C	0	12.871522	-10.789424	-0.005479
C	0	12.880633	-9.396987	0.118402
C	0	12.035984	-8.581901	-0.643266
C	0	11.144986	-9.164212	-1.568448
C	0	12.463238	-13.813272	-0.646636
C	0	6.019014	-17.951955	-7.640383
C	0	9.525797	-17.078648	-3.699430
C	0	10.603763	-11.107094	-4.065225
C	0	-1.260876	-0.738305	0.001175
C	0	-2.318387	-0.331043	-0.819828
C	0	-3.535631	-1.018308	-0.850818
C	0	-3.677533	-2.135125	-0.028535
C	0	-2.605899	-2.553938	0.818429
C	0	-1.411004	-1.867540	0.832391
N	0	-4.779199	-3.003314	0.138073
C	0	-4.479747	-4.006494	1.063085
C	0	-3.049961	-3.774210	1.601334
C	0	-3.078601	-3.449957	3.083267
C	0	-5.391524	-5.002931	1.356426
C	0	-5.352644	-6.089327	2.214704
C	0	-6.365384	-7.134158	2.387714
C	0	-5.524889	-7.777463	3.400051
C	0	-4.495221	-6.723391	3.244962
C	0	-5.780113	-8.948177	4.096718
O	0	-3.401687	-6.476516	3.754388
C	0	-5.103579	-9.604684	5.106038
N	0	-5.589301	-10.801206	5.641845
C	0	-4.757754	-11.249867	6.690930
C	0	-3.669423	-10.339956	6.862155
C	0	-3.796131	-9.228449	5.838535
C	0	-4.870324	-12.379905	7.499826
C	0	-3.891353	-12.588312	8.475993
C	0	-2.826517	-11.698435	8.655518
C	0	-2.717300	-10.557309	7.834441
C	0	-6.789450	-11.456568	5.223674
C	0	-6.012711	-2.891088	-0.576774
C	0	-19.562548	6.417915	38.578101
C	0	-10.566176	9.615714	34.519527
C	0	-15.591529	9.176264	36.295927
C	0	-16.362127	3.233465	37.128267
C	0	0.016026	-2.068714	34.722281
N	0	0.785749	-1.287572	35.117354
C	0	-0.336912	-4.351422	34.135456
N	0	0.149220	-5.407472	34.051723
C	0	-14.861957	10.235872	35.706301
N	0	-14.294068	11.132334	35.224160
C	0	-16.930302	9.494511	36.626184
N	0	-18.027124	9.794153	36.883173
C	0	-15.586104	3.977094	39.406894
C	0	-11.399370	4.975179	34.991704
C	0	-5.731777	0.388050	34.974227
C	0	-5.617752	-4.406299	30.929140
H	0	-0.959825	1.940036	0.008453
H	0	1.152092	3.235175	0.005153
H	0	1.230354	-1.803238	-0.010591
H	0	3.473074	-2.162461	-1.319673
H	0	5.180820	-1.550062	-1.361775
H	0	3.860501	-0.621598	-2.198009
H	0	6.299392	2.152786	0.028887
H	0	10.928556	-0.596254	0.094501
H	0	14.350020	-4.583077	-0.129014
H	0	14.234120	-7.043721	-0.392795
H	0	9.901525	-6.914075	-0.546577
H	0	13.999389	-2.502712	0.075975
H	0	12.962357	-1.268574	-0.787529
H	0	12.877031	-1.421602	1.032453
H	0	3.154824	3.937303	0.011229
H	0	4.679507	3.533108	0.936139
H	0	4.698081	3.555166	-0.891647
H	0	8.446682	-5.252314	0.870938
H	0	9.569920	-4.270371	1.905268
H	0	8.160576	-3.472364	1.078856
H	0	2.778701	-13.516512	-10.778098
H	0	2.662710	-15.975884	-11.116512
H	0	4.042829	-17.524647	-9.752638
H	0	4.298135	-12.541051	-9.045618
H	0	4.919736	-12.515901	-6.502890
H	0	6.033325	-13.327004	-5.321777
H	0	4.469440	-14.125724	-5.794615
H	0	7.571096	-17.093596	-5.880927
H	0	10.921135	-14.942705	-2.259181
H	0	13.544877	-11.400338	0.610974
H	0	13.568796	-8.934625	0.843939
H	0	10.482455	-8.529614	-2.176330
H	0	13.021460	-14.397320	-1.429178
H	0	13.193065	-13.434705	0.115724
H	0	11.728674	-14.498931	-0.141063
H	0	5.359864	-18.463122	-8.389645
H	0	7.091024	-18.216397	-7.854841
H	0	5.755469	-18.318610	-6.610158
H	0	10.307533	-10.052722	-4.285804
H	0	11.695842	-11.222237	-4.265729
H	0	10.035615	-11.787422	-4.743862
H	0	-2.190629	0.550067	-1.468798
H	0	-4.343718	-0.675292	-1.511259
H	0	-0.586591	-2.188220	1.487217
H	0	-2.040223	-3.228834	3.431037
H	0	-3.474848	-4.314899	3.667428
H	0	-3.719472	-2.556500	3.276047
H	0	-6.354630	-4.948998	0.800695
H	0	-6.725913	-9.430239	3.761399

C	0	-7.587297	-7.396725	1.836885	H	0	-5.696992	-13.094865	7.389080
C	0	-2.614518	-9.272261	4.887561	H	0	-3.969909	-13.475194	9.124911
C	0	10.615232	1.817125	0.179606	H	0	-1.877926	-9.857683	7.965568
N	0	11.777801	1.897625	0.206042	H	0	-6.723079	-11.719458	4.132165
C	0	8.566350	3.031979	0.211541	H	0	-6.937130	-12.396503	5.816875
N	0	8.079983	4.089821	0.266693	H	0	-7.676188	-10.782013	5.379184
C	0	10.481853	-17.305623	-2.681033	H	0	-6.180741	-3.806342	-1.208842
N	0	11.263919	-17.532473	-1.847060	H	0	-6.868161	-2.789770	0.146330
C	0	9.006558	-18.253430	-4.293463	H	0	-5.987556	-1.988032	-1.240895
N	0	8.601767	-19.243176	-4.757570	H	0	-1.677420	-9.054944	5.455598
C	0	-8.379735	-8.501957	2.228365	H	0	-2.525147	-10.282409	4.420901
N	0	-9.061584	-9.398968	2.526907	H	0	-2.729668	-8.508217	4.081813
C	0	-8.162499	-6.584532	0.830856	H	0	-14.426714	1.445564	18.540052
N	0	-8.667088	-5.941382	-0.000084	H	0	-13.218213	3.516754	17.917472
C	0	-12.641407	0.254478	18.823456	H	0	-10.687962	-0.697349	19.029853
C	0	-13.325545	1.433365	18.505846	H	0	-8.587330	-0.299325	17.476201
C	0	-12.650913	2.606643	18.155724	H	0	-7.405494	1.008529	17.044062
C	0	-11.257411	2.575594	18.125202	H	0	-9.036871	0.954952	16.243133
C	0	-10.550358	1.374892	18.441406	H	0	-8.204733	5.026594	17.469960
C	0	-11.231982	0.228581	18.789219	H	0	-2.831840	4.954018	17.694517
N	0	-10.336419	3.598992	17.811626	H	0	2.118059	3.202972	17.893525
C	0	-9.024268	3.131331	17.923790	H	0	3.246718	1.000305	18.018056
C	0	-9.062226	1.635606	18.310142	H	0	-0.571425	-1.053687	18.113127
C	0	-8.487016	0.778080	17.198356	H	0	-0.753898	5.374500	18.633113
C	0	-7.947537	3.971649	17.715803	H	0	0.777389	4.839681	17.789498
C	0	-6.576974	3.774382	17.760429	H	0	-0.717403	5.229583	16.810982
C	0	-5.521458	4.778288	17.603658	H	0	-10.280790	5.212939	16.469702
C	0	-4.507795	3.735500	17.780247	H	0	-11.817800	5.035204	17.443328
C	0	-5.563462	2.706566	17.933466	H	0	-10.295305	5.657392	18.243064
C	0	-3.130431	3.885715	17.789900	H	0	-2.677198	-0.104808	19.392457
O	0	-5.588134	1.489655	18.120084	H	0	-2.196921	1.462494	20.172625
C	0	-2.087904	2.984488	17.885572	H	0	-3.810653	1.311934	19.348649
N	0	-0.758543	3.416128	17.875706	H	0	-1.652247	-14.054217	9.225313
C	0	0.122364	2.314419	17.936313	H	0	0.049096	-14.527948	10.963584
C	0	-0.630437	1.101632	18.000977	H	0	-1.795898	-9.875280	10.375487
C	0	-2.107928	1.443449	17.998124	H	0	-1.594937	-9.063052	12.877781
C	0	1.516279	2.285233	17.943264	H	0	-0.410467	-9.068313	14.252694
C	0	2.145630	1.039054	18.014745	H	0	-1.427883	-10.538259	13.922709
C	0	1.416553	-0.154113	18.071571	H	0	2.666999	-11.774966	14.598945
C	0	0.007120	-0.118867	18.062595	H	0	4.424357	-7.652949	17.578922
C	0	-0.342122	4.780251	17.771583	H	0	4.751567	-2.895478	19.789323
C	0	-10.701040	4.940685	17.476770	H	0	3.524746	-0.748001	19.633904
C	0	-5.493882	6.125879	17.383357	H	0	0.836883	-2.393456	16.654985
C	0	-2.737225	1.007570	19.308228	H	0	5.919960	-6.020560	18.253662
C	0	-1.825581	-11.947515	9.690543	H	0	5.505332	-4.868099	19.611213
C	0	-1.304816	-13.233418	9.872938	H	0	4.774191	-6.543837	19.578823
C	0	-0.343784	-13.508288	10.850491	H	0	1.592262	-13.794153	14.220770
C	0	0.087885	-12.456621	11.657721	H	0	1.472551	-14.491650	12.534997
C	0	-0.443535	-11.141815	11.482998	H	0	2.880815	-13.413623	12.980930
C	0	-1.385077	-10.887463	10.509380	H	0	1.605970	-4.083471	14.792579
N	0	1.031225	-12.450105	12.708293	H	0	3.390184	-4.353443	14.991530
C	0	1.163741	-11.165341	13.242934	H	0	2.292643	-5.754489	14.620322
C	0	0.191410	-10.218387	12.504453	H	0	-14.860448	-0.696020	17.629488
C	0	-0.871909	-9.693084	13.450785	H	0	-16.167872	-2.713538	18.227480
C	0	2.063592	-10.903833	14.257809	H	0	-12.049227	-1.454006	20.844044
C	0	2.386933	-9.750178	14.954157	H	0	-12.707364	-2.437921	23.157304
C	0	3.420963	-9.580191	15.977869	H	0	-13.409587	-3.937205	23.900592
C	0	3.029948	-8.176835	16.135455	H	0	-14.507477	-2.669212	23.198284
C	0	1.984074	-8.331187	15.096395	H	0	-15.916022	-6.489130	21.877344
C	0	3.574793	-7.235246	16.993114	H	0	-14.206082	-9.471477	26.013778
O	0	1.144976	-7.596046	14.574882	H	0	-11.246337	-11.261833	29.973991
C	0	3.267632	-5.913357	17.253346	H	0	-9.165292	-10.566461	31.124856
N	0	3.999205	-5.172990	18.185742	H	0	-8.790527	-7.099325	28.546326
C	0	3.482729	-3.863465	18.296858	H	0	-13.308159	-11.397639	26.956557
C	0	2.371957	-3.709753	17.411389	H	0	-12.964774	-11.478877	28.750762

C	0	2.160828	-5.011299	16.662379	H	0	-14.298713	-10.397305	28.122929
C	0	3.902376	-2.802744	19.098553	H	0	-16.983771	-4.479671	19.069606
C	0	3.205943	-1.594249	19.004528	H	0	-16.343864	-6.097168	19.632629
C	0	2.114511	-1.436030	18.143033	H	0	-17.361014	-5.075440	20.756782
C	0	1.691627	-2.513286	17.337789	H	0	-9.434828	-7.099160	26.019512
C	0	5.102639	-5.674755	18.944652	H	0	-9.996861	-8.764115	25.563985
C	0	1.780330	-13.592761	13.130357	H	0	-10.879362	-7.301901	24.940033
C	0	4.361942	-10.391329	16.545037	H	0	-12.272894	-5.891499	26.592325
C	0	2.380174	-4.790960	15.177486	H	0	-12.338083	-6.395919	28.338239
C	0	-13.384535	-0.948201	19.192907	H	0	-10.805641	-5.712435	27.644865
C	0	-14.531838	-1.314380	18.480095	H	0	-11.982042	-5.374371	22.297770
C	0	-15.274015	-2.452206	18.810204	H	0	-12.095880	-5.065198	20.509621
C	0	-14.841832	-3.225926	19.886608	H	0	-11.295689	-3.849020	21.594597
C	0	-13.671033	-2.860335	20.619531	H	0	-8.795509	2.053090	20.429201
C	0	-12.948291	-1.738172	20.276443	H	0	-8.507235	0.329519	19.937150
N	0	-15.395425	-4.403134	20.434917	H	0	-7.272273	1.599540	19.544446
C	0	-14.638883	-4.841479	21.525899	H	0	-2.694096	-0.293823	16.858172
C	0	-13.450979	-3.873771	21.725906	H	0	-3.877877	1.079710	16.788595
C	0	-13.528319	-3.191554	23.078014	H	0	-2.322285	1.169430	15.849946
C	0	-14.998654	-5.970258	22.235868	H	0	0.008519	-4.849038	16.609326
C	0	-14.443375	-6.608688	23.333627	H	0	0.633828	-6.543133	16.432420
C	0	-14.933100	-7.808545	24.016210	H	0	0.655030	-5.730018	18.059174
C	0	-13.783444	-7.758061	24.923651	H	0	1.724506	-9.516059	11.129228
C	0	-13.287719	-6.529646	24.257434	H	0	0.223348	-8.497520	11.201011
C	0	-13.462992	-8.646157	25.936945	H	0	1.427053	-8.429412	12.557054
O	0	-12.333016	-5.767556	24.412993	H	0	-4.035378	-7.067736	5.780182
C	0	-12.422614	-8.698130	26.845444	H	0	-4.809206	-7.878978	7.212346
N	0	-12.334845	-9.729294	27.783850	H	0	-3.004722	-7.694601	7.135553
C	0	-11.194116	-9.563766	28.600165	H	0	-2.485255	-5.859853	1.850161
C	0	-10.489900	-8.385263	28.204859	H	0	-2.160599	-5.177325	0.195846
C	0	-11.223687	-7.743602	27.043413	H	0	-1.104562	-4.710368	1.596821
C	0	-10.722812	-10.352471	29.648710	H	0	3.923900	-0.700439	2.134744
C	0	-9.546885	-9.954510	30.291944	H	0	3.480783	-2.201136	1.213962
C	0	-8.851682	-8.800750	29.912783	H	0	5.199939	-1.621000	1.223376
C	0	-9.335166	-8.004962	28.853719	H	0	9.668915	-3.818780	-2.404471
C	0	-13.271229	-10.802773	27.910430	H	0	8.526392	-5.011655	-1.650878
C	0	-16.578170	-5.043749	19.949641	H	0	8.206890	-3.232423	-1.495598
C	0	-16.016952	-8.627426	23.874781	H	0	8.514027	-10.201600	-2.503183
C	0	-10.333978	-7.729998	25.815093	H	0	8.203436	-11.939093	-2.924268
C	0	-6.669119	6.906385	17.271769	H	0	8.626411	-11.478378	-1.217239
N	0	-7.613856	7.582561	17.177214	H	0	7.652454	-13.881424	-8.726828
C	0	-4.287634	6.853508	17.247823	H	0	6.775826	-12.373936	-8.222074
N	0	-3.315864	7.485942	17.127867	H	0	7.930648	-13.174891	-7.074403
C	0	4.529706	-11.745541	16.169623	H	0	-4.443842	6.385530	35.479675
N	0	4.699712	-12.863108	15.885375	H	0	-2.482672	4.871647	35.518036
C	0	5.249065	-9.944549	17.553137	H	0	-7.013037	3.170931	34.110114
N	0	5.995258	-9.614073	18.385389	H	0	-6.213886	1.295734	32.422786
C	0	-17.005544	-8.422525	22.883048	H	0	-4.996186	-0.045208	32.309382
N	0	-17.840206	-8.285769	22.080990	H	0	-4.494219	1.670398	31.977414
C	0	-16.244410	-9.745691	24.711787	H	0	-1.564261	-0.217714	34.565743
N	0	-16.466441	-10.673762	25.381201	H	0	-2.447522	-5.426488	33.530402
C	0	-11.693925	-6.354220	27.427547	H	0	-4.740473	-9.838666	31.816055
C	0	-12.129595	-4.588548	21.518446	H	0	-6.846645	-10.438083	30.657504
C	0	-8.366612	1.396030	19.635343	H	0	-8.153828	-6.303021	30.701722
C	0	-2.792250	0.817569	16.799250	H	0	-2.815037	-7.649723	34.079237
C	0	0.784409	-5.571493	16.961861	H	0	-3.096200	-8.904509	32.780015
C	0	0.941784	-9.100611	11.807973	H	0	-2.084656	-7.424281	32.418636
C	0	-3.924768	-7.886587	6.531436	H	0	-0.688017	1.896042	34.267774
C	0	-2.150756	-4.953935	1.289445	H	0	-1.099745	3.268669	35.403198
C	0	4.132468	-1.293690	1.212481	H	0	-1.106352	1.544272	36.012542
C	0	9.006954	-4.010721	-1.526408	H	0	-7.675724	-4.670198	32.734079
C	0	8.820741	-11.255176	-2.293554	H	0	-6.766992	-5.615742	33.989552
C	0	7.167225	-13.358727	-7.868090	H	0	-6.275129	-3.909419	33.601887
C	0	-5.841525	4.878759	34.800183	H	0	-5.871337	7.420136	33.902116
C	0	-4.573797	5.332864	35.181478	H	0	-7.770311	9.010655	33.896432

C	0	-3.462805	4.484340	35.207820	H	0	-8.338972	4.341066	35.702088
C	0	-3.647641	3.153720	34.836071	H	0	-10.180490	4.795202	37.467401
C	0	-4.935935	2.682951	34.437274	H	0	-11.761926	5.663608	37.661886
C	0	-6.021070	3.532492	34.420912	H	0	-10.197101	6.569663	37.850041
N	0	-2.716440	2.091977	34.774906	H	0	-12.798268	8.964064	35.440122
C	0	-3.339984	0.914383	34.361560	H	0	-17.462979	7.313197	37.561450
C	0	-4.829690	1.213564	34.078482	H	0	-21.102676	4.257603	39.806940
C	0	-5.147109	1.020084	32.607493	H	0	-21.241316	1.914869	40.616727
C	0	-2.636955	-0.274326	34.273349	H	0	-19.292923	0.381168	40.449518
C	0	-3.002361	-1.554184	33.896320	H	0	-17.135562	1.168801	39.455936
C	0	-2.179513	-2.767923	33.918041	H	0	-19.704299	6.741295	37.510146
C	0	-3.317767	-3.545060	33.426369	H	0	-20.558856	6.139684	39.010712
C	0	-4.156961	-2.327365	33.373510	H	0	-19.136898	7.279166	39.163107
C	0	-3.381588	-4.913949	33.207499	H	0	-9.623836	10.029769	34.074844
O	0	-5.309905	-2.060925	33.032916	H	0	-11.386729	9.668966	33.752071
C	0	-4.354022	-5.730441	32.668713	H	0	-10.859860	10.243877	35.405185
N	0	-4.160852	-7.112840	32.561505	H	0	-16.000321	2.196812	37.334920
C	0	-5.257573	-7.718158	31.912287	H	0	-17.241232	3.176591	36.442663
C	0	-6.234156	-6.724803	31.592939	H	0	-15.544067	3.803653	36.625909
C	0	-5.740600	-5.378276	32.085413	H	0	-14.746836	4.567114	38.966297
C	0	-5.474173	-9.055406	31.581657	H	0	-15.906138	4.456229	40.362998
C	0	-6.665952	-9.385923	30.929765	H	0	-15.219856	2.944904	39.627594
C	0	-7.623399	-8.418342	30.605521	H	0	-12.466619	4.873050	35.303253
C	0	-7.400926	-7.067905	30.945248	H	0	-11.359810	5.213174	33.901810
C	0	-2.982476	-7.806822	32.978285	H	0	-10.881217	4.000953	35.165934
C	0	-1.336288	2.206524	35.132797	H	0	-5.466109	0.540576	36.047597
C	0	-0.884783	-3.051400	34.247623	H	0	-6.792369	0.703059	34.818440
C	0	-6.666094	-4.862492	33.171879	H	0	-5.642445	-0.697129	34.726624
C	0	-6.981314	5.792818	34.799305	H	0	-6.622075	-4.260849	30.461733
C	0	-6.843851	7.094276	34.304323	H	0	-5.244986	-3.416886	31.288676
C	0	-7.911138	7.997132	34.295902	H	0	-4.916127	-4.801472	30.156194

DFT Calculations

Structure optimizations of **SQ** and **SQ$_3$** in the gas phase without symmetry constraints were done by DFT calculations with the B3LYP functional and a 6-31G* basis set using Gaussian09.[13] Here, long alkyl chains were replaced by methyl groups. The absorption spectra of the trimers (see Figure S59) were performed by TD-DFT calculations.

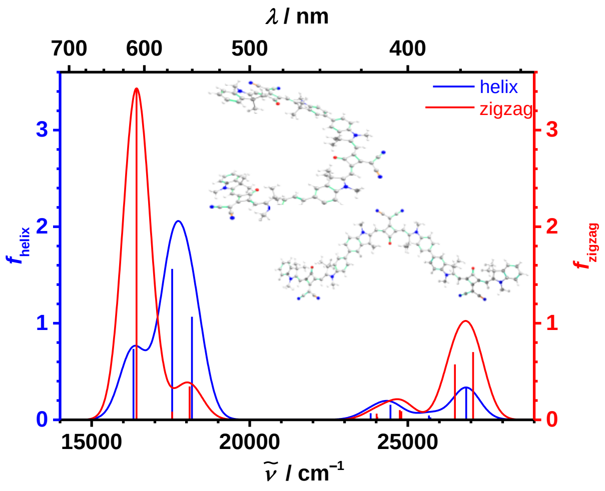

Figure S59. Absorption spectra of zig-zag and helix **SQ_3** at TD-DFT B3LYP/6-31G* level. Spectra were Gauss-broadened by 1000 cm^{-1}.

Force Field Calculations

SQ was parametrized for the CHARMM36 forcefield[14] using the FFTK toolkit[15] distributed with vmd 2.09[16] in conjunction with GAUSSIAN09[13] using the CAM-B3LYP[17] functional and def2-SVP basis[18] starting from related molecules like indole, which are readily parametrized in the latest CGENFF36 distribution.[19] All classical MD calculations were performed using NAMD2.12[20] as well as the latest CHARMM36 parameters and topologies for solvents and aliphatic tails. The simulations were performed using a 1 fs timestep at 300 K, a pressure of 1 atm and within periodic boundary conditions using Langevin dynamics[21] employing the Nose-Hoover-Langevin piston method.[22] The nonbonded pairlist distance was set to 14.0 A with 12.0 A cutoff distance and a switching distance of 10 A. The AM1 optimized geometry was used as a starting point and the solvent-(box) was freely equilibrated for 1 ns in a flexible cell with a fixed helix. The solvated system was then propagated for 100 ps. Along this trajectory every 10 ps the structure of the helix and solvent within roughly 10 A of the helix was extracted and optimized for 10000 steps to analyze the order and structure of the solvent molecules between the helix sheets.

7 References

[1] G. R. Fulmer, A. J. M. Miller, N. H. Sherden, H. E. Gottlieb, A. Nudelman, B. M. Stoltz, J. E. Bercaw, K. I. Goldberg, *Organometallics* **2010**, *29*, 2176-2179.

[2] a) S. F. Völker, T. Dellermann, H. Ceymann, M. Holzapfel, C. Lambert, *J. Polym. Sci., Part A: Polym. Chem.* **2014**, *52*, 890-911; b) N. Iwadate, M. Suginome, *J. Am. Chem. Soc.* **2010**, *132*, 2548-2549; c) H. Ceymann, A. Rosspeintner, M. H. Schreck, C. Mützel, A. Stoy, E. Vauthey, C. Lambert, *Phys. Chem. Chem. Phys.* **2016**, *18*, 16404-16413.
[3] L. Xu, P. Li, *Chem. Commun.* **2015**, *51*, 5656-5659.
[4] U. Mayerhöffer, M. Gsänger, M. Stolte, B. Fimmel, F. Würthner, *Chem. Eur. J.* **2013**, *19*, 218-232.
[5] D. J. Harkin, K. Broch, M. Schreck, H. Ceymann, A. Stoy, C. K. Yong, M. Nikolka, I. McCulloch, N. Stingelin, C. Lambert, H. Sirringhaus, *Adv. Mater.* **2016**, *28*, 6378-6385.
[6] G. A. Olah, S. C. Narang, B. G. B. Gupta, R. Malhotra, *J. Org. Chem.* **1979**, *44*, 1247-1251.
[7] M. H. Schreck, Doctoral dissertation thesis, Julius-Maximilians-Universität Würzburg (Würzburg), **2018**.
[8] M. D. Mohammadi, M. Hamzehloo, *Fluid Phase Equilib.* **2019**, *483*, 14-30.
[9] P. Lindner, R. Schweins, *Neutron News* **2010**, *21*, 15-18.
[10] B. E. Koene, D. E. Loy, M. E. Thompson, *Chem. Mater.* **1998**, *10*, 2235-2250.
[11] a) J. S. Pedersen, P. Schurtenberger, *Macromolecules* **1996**, *29*, 7602-7612; b) W.-R. Chen, P. D. Butler, L. J. Magid, *Langmuir* **2006**, *22*, 6539-6548.
[12] J. J. P. Stewart, 19.313W ed., Stewart Computational Chemistry.
[13] M. J. Frisch, G. W. Trucks, H. B. Schlegel, G. E. Scuseria, M. A. Robb, J. R. Cheeseman, G. Scalmani, V. Barone, G. A. Petersson, H. Nakatsuji, X. Li, M. Caricato, A. V. Marenich, J. Bloino, B. G. Janesko, R. Gomperts, B. Mennucci, H. P. Hratchian, J. V. Ortiz, A. F. Izmaylov, J. L. Sonnenberg, Williams, F. Ding, F. Lipparini, F. Egidi, J. Goings, B. Peng, A. Petrone, T. Henderson, D. Ranasinghe, V. G. Zakrzewski, J. Gao, N. Rega, G. Zheng, W. Liang, M. Hada, M. Ehara, K. Toyota, R. Fukuda, J. Hasegawa, M. Ishida, T. Nakajima, Y. Honda, O. Kitao, H. Nakai, T. Vreven, K. Throssell, J. A. Montgomery Jr., J. E. Peralta, F. Ogliaro, M. J. Bearpark, J. J. Heyd, E. N. Brothers, K. N. Kudin, V. N. Staroverov, T. A. Keith, R. Kobayashi, J. Normand, K. Raghavachari, A. P. Rendell, J. C. Burant, S. S. Iyengar, J. Tomasi, M. Cossi, J. M. Millam, M. Klene, C. Adamo, R. Cammi, J. W. Ochterski, R. L. Martin, K. Morokuma, O. Farkas, J. B. Foresman, D. J. Fox, Gaussian, Inc., Wallingford, CT, **2013**.
[14] B. R. Brooks, R. E. Bruccoleri, B. D. Olafson, D. J. States, S. Swaminathan, M. Karplus, *J. Comput. Chem.* **1983**, *4*, 187-217.
[15] C. G. Mayne, J. Saam, K. Schulten, E. Tajkhorshid, J. C. Gumbart, *J. Comput. Chem.* **2013**, *34*, 2757-2770.
[16] W. Humphrey, A. Dalke, K. Schulten, *J. Mol. Graph.* **1996**, *14*, 33-38.
[17] T. Yanai, D. P. Tew, N. C. Handy, *Chem. Phys. Lett.* **2004**, *393*, 51-57.
[18] a) F. Weigend, R. Ahlrichs, *Phys. Chem. Chem. Phys.* **2005**, *7*, 3297-3305; b) F. Weigend, *Phys. Chem. Chem. Phys.* **2006**, *8*, 1057-1065.
[19] K. Vanommeslaeghe, E. Hatcher, C. Acharya, S. Kundu, S. Zhong, J. Shim, E. Darian, O. Guvench, P. Lopes, I. Vorobyov, A. D. Mackerell, Jr., *J. Comput. Chem.* **2010**, *31*, 671-690; J. Comput. Chem.
[20] J. C. Phillips, R. Braun, W. Wang, J. Gumbart, E. Tajkhorshid, E. Villa, C. Chipot, R. D. Skeel, L. Kalé, K. Schulten, *J. Comput. Chem.* **2005**, *26*, 1781-1802; J. Comput. Chem.
[21] D. Quigley, M. I. J. Probert, *J. Chem. Phys.* **2004**, *120*, 11432-11441.
[22] a) G. J. Martyna, D. J. Tobias, M. L. Klein, *J. Chem. Phys.* **1994**, *101*, 4177-4189; b) S. E. Feller, Y. Zhang, R. W. Pastor, B. R. Brooks, *J. Chem. Phys.* **1995**, *103*, 4613-4621.

Author Contributions

A.T. performed the synthesis, absorption based and NMR characterisation and wrote the original draft. M.H. performed the semiempirical and DFT calculations. M.A. performed and interpreted

the SANS measurement. D.F. performed the force field calculations. R.M. supervised the force field calculations, F.G supervised the SANS experiment. C.L. designed the project, acquired the funding and co-wrote the paper. All authors contributed to the interpretation of the results and commented on the manuscript.
